# Major Shifts in Glial Regional Identity Are a Transcriptional Hallmark of Human Brain Aging

**DOI:** 10.1016/j.celrep.2016.12.011

**Published:** 2017-01-10

**Authors:** Lilach Soreq, UK Brain Expression Consortium, North American Brain Expression Consortium, Jamie Rose, Eyal Soreq, John Hardy, Daniah Trabzuni, Mark R. Cookson, Colin Smith, Mina Ryten, Rickie Patani, Jernej Ule

**Affiliations:** 1 Institute of Neurology, University College London, London WC1N 3BG, UK; 2 The Francis Crick Institute, 1 Midland Road, London NW1 1AT, UK; 3 MRC Edinburgh Brain Bank, Academic Neuropathology, Centre for Clinical Brain Sciences, University of Edinburgh, Edinburgh EH16 4SB, UK; 4 The Computational, Cognitive and Clinical NeuroImaging Laboratory, Division of Brain Sciences, Imperial College, London SW7 2AZ, UK; 5 Reta Lila Weston Institute of Neurological Studies, UCL ION, 1 Wakefield Street, London WC1N 1PJ, UK; 6 Departments of Genetics, King Faisal Specialist Hospital and Research Centre. Riyadh 12713, Saudi Arabia; 7 Laboratory of Neurogenetics, National Institute on Aging, NIH, Bethesda, MD 20892, USA; 8 Euan MacDonald Centre for MND, University of Edinburgh, Edinburgh EH8 9YL, UK; 9 Department of Medical and Molecular Genetics, King’s College London, Guy’s Hospital, Great Maze Pond, London SE1 9RT, UK; 10 Department of Clinical Neurosciences, University of Cambridge, Cambridge CB2 1TN, UK

**Keywords:** aging, gene expression, machine learning, immunohistochemistry, brain, neurons, olgiodendrocytes, microglia, exon microarrays, RNA-seq

## Abstract

Gene expression studies suggest that aging of the human brain is determined by a complex interplay of molecular events, although both its region- and cell-type-specific consequences remain poorly understood. Here, we extensively characterized aging-altered gene expression changes across ten human brain regions from 480 individuals ranging in age from 16 to 106 years. We show that astrocyte- and oligodendrocyte-specific genes, but not neuron-specific genes, shift their regional expression patterns upon aging, particularly in the hippocampus and substantia nigra, while the expression of microglia- and endothelial-specific genes increase in all brain regions. In line with these changes, high-resolution immunohistochemistry demonstrated decreased numbers of oligodendrocytes and of neuronal subpopulations in the aging brain cortex. Finally, glial-specific genes predict age with greater precision than neuron-specific genes, thus highlighting the need for greater mechanistic understanding of neuron-glia interactions in aging and late-life diseases.

## Highlights

• Understanding the role of cell-type-specific changes in human brain aging
• Glial-specific genes shift their regional expression patterns during aging
• Oligodendrocytes and neuronal subpopulations are decreased in the aging neocortex
• Microglia-specific genes globally increase their expression during aging

## Graphical Abstract

**Figure:**
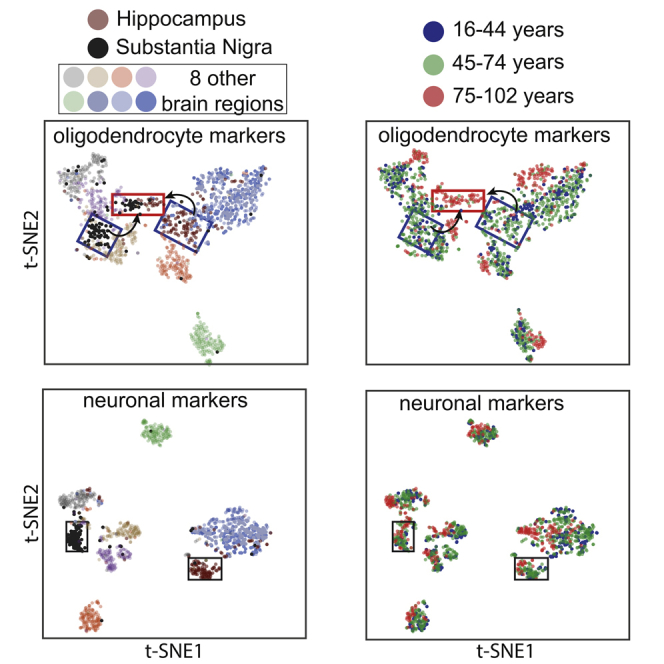

## Teaser

Human brain aging is determined by a complex interplay of regional and cell-type-specific molecular events. Soreq et al. find that glial genes shift their regional expression patterns, while microglia-specific genes globally increase their expression upon aging. Moreover, immunohistochemistry reveals decreased numbers of oligodendrocytes and neuronal subpopulations in the aging neocortex.

## Introduction

Aging, an inevitable time-dependent functional decline, is present in all living organisms. The intimate relationship between aging and neurodegeneration raises the possibility of shared transcriptional and post-transcriptional gene regulation programs; however, we still lack a comprehensive transcriptome-wide picture of the effects of aging across different human brain regions and cell types (De Strooper and Karran, 2016). RNA expression profiling of the aging brain has been studied historically using a limited number of brain regions in animal models or human post-mortem tissues. A major unrealized goal therefore remains a comprehensive characterization of the transcriptional landscape across multiple human brain regions in a physiological age range, which may provide insights into the cellular architecture and molecular pathways of aging.

The unparalleled complexity of the human brain is a function of its structural and functional cellular diversity, which arises from tightly regulated transcriptional programs. Limited availability to human post-mortem samples has hampered comprehensive transcriptomic analysis of the brain, particularly of region- and cell-type-specific diversity. However, through international collaboration, a comprehensive atlas of the brain’s transcriptome based on samples from two individuals (the Allen Brain Atlas) has been achieved. This study illustrated how transcripts of genes involved in different pathways are expressed across the brain, but the potential effect of age on the regional differences was not examined.

By current consensus, astrocyte (AC) and neuronal numbers appear generally preserved in aging (Fabricius et al., 2013, Matarin et al., 2015, Pelvig et al., 2008). It is clear, however, that Alzheimer’s disease (AD) and other neurodegenerative diseases for which age is a major risk factor are associated with inflammatory changes mediated by microglia (MG) (Cribbs et al., 2012, Frank et al., 2008). Brain aging includes accumulation of senescent MG, altered signaling, and pro-inflammatory phenotypes (Mosher and Wyss-Coray, 2014), and it was shown that MG display regional sensitivity to aging (Streit and Xue, 2010). Immune-related changes were also strongly associated with aging in mouse models of amyloid pathology (Matarin et al., 2015). Nevertheless, animal models and human tissue have reported variable and apparently contrasting alterations in ACs (reactivity or atrophy) and MG (MHC class II antigen increase or atrophy) (Cerbai et al., 2012, Streit and Xue, 2010, Tremblay et al., 2012). Accumulation of oligodendrocytes (OLGs) was previously reported in aging monkey cortex (Peters and Sethares, 2004), while stereological quantification of glia in neocortical regions of old brains has suggested a reduction in the number of OLGs, as evident by a >3-fold greater atrophy of the sub-cortical white matter (WHMT) compared to cortical regions and an age-determined loss of myelin (Head et al., 2004, Vernooij et al., 2008). Furthermore, MG-mediated neuroinflammation has been described as a common hallmark of both AD and Parkinson’s disease (PD) and is believed to be mechanistically important in driving pathogenesis (Orre et al., 2013, Perry and Teeling, 2013). Collectively, these findings suggest that the field stands to benefit from systematic and comprehensive analysis of aging-related changes in the cellular and molecular composition of the human brain.

Apart from the study of region-dependent microglial response to aging, the importance of both region- and cell-type-specific changes in the aging brain remains poorly understood. Studies have been hampered by the limited availability of cross-regional post-mortem tissue across a range of ages. To overcome these limitations, we analyzed gene expression patterns in ten brain regions (including cortical and sub-cortical areas) using more than 1,800 brain samples from two large independent cohorts, representing the most comprehensive human aging brain gene expression analysis to date. We report striking changes in cell-type-specific expression patterns across different brain regions, which revealed major shifts in glial regional identity upon aging in the human brain.

## Results

**Figure 1:**
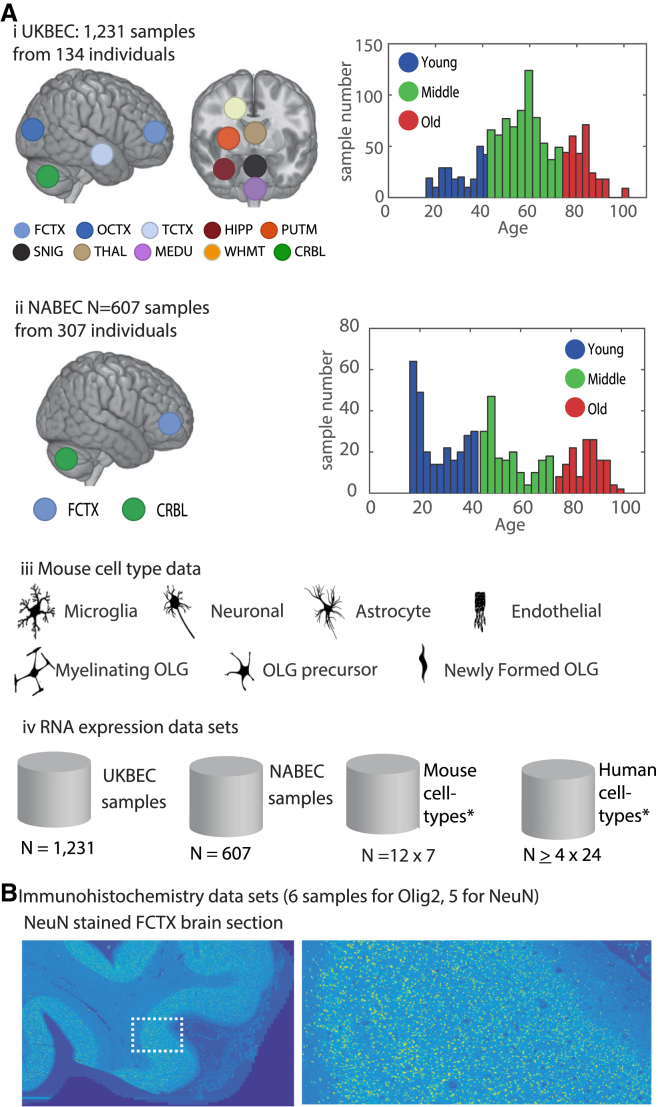
Analyzed Samples and Datasets (A) The samples of the UKBEC and NABEC datasets were divided into three age groups each (young: 16–44, middle: 45–74, old: ≥75). (i) The main analyzed dataset (UKBEC) is composed of 1,231 brain samples interrogated by exon microarrays, from brain samples of 134 individuals from 16 to 102 years old and up to ten brain regions each. The brain regions included both cortical and sub-cortical regions, specifically: the frontal cortex (FCTX), temporal cortex (TCTX), occipital cortex (OCTX), intralobular white matter (WHMT), cerebellum (CRBL), substantia nigra (SNIG), putamen (PUTM), thalamus (THAL), hippocampus (HIPP), and medulla (MEDU) for UKBEC and the FCTX and CRBL for NABEC. (ii) The independent (NABEC) dataset of brain samples from FCTX and CRBL 307 individuals (16–101 years old). (iii) In addition, seven cell types were identified based on analysis of available RNA-seq data from mice cortex (http://web.stanford.edu/group/barres_lab/brain_rnaseq.html). (iv) A summary of all expression data used in this study. The total number of samples described in (i)–(iii) is listed, as well as the human RNA-seq analysis of 24 CNS human cell types (Table S7) (http://web.stanford.edu/group/barres_lab/brainseqMariko/brainseq2.html). (B) High-resolution immunohistochemical imaging dataset was produced from samples of young and three old FCTX from the UKBEC cohort, following staining by OLIG2 antibody and computational analysis for the quantification of the OLG cell population. Staining by NeuN of FCTX sections from the same brain samples followed by targeted computational analysis was conducted for quantification of the neuronal cell population (an example of one of the NeuN stained sections is shown on the right, in the zoomed-in view of the area marked on the left-hand side). OLG, oligodendrocyte.

In this study, we examined two extensive gene expression datasets from post-mortem human samples and sampled multiple (up to ten) brain regions per individual. The primary dataset was produced by the UK Brain Expression Consortium (UKBEC) and included 1,231 tissue samples collected from 134 adult individuals between 16 and 102 years old, with each contributing post-mortem samples of up to ten brain regions (Figure 1Ai). The brain regions included both cortical and sub-cortical regions, specifically the frontal cortex (FCTX), temporal cortex (TCTX), occipital cortex (OCTX), intralobular white matter (WHMT), cerebellum (CRBL), substantia nigra (SNIG), putamen (PUTM), thalamus (THAL), hippocampus (HIPP), and medulla (MEDU). The second dataset, which allowed independent external cross-validation, was produced by the North American Brain Expression Consortium (NABEC) (Gibbs et al., 2010, Kumar et al., 2013), including 307 samples from two brain regions (age range: 16 to 101 years old; Figure 1Aii). The third dataset was also used for validation including samples with an age range of 27 to 106 years old (Lu et al., 2014). None of the brain samples had neuropathological evidence of diagnosable degenerative diseases (Table S1). To detect differentially expressed genes, we assigned each sample to one of three age groups (young: 16–44, middle: 45–74, old: ≥75 years old) and applied a collection of tailored data-mining computational approaches (Figure 1). We excluded gender-based sample separation to specifically identify the effects of age on gene expression profiles.

### Region-Specific and Global Transcriptional “Signatures” of the Aging Human Brain

**Figure 2:**
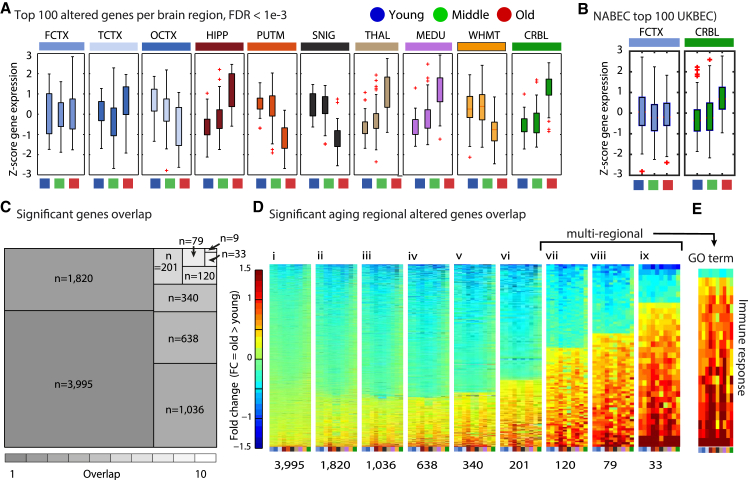
Multi-regional Aging-Altered Genes Are Mainly Upregulated (A) The direction of expression change of the top 100 genes detected as significantly differentially expressed upon aging in each of the studied expression datasets from ten UKBEC brain regions (ANOVA test significance threshold: FDR < 1e−3; the test compared the three defined age groups). (B) Age-group based separation of 607 FCTX and CRBL samples (the NABEC cohort) was based on measured expression of the nine cross-regional genes. (C) A tree map of the number of genes that were altered upon aging, dependent on the number of brain regions where the change is observed. (D) Fold change of the genes that were altered upon aging, separated into heatmaps dependent on the number of brain regions where the change is observed. (E) Fold change of the multi-regional genes that were enriched in the Gene Ontology term immune response (standardized *Z* score; range is as shown for the heatmaps on the left). Brain region abbreviations are explained in the legend to Figure 1A. See also Figure S1A for the total number of aging-altered genes per region.

We first sought to address whether region-specific differences in gene expression patterns occur within the brain upon aging. Both the number of differentially expressed genes (threshold: false discovery rate [FDR] < 1e−3) (Figure S1) and the direction of expression change varied in a region-specific manner (Figure 2A). The general directions of gene expression change were preserved in the independent NABEC dataset (Figure 2B). We applied a stringent threshold to enable isolation of global changes across the UKBEC brain regions. Most changes were specific for one region (hereafter referred to as “region specific”) or a few regions (hereafter referred to as “region selective,” including genes altered in two to seven regions), while some genes were altered in eight or more brain regions (hereafter referred to as “multi-regional”), and nine genes were found to be significantly altered in all ten brain regions upon aging (FDR < 1e−3, hereafter referred to as “cross-regional”) (Figure 2C; Figure S2). The rates and number of overlapping age-altered genes varied between pairs of brain regions (Figure S2). Multi-regional genes predominantly exhibited increased expression levels upon aging (Figures 2Dviii–2Dix). This group of genes was enriched in the Gene Ontology (GO) term “immune response”, which had the general trend to be upregulated in aging (Figure 2E).

### Nine Cross-Regional Aging-Altered Genes Accurately Predict the Age Categories

**Figure 3:**
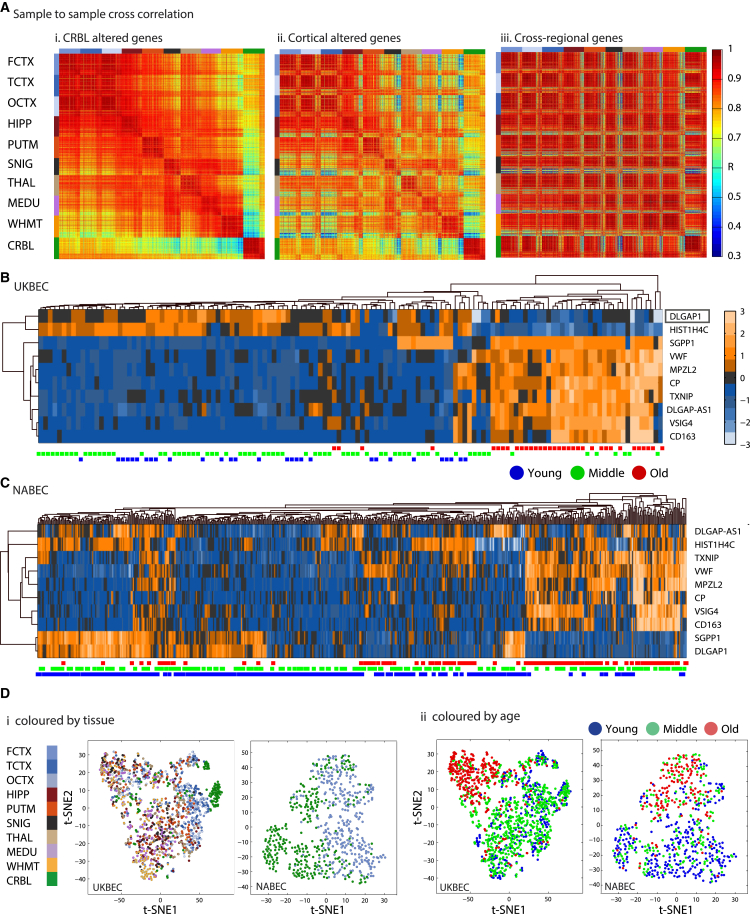
The Nine Cross-Regional Genes Discriminate Samples Based on Age (A) Correlation scores were calculated between each pair of brain samples among the UKBEC samples based on different lists of aging-altered genes using Spearman correlation. (i) Correlation scores based on CRBL aging-altered genes. (ii) Correlation scores based on cortical aging-altered genes. (iii) Correlation scores based on cross-regional aging-altered genes. See Figure S1B for correlation based on WHMT-altered genes. (B) Hierarchical classification of the UKBEC cohort based on the expression signals of the cross-regional altered genes and the *DLGAP* antisense. Rows, genes; columns, samples. Age group is denoted in blue (young: 16–44 years), green (middle-age: 45–74 years), or red (old: ≥75 years). The dendrograms show the Euclidian distance measured for both rows and columns. Right color bar, standardized fold change (*Z* score; range: −3 to 3; orange represents increased expression in aging, and blue denotes decrease). (C) Hierarchical classification of the NABEC expression dataset based on the profiles of the cross-regional genes and the DLGAP antisense. (D) Non-linear dimensionality reduction by t-distributed stochastic neighbor embedding (t-SNE) is based on the expression of the nine cross-regional genes, with the x axis showing t-SNE1 and the y axis showing t-SNE2. Either the ten UKBEC brain regions or the two NABEC brain regions (FCTX and CRBL) are classified, as marked in the plots. (i) Each sample is colored based on its corresponding tissue (colors are marked on the left). (ii) The same samples are colored based on their age group (colors are marked on the top). Brain region abbreviations are explained in the legend to Figure 1A.

To assess the ability of different groups of genes to classify the brain samples in both cohorts by brain region and age group criteria, we next used a non-linear dimension reduction classification method. The expression of regional-selective genes separated the samples well based on their regional identity (Figure S2). In addition, genes with aging-altered expression patterns in the CRBL, WHMT, or cortical samples generally have distinctive regional expression, as evident by sample-to-sample correlation scores that were computed among each of the 1,231 brain samples based on their expression signals (Figures 3Ai and Aii; Figure S1B). The 642 CRBL-altered genes showed high inter-regional correlation in expression patterns with the cortical regions (including the HIPP), the 265 cortical aging-altered genes revealed high correlation among the cortical regions and HIPP samples, and the 801 WHMT-altered genes showed increased correlation specifically within CRBL and WHMT.

In contrast to region-specific aging-altered genes, expression patterns of these cross-regional genes were correlated among samples only based on age, rather than brain region (Figure 3Aiii). Eight of the pan-regional genes were upregulated, and one (*HIST1H4C*) was downregulated upon aging (Figure 3B; Table S5). The cross-regional genes successfully discriminated the samples based on age group in most UKBEC cases (130 of 134) (Figure 3B). One of these genes was a non-coding RNA (*DLGAP1-AS1*) that is antisense to the protein-coding gene (*DLGAP1*). We identified a robust, reciprocal relationship in expression between *DLGAP1* and *DLGAP1-AS1* upon aging. *DLGAP1* is highly brain-specific, while *DLGAP1-AS1* and *DLGAP1-AS2* are normally expressed in internal organs and the bone marrow (Gene Cards database) (Harel et al., 2009). We find that in contrast to upregulation of *DLGAP1-AS*, expression of *DLGAP1* shows an aging-altered decrease across brain regions, despite not reaching statistical significance in each region (Figure 3B). This demonstrates coupling between the age-dependent decrease in the expression of the brain-specific protein-coding gene DLGAP1 and the increase of its antisense RNA, which is otherwise only expressed outside of the brain.

As a sign of the validity of the cross-regional genes, they were efficient in classifying samples of the independent NABEC dataset based on age, even though the NABEC data were not used to identify these genes (Figure 3C). Moreover, a non-linear classification based on these genes separated samples belonging to young, middle-age, and old groups in the UKBEC, the NABEC, and an additional independent FCTX brain expression cohort (Lu et al., 2014) (Figure 3D; Figure S3C). This model also verified the prediction of age group based on expression levels in the NABEC cohort (Figure S3A). To exclude the effects of other variables, we show that the UKBEC samples were not classified by gender (Figure S3B). The cross-regional genes also correctly classified samples by age group in an additional independent dataset of cortical samples (Lu et al., 2014; Figure S3) and did not classify the UKBEC dataset by gender (Figure S3).

### Major Shifts in Region-Specific Expression Profiles of Glia-Specific Genes in the Aging Brain

To investigate the biological relevance of the age-related gene expression changes, we first examined the expression profiles of the cross-regional genes in recently produced RNA sequencing (RNA-seq) data from seven purified mouse brain cell types (Zhang et al., 2014). All cross-regional genes were expressed in a cell-type-specific manner, in particular within glial cells and mainly in MG and OLGs (Figure S4A). We therefore further examined the cell-type-specific expression patterns of all aging-altered genes. For this, we calculated genome-wide expression scores to identify genes specific for each cell type (Table S3). We then examined whether expression of cell-type-specific genes was altered upon aging. For three cell types (neurons, ACs, and OLGs), enrichment data previously generated by microarrays was also available (Cahoy et al., 2008). We selected genes that were demonstrated to be specific by both our defined cell-specific lists found by analysis of RNA-seq data (Zhang et al., 2014) and the published microarray cell-specific lists from mice (Cahoy et al., 2008), in addition to being altered in aging. In agreement with the great diversity of neuronal cell types across different brain regions, neuron-specific genes were most enriched among the regional aging-altered genes (Figure S4B). In contrast, glia-specific genes were most enriched among the multi- and cross-regional altered genes, and this was most pronounced for OLG precursors and MG (Figure S4B).

**Figure 4:**
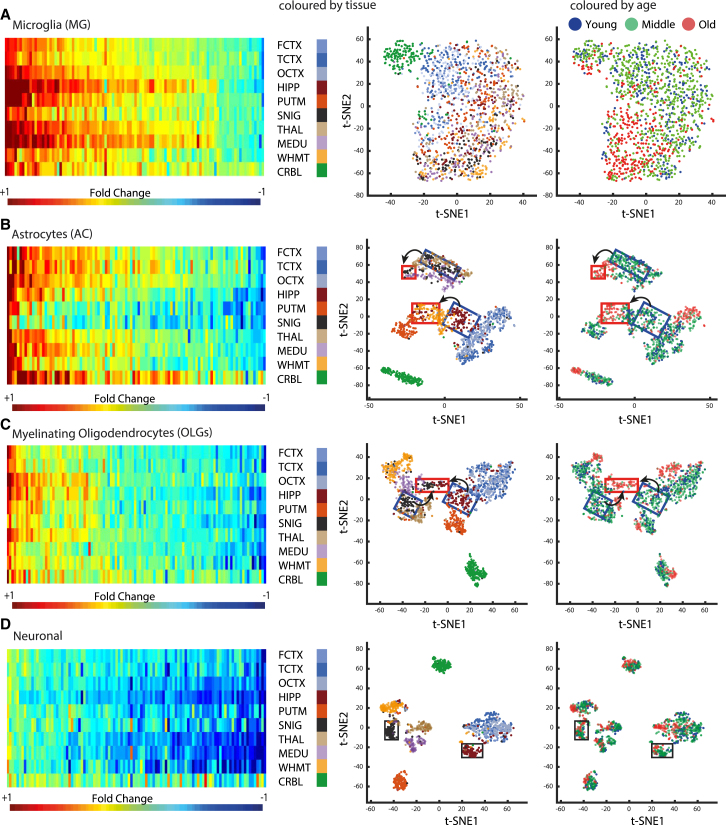
Glia-Specific Genes Show Major Shifts in Regional Identity upon Aging On the left, heatmaps show the fold change between old and young groups in the expression of the top 100 aging-altered cell-type-specific genes across regions (the color bar corresponds to the standardized *Z* score, with blue corresponding to decrease and red to increase in gene expression; range: −1 to +1). On the right, non-linear dimensionality reduction by t-distributed stochastic neighbor embedding (t-SNE) is used to classify a sample of the ten UKBEC brain regions based on the expression of the top 20 aging-altered cell-specific genes, with the x axis showing t-SNE1 and the y axis showing t-SNE2. In the first plot, each sample is colored based on its corresponding tissue (colors are marked on the left of the plot), and in the second plot, the same samples are colored based on their age group (colors are marked on the top of the plots). (A) Sample classification based on the aging-altered MG-specific genes. (B) Sample classification based on the aging-altered AC-specific genes. (C) Sample classification based on aging-altered myelinating OLG-specific genes. (D) Sample classification based on the expression signals of aging-altered neuron-specific genes. The SNIG and PUTM samples are marked by rectangles as an example of the loss of region-specific expression upon aging for OLG- and AC-specific genes. Brain region abbreviations are explained in the legend to Figure 1A. MG, microglia; AC, astrocyte; OLG, oligodendrocyte. See Figure S2 for sample classification based on region-specific genes compared with multi-regional genes and Figure S6 for heatmaps and classification plots based on the three cell-type microarray gene markers.

We first sought to investigate the changes in expression of MG-specific genes, because these cells have been most extensively linked to aging so far (Erraji-Benchekroun et al., 2005, Sibille, 2013). Consistent with previous studies (Erraji-Benchekroun et al., 2005, Sibille, 2013), most MG-specific aging-altered genes had low expression in brain samples from the young group but strongly increased their expression in all regions in the old group samples (Figure S5A). Our study extends these findings to a multi-regional phenomenon. Moreover, we find that a small number of MG-specific genes have high absolute expression in the young group but decreased expression upon aging in all regions. This suggests that the change in expression not only reflects an increased number of MG but most likely also includes a dramatic change in the MG gene expression program. Although most brain regions were not strongly separated by the MG-specific aging-altered genes, the CRBL was distinct (Figure 4A). This complies with a report of a distinct MG expression profile in the CRBL of young mice (Grabert et al., 2016). Apart from the CRBL samples that formed a separate cluster, samples from the old group clustered together for all other regions and separately from younger samples, indicating that MG-specific gene expression is more defined by age than by regional identity (Figure 4A). The aging-altered endothelial-specific genes showed a similar pattern of changes as the MG-specific genes, with a general upregulation across all brain regions and a notable age group separation and lack of clear regional identity (Figure S5D).

Next, we examined the expression profiles of genes specific for either ACs or OLGs. In young samples, we observed much higher absolute expression of AC-specific genes in the midbrain regions compared to cortex and HIPP (Figure S5). However, AC-specific genes increase their expression within cortical regions and exhibit decreased expression in basal ganglia (BG) upon aging (Figure 4B); therefore, their absolute expression signals become more similar across regions in the aging brain (Figure S5A). SNIG and THAL, which show the highest expression of AC-specific genes in the young brain, have a generic decrease of AC-specific genes upon aging (Figure 4B). In contrast, the AC-specific genes with the lowest expression in the young group increase their expression upon aging in all regions except SNIG and THAL. This leads to remarkable shifts in the regional patterns of AC-specific gene expression. Although expression of AC-specific genes clustered most brain regions separately for the young group, only four regional clusters remained in the old group, two of which were the CRBL and the cortical regions (Figure 4B). The most pronounced change is seen for HIPP and SNIG. For example, HIPP clusters close to cortex in the young group but shifts toward the WHMT and PUTM in the samples from the old group (Figure 4B).

The aging-altered genes that are specific for all stages of OLG differentiation, including OLG precursors, newly formed OLGs, and myelinating OLG generally show a trend toward decreased expression in all regions upon aging (Figures S5A–S5C). Moreover, OLG-specific genes show a shift of region-specific gene expression upon aging, with the strongest change of regional identity seen in HIPP and SNIG. In the samples from the young group, HIPP clusters close to cortical samples and SNIG clusters close to MEDU and THAL, whereas in the samples from the old group, HIPP and SNIG cluster closer to each other (Figure 4C).

Similar to the OLG-specific genes, the aging-altered neuron-specific genes showed predominant downregulation in all brain regions upon aging (Figure D; Figure S5B), in agreement with previous studies that observed decreased expression of neuron-specific genes in the cortex (Erraji-Benchekroun et al., 2005), but with the added insight that this occurs in a brain-wide manner. Classification based on aging-altered neuron-specific genes yielded a striking separation of samples based on their regional identity (Figure 4D), and the old group samples remained clustered closest to the young samples of the same brain region (Figure 4D). Thus, neuron-specific gene expression is more defined by regional identity than by age. This agrees with the finding that the downregulated genes, which are often neuron specific, are also generally region-specific, while the upregulated genes, which are often MG specific, are generally multi-regional (Figure 2D; Figure S5B).

### Specific Neuronal Subpopulations and Oligodendrocytes Are Decreased in the Aging Brain

To examine how gene expression changes may relate to changes in brain cell populations, we developed an efficient pipeline for analysis of high-resolution image tiles of immunolabeled sections of FCTX. We developed a targeted computational pipeline for detection and quantification of the stained cells based on the scanned images, which consists of big data detection, segmentation, and quantification pipeline using thresholding, filtering, and object detection.

**Figure 5:**
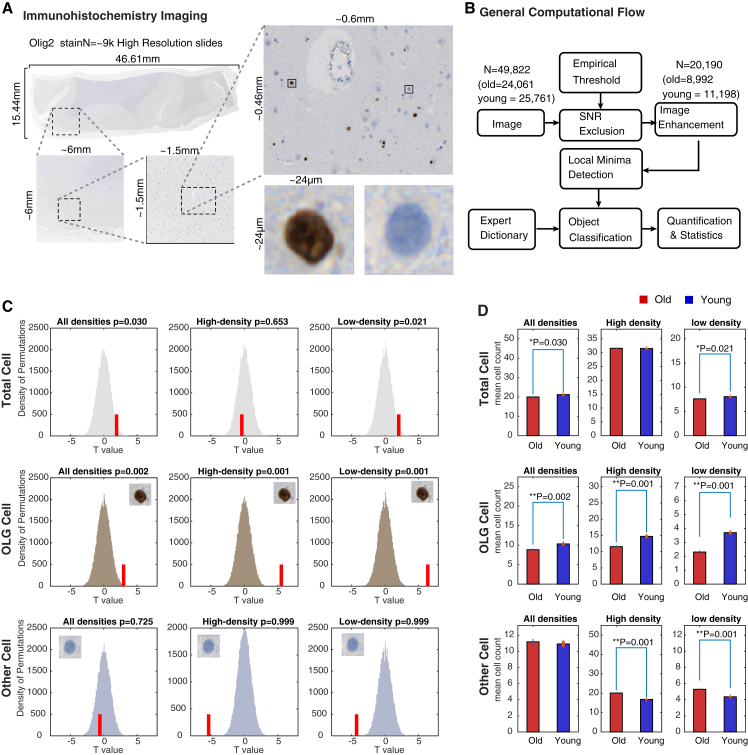
Decreased Counts of Oligodendrocytes in the Frontal Cortex upon Aging Six FCTX brain sections were stained and imaged (from three old and three young post-mortem brain samples). Each sample contains thousands of equal-size slides each 1,600 × 1,200 pixels, as captured by a Zeiss AxioScan slide scanner following staining with the Olig2 antibody. (A) An example of a BA9-Olig2 slide shown in a full-resolution pyramid, with gradual zooming into two typical cells: one stained brown (OLG cells) and one stained blue (other cells). (B) General computational pipeline for the analysis of high-resolution immunohistochemical high-dimensional imaging data allowed us to quantify both OLG and other cells in each FCTX slide. (C) Comparison of OLG counts that asks if the number of cells of interest is different in young samples compared to old (i.e., red bar shifted to the right means increased count in young samples). In each panel, the histogram represents the null distribution of t values calculated using two-tailed Student’s t test over slide cell counts randomly sampled from the entire population of the six samples, using 100 random iterations over 500 permutations where the true-label t statistics is depicted with a red bar, and the remaining distribution was calculated based on shuffled labels. The analysis was done on overall 8,766 young and 10,922 old group slides (left). From a total of 2,612 young and 1,828 old group high-density slides, the 50 slides with the highest density were selected per case for quantification. Similarly, from 1,154 young and 1,277 old group low-density slides, the 50 slides with the lowest density were chosen per sample for quantification. (D) Cell counts in samples from old (red) and young (blue) groups, with significance calculated with t statistics as described in (C). The star marks bars with a p value < 0.05 and the mean T statistic, p value and SD of the permutation test are reported on top of the graphs.

Noting the trend for decreased expression of OLG-specific genes in the frontal cortex (Figure 4D), we examined serial sections immunolabeled with OLIG2 antibody from the tissue blocks from the same brain samples that were used for the microarray study. We selected three young and three old cases based on their microarray profiles, such that it was representative for their age (see Supplemental Experimental Procedures). Approximately 50,000 image tiles were analyzed from the three young and three old FCTX sections (Figure 5A). We counted the number of OLIG2-positive cells compared to the total number of cell nuclei in each tile. Statistics was calculated on two classes of tile density, likely corresponding to local variations in the proportion of white matter (low density of nuclei) and gray matter (high density of nuclei), in addition to all densities combined (all tiles). The number of OLIG2-positive cells decreased in all classes of tiles in the aging FCTX, with the largest decrease in the low-density tiles in old compared to young cases (Figure 5C, middle panel). In contrast, the number of other cells significantly increased low-density tiles (Figure 5C, lower panel), in agreement with the slight increase in the total number of cells in the same tiles (Figure 5C, upper panel). This analysis demonstrates that the decreased expression of OLG-specific genes might partly reflect a decrease in three cortical OLG cell population.

**Figure 6:**
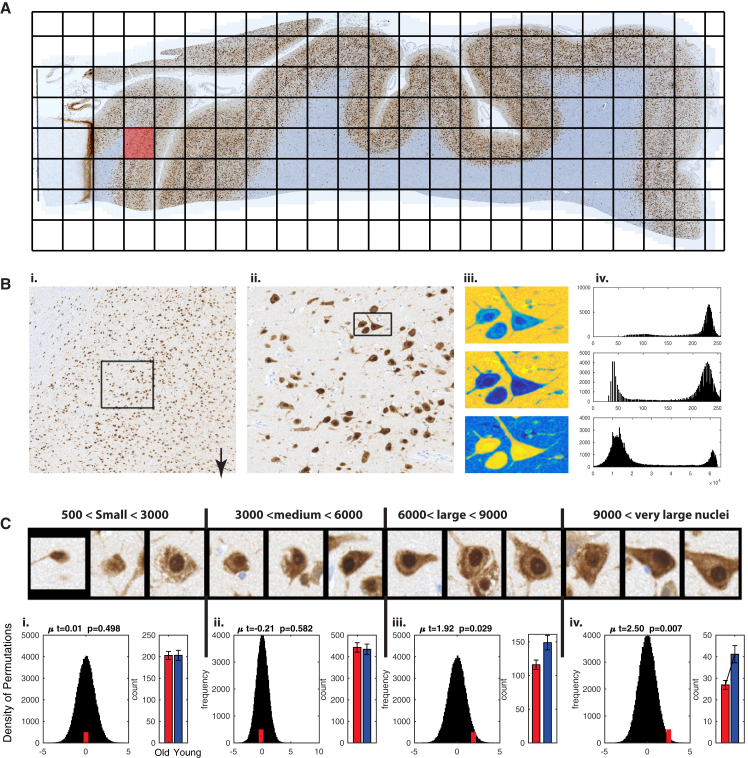
Decreased Counts of Specific Neuronal Populations in the Frontal Cortex upon Aging (A) An image of one NeuN-stained FCTX section, with re-defined tiles demonstrated by black rectangles (file size = 37.4 GB). (B) (i) Enlargement of a single tile of 10,000 × 10,000 pixels (size = 225 MB). (ii) Enlargement of a 2,500 × 2,500 pixel section. (iii) Three cells as observed in the red channel (top, shown in light blue) and blue channel (middle), and intersection of the two channels (bottom) differentiates between neuronal cells (stained by NeuN in brown on the original slides) and other cells (stained by Heamotoxylin in blue in the middle plot). (iv) The x axis represents the color frequency distribution of the red and blue channels across an intensity range of 256 gray levels, while the y axis represents the frequency of pixel intensity in the image tile depicted in (iii). (C) Examples of detected neurons that contain small, medium, medium to large (E), or large cell body (F) with size given in pixels. Underneath each image is the histogram that asks if the number of cells of interest is different in young samples compared to old (i.e., red bar shifted to the right means increased count in young samples). The histogram shows the null distribution of t values, calculated using two-tailed Student’s t test over slide counts using 100,000 random permutations from the entire population of the six samples (black bars), while the mean of the true-label t statistics is depicted with a red bar. The right graph shows the cell counts in samples from old (red) and young (blue) groups, with significance calculated with t statistics based on 10,000 random permutations. The star marks bars with a p value < 0.05, and the mean t statistics, p value, and SD of the permutation test are reported on top of the graphs.

The aging RNA expression signatures also revealed downregulation of neuron-specific genes (Figure 4B); therefore, we analyzed high-resolution images produced from the three young and three old FCTX sections stained with NeuN antibody to mark the neurons. This antibody detects the neuron-specific RNA-binding protein RBFOX3, which is predominantly nuclear, but is also present in the cytoplasm of the cell body (Kim et al., 2009) (Figure 6A). We used the tissue samples from the same cases as were used for OLIG2 quantification, thus allowing direct comparison of the two cell types. To capture the large diversity of both shape and size of cell bodies in the neuronal populations, we used a large tile size (10,000 × 10,000 pixels each) (Figure 6Bi). This allowed us to extract information from almost all layers of the neocortex in each slide of gray matter. A preliminary quality control analysis flagged one image from a young individual as a technical outlier, and this sample was therefore omitted from further analysis, although we provide access to its data (https://figshare.com/s/f2675361af1242f3565f). We processed 1,044 image tiles using our cell detection pipeline and applied an information exclusion criterion (entropy > 5) to contain the most meaningful slides (n = 641). In an attempt to enrich the regions of gray matter with the highest information content, we further focused on the 184 tiles with the highest density of nuclei. A total of 371,096 neurons were identified. We further segregated cells into four bins of total area of cell body (small: 500–3,000 pixels, n = 92,947; medium: 3,000–6,000 pixels, n = 202,239; large: 6,000–9,000 pixels, n = 60,314; very large: >9,000 pixels, n = 15,596). In agreement with the previous study (Kim et al., 2009), the intensity of cytoplasmic NeuN signal was strongest in the largest cells (Figure 6C). To account for the imbalance of tiles across samples, significance in the age-dependent decrease in each neuronal population was tested with right-tailed two-sample t test. We observed no change in the number of neurons with small or medium-size cell bodies, which represent 80% of detected neurons. In contrast, the number of neurons with large or very large cell bodies is significantly decreased (p = 0.029 and p = 0.007, accordingly; right-tailed two-sample t test) Figure 6C).

Altogether, it is likely that changes in gene expression observed in our study reflect a combination of changes in expression profiles and changes in quantity of specific cell types. While most neuron-specific genes are predominantly downregulated, the extent of this downregulation varies among brain regions. All other cell types have a more complex pattern of changes. These changes appear most pronounced in HIPP and SNIG, which show the strongest shifts in the regional expression pattern of AC- and OLG-specific genes upon aging.

### MG- and Endothelial-Specific Genes Are the Best Predictors of Biological Age

**Figure 7:**
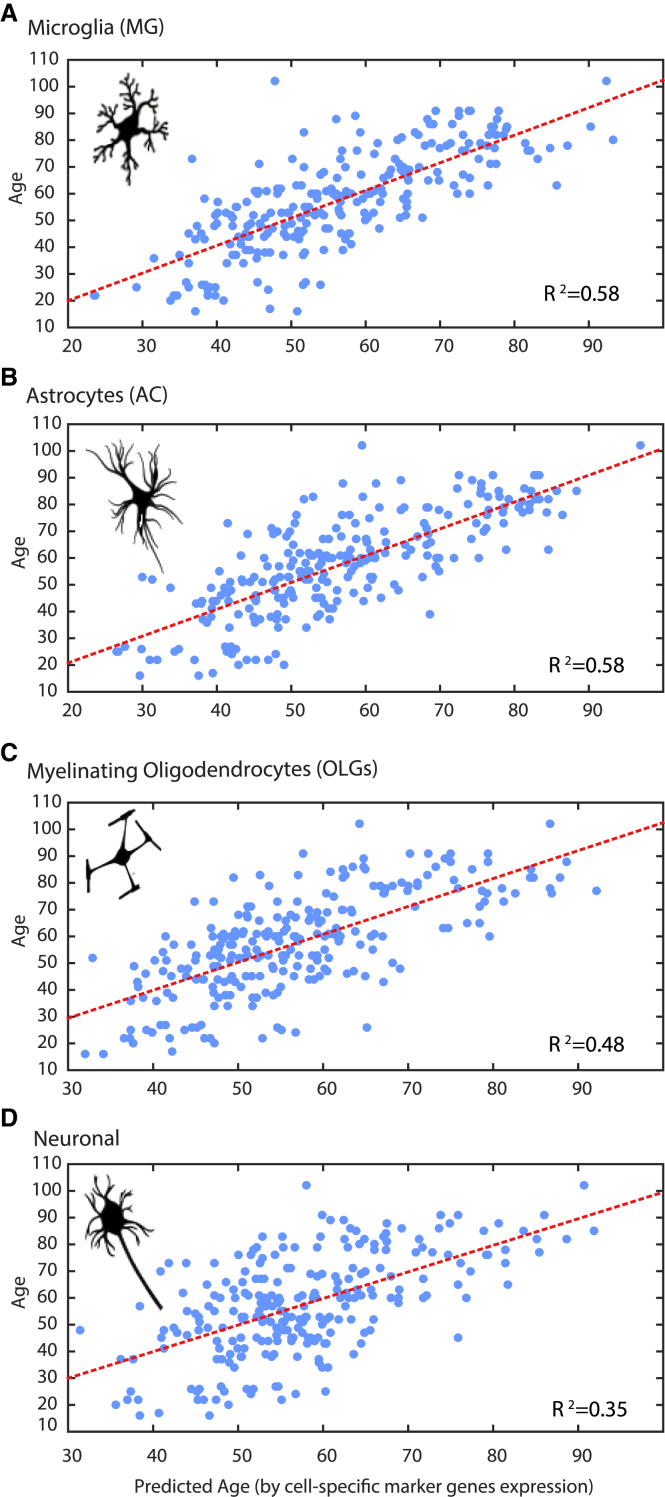
Glial-Specific Genes Are Most Capable of Predicting Biological Age (A–D) Analysis of the accuracy of cell-type-specific genes in predicting the biological age of UKBEC brain samples: (A) MG-specific genes (R^2^ = 0.58), (B) AC-specific genes (R^2^ = 0.58), (C) neuron-specific genes (R^2^ = 0.35), (D) OLG precursor-specific genes (R^2^ = 0.48). In all plots, the y axis denotes the actual age and the x axis denotes the predicted age. MG, microglia; AC, astrocytes; OLG, oligodendrocytes. See Figure S4C for age association plots of endothelial, OLG precursor, and newly formed OLG cell-specific genes.

Given our aforementioned findings, we next sought to gain insight into whether glial genes can predict age category with more fidelity than neurons and sought to understand the precise nature of gene expression changes driving this. Having established cross-regional and cell-type-specific gene expression relationships upon aging, we next asked which cell-type expression patterns within the brain are most associated with age. We applied a stepwise regression to construct an age-associative model based on the expression signals of cell-type-specific genes to compare them (Figure 7; Figure S4; Supplemental Experimental Procedures). We accounted for both the brain bank source and the cause of death. Application of the model uncovered defined groups of the highest age-relevant genes for each cell type. In a few cases, interaction between two genes was found to be age predictive (Table S3). Several multi-regional genes were also detected as age-predictive cell-type-specific genes, including *CP*, *SGPP1*, and *VWF*, which were detected as OLG or endothelial specific (Table S4). MG-, AC-, and endothelial-specific genes were most highly associated with biological age, while the smallest number of age-predictive genes was found among the neuron-specific genes (Figure 6). Altogether, our data implicate expression of glial-specific genes, rather than neuronal-specific genes, as the most reliable predictor of biological age in the human brain.

### Functional Enrichment Analysis of Multi-regional and Region-Specific Aging-Altered Genes

To gain further insight into the functional nature of aging-altered genes, we performed enrichment analysis of Gene Ontology (GO) terms of these genes (Edgar et al., 2013). The upregulated multi-regional genes were enriched in the following functional terms: “MG cell development”, “interleukin-1 (IL-1) receptor activity”, and “immune response”. Supporting these observations, neuroinflammation is known to be involved in aging, with evidence implicating the interferon type I response in aging-associated cognitive decline (Baruch et al., 2014). Conversely, downregulated multi-regional genes were enriched in the processes of “protein transport and localization”, and aging-altered expression of these genes led to shifts in regional identity (Figure S2B). Moreover, 244 of a total of 253 genes annotated to the “protein transport” category were detected as altered upon aging in at least one brain region. These genes separated the CRBL from the other regions and maintained their regional sub-classification (Figure S5Bi). In addition, the CRBL samples of the old group remained clearly separated from the rest (Figure S5Bii). Most aging-altered genes annotated to this category were downregulated in eight regions, apart from the CRBL and WHMT, which showed greater expression variability (Figure S5Biii). The WHMT aging-altered genes were functionally enriched in “regulation of cell adhesion”, “regulation of cell development”, “metabolic processes”, and “cognition” (Figure S6A). Conversely, among the top functional terms that were enriched in aging-altered genes in the FCTX were immune functions including “T cell differentiation”, “T cells”, and “leukocyte and lymphocyte activation” (Table S2). Among the CRBL-enriched functions were “cell adhesion”, “regulation of cell motion and migration”, and “neuron projection morphogenesis”. These results imply a region-specific functional heterogeneity of the brain aging process.

Analysis of the cell-type-specific aging-altered genes revealed enrichment of further functional pathways. “Synaptosome”, “regulation of programmed cell death”, and “metal ion transporter” were enriched in downregulated neuron-specific genes. “Regulation of adaptive immune response”, “natural killer cell-mediated cytotoxicity”, and “cell adhesion and motion” were enriched in MG-specific upregulated genes (Table S2). “Myelination”, “oxidoreductase”, and “RAS protein signal transduction” were enriched in upregulated OLG-specific genes. “Mitochondrial matrix”, “phosphate metabolic process”, and “Kyoto Encyclopedia of Genes and Genomes (KEGG) pathway AD” were enriched in downregulated myelinating OLG-specific genes. Finally, “cell morphogenesis” and “cell-cell adhesion” were enriched in upregulated AC-specific genes. Some of these functions have also been identified in a study that examined the initial cell-type-specific transcriptional changes in a mouse model of amyotrophic lateral sclerosis (ALS), including synaptic functions in neurons and membrane signaling defects in OLGs (Sun et al., 2015).

## Discussion

This study presents a comprehensive analysis of RNA expression in ten regions of the human brain and large-scale cell quantification in FCTX upon aging. Our findings show that cell-type-specific genes delineate samples based on both age group and brain region. Aging was the major determinant of glia-specific gene expression shifts in regional identity, while such changes were not evident in neuron-specific genes. Genes specific for neurons and OLGs generally decreased their expression upon aging, while MG-specific genes increased their expression profiles, consistent with the known MG activation in aging (Norden and Godbout, 2013). ACs showed a more complex pattern of reciprocal regional changes upon aging, with upregulation in the cortical regions and downregulation in the deeper brain structures. Among the genes specific for the non-neuronal cell types, those with the highest absolute expression in the young group decrease their expression upon aging in most brain regions, while those with the lowest expression in the young group increase their expression in a subset of regions. This leads to major shifts in region-specific gene expression, particularly of AC- and OLG-specific genes, which are most pronounced in the HIPP and SNIG, the regions that are archetypally affected in the most common age-related neurodegenerative diseases (AD and PD, respectively). These findings reinforce a growing body of evidence implicating glia in aging (Norden and Godbout, 2013).

Age-related degeneration of OLG has been previously observed in the HIPP of the senescence-accelerated mice, as well as other animal models (Hayakawa et al., 2007, Hwang et al., 2006, Shimeda et al., 2005). OLG-specific genes were also found to have the strongest enrichment among genes with decreased age-related expression in human TCTX (Tollervey et al., 2011). We demonstrate that the age-related downregulation of OLG-specific gene expression is accompanied by a decrease in OLG cell numbers in the FCTX, consistent with previous observations of decreased OLGs in neocortical regions of old human brains (Fabricius et al., 2013, Pelvig et al., 2008). The OLG-specific aging-altered genes include *MBP*, a major constituent of the myelin sheath, and *LINGO1*, a regulator of myelination (Mi et al., 2005). It is of interest that the low-density tiles (corresponding to likely white matter) show the largest decrease in oligodendrocytes and a corresponding increase in other cells (Figure 5). Given the strongest upregulation of MG-specific genes in the brain, it appears possible that the increase of other cells is driven by the increase in MG, but this remains to be directly examined.

We found increased AC-specific gene expression in human aging HIPP, which agrees with data from aging mouse models, in which increased proliferation and activation of ACs are reported (Hayakawa et al., 2007) (Figure 4). An examination of three sub-regions of mouse HIPP using three AC-specific protein markers revealed complex, region-specific, and marker-dependent changes (Rodríguez et al., 2014). Regionally encoded AC expression is important for neuronal functions, as was demonstrated by the loss of ventral spinal cord AC-encoded *SEMA3A* gene expression, which leads to selective death of α-motor neurons in mice (Molofsky et al., 2014). We find many regional differences in expression of AC-specific genes are largely erased in samples from the old group; for example, these genes cluster the HIPP and PUTM separately in the young group, but not in the old group (Figure 4B). This suggests that major changes in functional heterogeneity of AC take place in the aging brain, which might have deleterious consequences on the integrity of neuronal circuits.

A trend toward increased expression of MG-specific genes was observed in all regions upon aging, with corresponding upregulation of genes with immune or inflammatory functions. The upregulated genes include *C1Q*, which agrees with the increased C1Q protein levels that were observed in both mouse and human brains upon aging (Stephan et al., 2013). Another upregulated gene is *TREM2*, which is also upregulated in amyloid-plaque-associated MG (Frank et al., 2008) and contains variant alleles that increase AD risk (Guerreiro et al., 2013). Upregulation of inflammatory functions is in line with evidence implicating the interferon type I response in age-associated cognitive decline (Baruch et al., 2014).

In addition to glial changes, we also observed a decreased number of neurons with large cell bodies, which represent approximately 20% of neurons in the cortex. Although we did not attempt to directly identify the neuronal subtypes in the present study, neurons with the largest cell bodies are likely to be associative pyramidal neurons (Zeba et al., 2008). Furthermore, these neurons were previously indicated to be most vulnerable to aging in a study of Rhesus monkeys (Gilman et al., 2016). While our analysis indicates that the decrease in these pyramidal neurons may be the primary source of the downregulation of neuron-specific genes, our findings regarding the cortical neuronal cells remain speculative due to the limited number of individuals used for the imaging analyses. Moreover, it remains possible that the change does not result from loss of these neurons, but rather from downregulation of Rbfox3 protein, or its loss from the cytoplasm of large neurons. Thus, our current analysis will need to be verified with the use of additional markers of specific neuronal cell types and increased sample size, which will potentially include additional brain regions; ideally, it will also be compared to the outcomes of cell-type-specific analyses of RNA sequencing datasets (Lake et al., 2016).

Age is the major risk factor for both AD and PD, the two most prevalent neurodegenerative diseases. It is becoming clear that the pre-clinical stage of AD begins decades before clinical manifestation (Dubois et al., 2014). This pre-clinical stage has been termed “the cellular phase,” because it involves changes in interactions among all cell types in the brain, with the most dramatic changes taking place in AC, MG, and vasculature (De Strooper and Karran, 2016). We find a corrosion of glial region-specific gene expression in aging, with the genes specific for AC, MG, and endothelial cells being the best predictors of age. HIPP and SNIG are affected in the early stages of AD and PD, respectively, and these are the two regions with major shifts in their regional expression profiles of AC- and OLG-specific genes upon aging. Thus, our data may provide insights into the role of glia in the region-specific vulnerability in these age-related neurodegenerative diseases.

By simultaneously assessing changes in cell-type-specific genes across multiple brain areas, our study takes a step toward providing a comprehensive framework of the molecular and cellular changes in human aging. While our primary aim was to deconvolute the cell-type-specific signatures present within large databases of age-related transcriptional changes, we also made a step toward interpreting these in light of changes in counts of OLG and neuronal cells. Integration of further genome-wide and single-cell data from human tissues samples and cell and animal models will be required to fully understand the cellular and molecular mechanisms underlying the observations in our study. Altogether, our study indicates that the cellular changes during aging involve a dramatic shift in the regional identity of glia, and it provides a resource for further studies of the relationship between aging and the cellular phase of dementia.

### Conclusions

Our study examines brain-wide gene expression patterns in the aging human brain across a wide physiological age range, coupled with complementary analysis of cell-type-specific marker genes and validation by direct cell quantification using immunohistochemical imaging followed by targeted computational analysis. In addition to the expected increase in expression of MG-specific genes and decrease in expression of neuron-specific genes, our analyses uncovered major changes in the region-specific expression of AC- and OLG-specific genes. The age-associated changes in the regional expression of glial-specific genes are most dramatic in HIPP and SNIG, the brain regions affected in AD and PD. The age-dependent decrease in expression of OLG- and neuron-specific genes aligns with the results of direct cortical cell counting, in which decreased numbers of OLGs and of neurons with large cell bodies are demonstrated. We believe that our data and computational approaches provide a powerful resource for further study of the cellular and molecular changes taking place during human brain aging and provide insights into the pre-clinical cellular phase of dementia.

## Experimental Procedures

### Ethical Statement

All samples used for this study had fully informed consent for retrieval and were authorized for ethically approved scientific investigation (Research Ethics Committee number 10/H0716/3).

### Brain Samples

Post-mortem human brain material was produced under institutional guidelines governed by approved protocols. Tissue samples were produced from 99 individuals by the Sudden Death MRC brain bank, 35 individuals by the Sun Head Institute for the UKBEC, and 305 individuals by the American Brain Bank (NABEC).

### Quality Assessment and Array Pre-processing

For the UKBEC dataset, all quality measurements were extensively described in a previous publication (Trabzuni et al., 2011). The initial pre-processing of the microarray data, including application of RMA (robust multi-assay) average quantile normalization with guanine cytosine (GC) background corrections (GC-RMA) and expression data were log2 transformed. The gene level signal estimates were calculated for a total of 26,493 transcripts using the median signal of each group of probe sets interrogating a transcript.

### Expression Data Analysis

A tailored analysis pipeline was developed for all computational analyses and data visualization of microarray and RNA-seq datasets that were analyzed in this study (in MATLAB, R2014-2016a). Those include construction of data structures and statistical significance inference using ANOVA, with false discovery rate (FDR) thresholding (of corrected p < 1e−3), classification and clustering (e.g., using t-distributed stochastic neighbor embedding [t-SNE] and hierarchical clustering), and data visualization.

Cell-type-specific genes were defined by analysis of RNA-seq data from mouse brain (http://web.stanford.edu/group/barres_lab/brain_rnaseq.html) and were further used to find age-predictive cell-specific genes. The lists are under Tables S5 and S6, accordingly. Additional cell-specific lists were based on a previous microarray data on three of these cell types (Cahoy et al., 2008). Further details are in the Supplemental Information.

### High-Resolution Imaging and Analysis of Immunolabeled Brain Samples

Post-mortem human brain sections were placed into xylene and rehydrated. Antigen retrieval was performed with citric acid. For OLG staining, the samples were immunolabeled with OLIG2 antibody using the Leica Novolink Polymer detection kit. We used the Olig2 antibody from Millipore (catalog #AB9610) at 1/200 dilution. For staining of neurons, the samples were immunolabeled with NeuN antibody (Acris) and the Leica Bond Epitope Retrieval Solution#1 was used (AR9961 from Leica Biosystems) (AR9961 from Leica Biosystems). The images for both types of stains were acquired on the Zeiss AxioScan slide scanner. Details of the cell detection and quantification computational methods for neurons are given under the Supplemental Experimental Procedures. In addition, all raw jpeg images of the slides can be seen at https://figshare.com/s/f2675361af1242f3565f. For image analysis, we employed some of the computational methods mentioned by Bjornsson et al. (2008), in addition to a targeted computational pipeline developed in-house in MATLAB (see details under Supplemental Experimental Procedures).

## Consortia

The members of the UK Brain Expression Consortium are John Hardy, Mina Ryten, Daniah Trabzuni, Sebastian Guelfi, Michael E. Weale, Adaikalavan Ramasamy, Paola Forabosco, Colin Smith, and Robert Walker. The members of the North American Brain Expression Consortium are Sampath Arepalli, Mark R. Cookson, Allissa Dillman, J. Raphael Gibbs, Dena G. Hernandez, Michael A. Nalls, Andrew B. Singleton, Bryan Traynor, Marcel van der Brug, Luigi Ferrucci, Robert Johsnon, Ronal Zielke, Dan L. Longo, Juan Toncoso, and Alan Zonderman.

## Author Contributions

L.S., R.P., and J.U. conceived and designed the project and wrote the manuscript with contributions from all co-authors; J.U. and R.P. contributed equally to the work. L.S. developed the computational pipelines, analyzed the data, and produced the figures. E.S. developed targeted imaging computational analyses. M.R. and D.T. provided RNA extraction and microarray sample preparations. J.H. and M.R. provided access to the UKBEC dataset, and M.R.C. provided access to the NABEC datasets. J.R. and C.S. provided immunohistochemistry preparation, and R.P. supervised the immunohistochemical slide analysis and interpretation.

## Accession Numbers

The accession numbers for the UKBEC exon and NABEC expression datasets reported in this paper are GEO: GSE46706 and GSE36192, respectively.

## Supplemental Information

Document S1. Supplemental Experimental Procedures, Figures S1–S7, and Table S4

Table S1. UKBEC Cohort Samples Details, Related to Figure 1

Table S2. Functional Enrichment of Brain Aging-Altered Genes, Related to Figure 2

Table S3. Cell-Type Marker Genes Altered in the Aging Brain, Related to Figure 4

Table S5. Age-Associated Cell-Specific Genes, Related to Figures 4 and 7

Table S6. Age-Associated Cell-Specific Genes, Related to Figure 4

Table S7. Aging-Altered Genes that Were Also Found as Methylated upon Aging (Based on Comparison to Other Datasets), Related to Figures 4 and S7

Document S2. Article plus Supplemental Information
