# Supplementary material for: Major Shifts in Glial Regional Identity Are a Transcriptional Hallmark of Human Brain Aging
Source: Cell Rep. 2017 Jan 10;18(2):557–70. doi: 10.1016/j.celrep.2016.12.011 (PMC5263238; doi:10.1016/j.celrep.2016.12.011)
Supplement: Document S1. Supplemental Experimental Procedures, Figures S1–S7, and Table S4 [file mmc1.pdf]

Cell Reports, Volume *18*

Supplemental Information

Major Shifts in Glial Regional Identity Are a Transcriptional Hallmark of Human Brain Aging

Lilach Soreq, UK Brain Expression Consortium, North American Brain Expression Consortium,, Jamie Rose, Eyal Soreq, John Hardy, Daniah Trabzuni, Mark R. Cookson, Colin Smith, Mina Ryten, Rickie Patani, and Jernej Ule

Table of Contents[1]

1. SUPPLEMENTARY EXPERIMENTAL PROCEDURES 3

STUDY DATA SETS 3

RNA EXPRESSION DATA SETS 3

THE UKBEC DATA SET 3

THE NABEC DATA SET 4

REST FCTX DATA SET 4

RNA ISOLATION 4

UK-BEC EXON ARRAYS: RNA EXTRACTION IS DESCRIBED UNDER TRABZUNI ET AL. (TRABZUNI ET AL., 2011). 5

NABEC 3' ARRAYS: THE RNA ISOLATION AND ARRAY PROCESSING AS WELL AS QUALITY MEASUREMENTS ARE REPORTED IN RAMASAMY ET AL. (E.G (RAMASAMY ET AL., 2013A), (HERNANDEZ ET AL., 2012)). 5

HIGH-RESOLUTION IMMUNOHISTOCHEMICAL BRAIN IMAGING DATASET PRODUCTION 5

IMAGE ACQUISITION OF THE HIGH-RESOLUTION FCTX OLIG2 STAINED IMAGES 6

NEUN STAINED SECTIONS IMAGE ACQUISITION 7

ANALYSIS METHODS 8

MICROARRAYS 8

CELL TYPE SPECIFIC GENES: DEFINITION AND CLASSIFICATION ANALYSES 9

3. HIGH RESOLUTION SCANNED IMMUNOHISTOCHEMICAL STAINED IMAGES COMPUTATIONAL QUANTIFICATION 10

OLIG2 STAINED BRAIN SECTIONS IMAGE ANALYSIS 10

CELL QUANTIFICATION AND CLASSIFICATION MODEL 10

PERMUTATION TEST ON THE OLIG2 STAINED SLIDES 11

PERMUTATION TESTS ON THE NEUN STAINED SLIDES 13

4. CELL TYPE MARKER GENES AND AGE-ASSOCIATION STATISTICAL ANALYSIS 13

[1] Soreq L. et al., Major shifts in glial regional identity are a transcriptional hallmark of human brain aging

5. SUPPLEMENTARY TABLES LIST AND LEGENDS 13

SUPPLEMENTARY TABLE 1| UKBEC COHORT SAMPLES DETAILS, RELATED TO FIGURE 1 13

SUPPLEMENTARY TABLE 2| FUNCTIONAL ENRICHMENT OF BRAIN AGING ALTERED GENES, RELATED TO FIGURE 2 14

SUPPLEMENTARY TABLE 3| CELL TYPE MARKER GENES ALTERED IN THE AGING BRAIN, RELATED TO FIGURE 4 14

SUPPLEMENTARY TABLE 4| CROSS REGIONAL HUMAN AGING ALTERED GENES, RELATED TO FIGURE 3 15

SUPPLEMENTARY TABLE 5| AGE ASSOCIATED CELL SPECIFIC GENES, RELATED TO FIGURES 4 AND 7 15

SUPPLEMENTARY TABLE 6| AGE ASSOCIATED CELL SPECIFIC GENES, RELATED TO FIGURE 4 16

SUPPLEMENTARY TABLE 7| AGING ALTERED GENES THAT WERE ALSO FOUND AS METHYLATED UPON AGING (BASED ON COMPARISON TO OTHER DATASETS), RELATED TO FIGURES 4 AND S7 16

8. SUPPLEMENTAL REFERENCES 17

1. Supplementary experimental procedures

Study data sets

RNA expression data sets

The UKBEC data set

Brain tissues originated from 134 Caucasian European individuals (with two exceptions, 1 of Mexican ancestry and 1 of Chinese ancestry, see further details under Table S1). Of these, 101 brains were obtained from the MRC sudden Death Brain and Tissue Bank, Edinburgh, UK (Millar et al., 2007) 33 of them originating from the Sun Health Research Institute (SHRI, USA), an affiliate of Sun Health Corporation, USA (Beach et al., 2008). None of the individuals had neuropathologically diagnosable conditions, or presented with neurological or neuropsychiatric conditions. A detailed description of the samples used in the study, tissue processing and dissection is provided in the in Trabzuni et al. (Beach, 2008; Trabzuni et al., 2013). All the samples had fully informed consent for retrieval and were authorized for ethically approved scientific investigation (National Hospital for Neurology and Neurosurgery and Institute of Neurology Research Ethics Committee, 10/H0716/3).

The brain samples were extracted post mortem from up to 10 brain regions per individual. The individuals aged from 16 (the youngest) to 102 years. For each individual, the samples were produced from up to 10 different anatomical brain regions: Frontal cortex (FCTX) from Brodmann areas 8 and 9, Cerebellar cortex (CRBL), Hippocampus (HIPP), Substantia Nigra (SNIG), Putamen (PUTM), Thalamus (THAL), Medulla (specifically, the inferior olivary nucleus) (MEDU), Temporal Cortex (TCTX) and the Occipital cortex (OCTX) from Brodmann area 17 and from intralobular white matter (WHMT, which was separated from the grey matter). In terms of aging altered genes, we detected genes that were detected as

differentially expressed in different brain regions, subsets of brain regions or in a brain-wide fashion and aging altered cell specific genes.

The NABEC data set

To further increase the number of samples and add an external, independent dataset for computational validation purposes, we examined a large additional independent dataset of 607 post mortem brain samples originating from 305 control individuals (N = 101 females and 204 males) between the ages 16 to 101 that had no neuropathological diagnosis nor neurological or neuropsychiatric conditions. Sub-dissected samples from cerebellar and frontal cortex brain regions were frozen before processing (Ramasamy et al., 2013a). The dataset included an overall of 607 Illumina HT12 v3 BeadChip microarray raw CEL files, which interrogated RNA from 305 post mortem FCTX brain samples, and 302 CRBL samples from the same individuals.

REST FCTX data set

RNA extraction from 39 human cortical samples, as well as microarray hybridization protocols were previously described under (Loerch et al., 2008), the minimal age was 24 and the maximal - 106. The microarray platform used is Affymetrix Human Genome U133plus 2.0 Arrays. Postmortem human cortical samples were derived from subjects that did not carry a diagnosis of Alzheimer's disease or another neurodegenerative disease, and showed neuropathological findings within the normal range for age (Loerch et al., 2008). Overall, we analyzed up to 10 brain regions from 480 individuals aged from 16 to 106 and over 1800 microarray samples.

RNA Isolation

UK-BEC exon arrays: RNA Extraction is described under Trabzuni et al. (Trabzuni et al., 2011).

NABEC 3' arrays: the RNA isolation and array processing as well as quality measurements are reported in Ramasamy et al. (e.g (Ramasamy et al., 2013a), (Hernandez et al., 2012)).

High-resolution immunohistochemical brain imaging dataset production

To complement out RNA expression findings, we have generated high resolution imaging data for 6 selected BA9 (FCTX) samples using OLIG2 antibody (to stain oligodendrocytes (OLGs), where the DAB+ nuclear product is seen brown) and with Heamotoxylin as a counterstain (the nuclei of other cell types are stained in blue). Briefly, the brain sections were placed into 2 changes of Xylene to remove wax – 3 mins each, with varying degrees of alcohol (70%, 99% and absolute alcohol) to rehydrate – 3 mins each. The samples were then placed in picric acid to remove any artifacts – ca15 minutes. Subsequently, the samples were placed under running water to remove all picric residues (ca15 mins). Finally, antigen retrieval was performed, 1:200 with Citric Acid retrieval (5% Citric Buffer pH 6.0 heated to 125°C for 30 seconds then allowed to cool).

The remaining process was done via the Leica Novolink Polymer Detection Kit:

1. Peroxidase Block – 30 mins
2. Tris Buffered Saline – 5 mins
3. Protein Block – 15 mins
4. Tris Buffered Saline – 5 mins
5. Primary Antibody – 30 mins
6. Tris Buffered Saline – 5 mins
7. Post Primary Block – 30 mins
8. Tris Buffered Saline – 5 mins

9. Novolink Polymer – 30 mins
10. Tris Buffered Saline – 5 mins
11. DAB secondary (50ul chromogen/1ml substrate buffer)
12. Counterstained with Heamotoxylin – 30 seconds

Slides then dehydrated, through to Xylene and mounted.

The images were acquired on the 'Zeiss AxioScan Slide Scanner.

Details and specs of the system can be found here:

www.zeiss.co.uk/microscopy/en_gb/products/imaging-systems/axio-scan-z1.html

Magnification of the scans done set to x20 and in the Brightfield setting.

NeuN slides staining

In the NeuN staining protocol, antibody used was from Acris (code AM10122SU-N) in dilution of 1:200. NeuN antibody was Acris AM10122SU-N was used at 1:200 dilution with citrate buffer pre-treatment for antigen retrieval. The retrieval method was Leica Bond ERI solution for 20 minutes (citric acid @6pH). All pre-treatments including dewaxing and counterstains (haematoxylin) were carried out on the Leica Bond III IHC staining machine. The staining protocol used is Protocol F (factory set one).

Image acquisition of the high-resolution FCTX OLIG2 stained images

Post-mortem human brain sections were placed into 2 changes of Xylene to remove wax (3 minutes each). Varying degrees of alcohol (70%,99% and absolute) used to rehydrate (3 minutes each), placed in Picric Acid to remove any artifacts and were put under running water to remove all picric residue (ca 15 minutes). Antigen retrieval was performed subsequently, 1:200 with Citric acid for the OLIG2 staining (citric acid: 5% Citric Buffer pH 6.0 heated to 125oC for 30 seconds then allowed to cool). The Leica Novolink Polymer detection kit was then applied as follows: Peroxidase Block - 30 minutes, Tris buffered Saline – 5 minutes, Protein block – 15 minutes, Tris buffered Saline – 5 minutes, Primary

antibody – 30 minutes, Tris Buffered Saline – 5 minutes, Post primary block – 30 minutes, Tris buffered Saline – 5 minutes, Novolimk Polymer – 40 minutes, Tris buffered Saline – 5 minutes and DAB secondary (50ul chromogen/1ml substrate buffer), Counterstained with Heamotoxylin – 30 seconds and slides were then dehydrated, through to Xylene and mounted. The images were acquired on the 'Zeiss AxioScan Slide Scanner. Details and specs of the system can be found here: www.zeiss.co.uk/microscopy/en_gb/products/imaging-systems/axio-scan-z1.html. Magnification of the scans done set to x20 and in the Brightfield setting.

NeuN stained sections image acquisition

Image acquisition from the FCTX NeuN stained sections was done similarly to the OLIG2 stained samples, the images were acquired on the 'Zeiss AxioScan Slide Scanner. In order to account for the large diversity of cell size and shape, in the quantification of neuronal cells based on the NeuN stained slides, we increased tile size from the native device tile 1600 x 1200 to a larger tile of 10k x10k pixels. This was followed assessing the entropy of each slide, a statistical measurement that captures the randomness across grayscale image using the following formula $E = -\sum p_i log(p_i)$ and excluding any image below entropy of 5. Then we preform element wise multiplication across the inverse red and blue image channel to create the 16bit interaction image that contains both NeuN staining as well as the supporting cells all transformed from local minima to local maxima – converting "valleys to hills". This is followed by estimating five multilevel thresholds using Otsu method (Otsu, 1975). And creating a segmented binary image using the maximum threshold. Morphological operations are followed to ensure segmentation integrity and areas smaller then a lower bound of 500 pixels is enforced.

This is followed by applying the watershed transform (Meyer, 1994) to separate joined segments. And finished by harvesting additional per cell statistics. Visual plots are produced per tile to enable

visual inspection of the quality of cell detection. The raw .jpeg images of the NeuN stained slides can be seen under FigShare portal (link: https://figshare.com/s/f2675361af1242f3565f).

ANALYSIS METHODS

Microarrays

To analyze these extensive datasets, we developed dedicated programmed computer programs. Specifically, we have implemented a number of functions and scripts for mathematical, computational and statistical analyses of the microarrays and RNASeq data, including (1) definition of age groups based on histogram bins (16-44 years old, 45-79 years old and 80 – 106 years old), (2) microarray processing including identification of gene differentially expressed upon aging in each brain region, (3) comparisons between different brain regions, (4) expression correlations, (5) classification (6) enrichment analysis of the RNASeq data from specific cell types, (7) cellular specificity of the aging altered genes and (8) data visualization. These tailored mathematical and statistical analyses were conducted through tailored Matlab (version R2013B) programs that also called functions from Matlab Statistics toolbox. First, we divided the UKBEC microarray samples to three age groups (young, middle and old) by a mathematical calculation, using histogram bins of all the ages that were included in the dataset. Subsequently, significance p-values were computed using ANOVA (and FDR correction, $q < 1e-3$) based on the gene level expression signals of the interrogated genes. Subsequently, partial least squares regression (PLSR) linear regression model was further used to rank the aging altered genes based on the age prediction accuracy of these genes. We then applied unsupervised clustering and classification methods on the expression data, to investigate grouping of the samples of different age groups and brain regions based on expression signals of different groups of genes. Those methods included hierarchical classification (HCL), which was applied using the Euclidean distance and average linkage method. We also applied a powerful non-linear parametric dimensionality reduction technique called t-Distributed Stochastic Neighbor Embedding (t-SNE) (Laurens van der Maaten, 2008) to create a 2D visualization of local and global data structures based on different groups of genes identified as altered in the aging brains including

cell type top markers. The technique is a variation of the Stochastic Neighbor Embedding method (termed SNE, Hinton and Roweis, 2002), with the aim to preserve as much of the significant structure of the high dimensional data as possible in a low dimensional map. SNE minimizes the sum of Kullback-Leiber divergences over all data points using a gradient descent method. The variance of the Gaussian noise is reduced through iterative process. We applied support vector machine (SVM) to classify the samples to age groups based on the expression patterns of the genes commonly altered in all the brain regions upon aging. Functional enrichment analysis to detect enriched functional groups of genes was conducted through the DAVID (Dennis et al., 2003) resource EASE (Hosack et al., 2003) program on the lists of genes found as altered in different brain regions upon aging, as well as to functionally analyze cell specific aging genes. To measure relationships among the annotation terms based on the degrees of their co-association genes the similar, redundant, and heterogeneous annotation contents from the same or different resources were grouped into annotation groups. A modified Fisher Exact P-value calculated for gene-enrichment analysis. $P < 0.05$ considered as significant. The number of differentially expressed genes per term was compared against the human genome background.

Cell type specific genes: definition and classification analyses

Cell-type specific genes were defined by analysis of RNASeq data from mouse brain (http://web.stanford.edu/group/barres_lab/brain_rnaseq.html) through calculation of enrichment score based on the normalized RNASeq read counts from the mice cortex RNA libraries. We calculated the enrichment p-value for the 7 cell types (which included neurons and glial types), per each separately. The full list of markers is given under Table S5. We further used the defined lists of genes expression profiles to find age predictive genes (Table S6). Additional cell specific lists were based on a previous microarray data on four cell types (Cahoy et al., 2008). Additionally, we used expression data from 24 central nervous system cell types interrogated by microarrays was to classify the brain samples

(http://web.stanford.edu/group/barres_lab/brainseqMariko/brainseq2.html), Table S7. Further details are under supporting material.

3. High resolution scanned immunohistochemical stained images computational quantification

OLIG2 stained brain sections image analysis

To analyze slides produced by high throughput imaging on brain sections from selected old and young individuals (N=3 of each age group), a tailored systematic computational pipeline was developed using Matlab (R2015a). The pipeline preforms a cell counting procedure for each independent tile, followed by statistical permutation assessment. The quantification module includes the following steps: 1. For slide in each sample (typically composed of several thousands of individual multi-channel images here called slides): For each slide, entropy (i.e. $H = -\sum_{i=1}^{M} p_k \log_2(p_k)$) was calculated and the exclusion criteria of H=5 was used to discard slides without useful information. 2) Morphological top-hat and bottom-hat filtering using a disk kernel was performed to account to local noise. 3) A pyramidal Gaussian mixture model (k=4) was performed on the slide to label each pixel and using a majority vote across pyramidal levels a binary image was produced. 4) Morphological procedures were used to discard border elements as well as hole filling, and lower and upper area thresholds. 5) Watershed algorithm was performed to separate overlapping cells. 6) Each estimated cell was classified using an expertly trained classification model (see below) to further eliminate non-cell objects.

Cell quantification and classification model

For analysis of OLG, the tiles of each OLIG2 stained image were extracted. Images with low entropy were discarded (<5) and the information based filtering was followed by background and noise filtering.

Then the lower threshold was uncovered using empirical Pareto distribution modelling, and thresholding of the three color channels using this measurement to create a binarized segmentation of the image. This was followed by circle detection using circular Hough transform. Lastly, each circle was assigned a class using a previously trained random forest model based on manual expert classification of 2000 events (i.e stained cells) containing both brown- and blue- stained noise ones. For each slide, the output included a summary of the number of cells counted as true in it, per each of four density groups representing four different cell densities. The difference between age groups was calculated using permutation tests on the average number of cells per each density class. For training of the machine-learning algorithm, about 2800 individual cells were manually labeled as 'blue', 'brown' (the OLG stained cells through the DAB+ nuclear product), or 'other' (which represents technical artifacts/noise). This data was used to train a Random Forest learning algorithm and to generate a classification model.

For analysis of neurons, we accounted for the large diversity of cell size and shapes by increasing the tile size from the native device tile. We assessed the entropy of each slide, and assessed statistically the entropy of each slide for filtering purposes, preformed element wise multiplication across the inverse red and blue image channel, estimated five multilevel thresholds and application of watershed transform. Area statistics per slide is aggregated into one large database, and data is binned into four size groups. For each bin, 100 random iterations over 1000 permutation analysis are performed to assess the empirical p-value over the null T-distribution. In order to account for the large diversity of cell size and shape, in the quantification of neuronal cells based on the NeuN stained slides, we increased tile size from the native device tile. Briefly, assessed the entropy of each slide, and assessed statistically the entropy of each slide for filtering purposes, preformed element wise multiplication across the inverse red and blue image channel, estimated five multilevel thresholds and application of watershed transform. Further details about the computational methods for quantification of neurons are given under the supporting methods.

Permutation test on the OLIG2 stained slides

SUPPLEMENTAL INFORMATION

Each image is constructed from thousands of tiled images each approximately 0.6mm wide and 0.46mm tall. For each tile, the number of cells was extracted. Statistical significance was calculated on groups for both types of stained cells on overall 10,922 aging and 8,766 young stained tiles. To account for the large imbalance in the total number of slides we randomly selected an equal number of 100 tiles per sample, and repeated this process across 100 iterations and 1000 random shuffling permutations. To further examine the effects of the two different cellular populations in BA9 (likely corresponding to gray matter cortical areas vs. the least dense, WHMT) we classified the different tiles based on the total cell density (regardless of sample) to four different groups based on density (low, low-med, med-high and high which generally may be attributed to white matter vs. grey matter areas). The significance p-value was calculated using normal distribution cumulative distribution function (CDF) per permutation using the following formula $T_n = \frac{T_0 - \bar{T}}{std(T)}$ where T_0 corresponds to the true labels.

Overall 6 BA9 (FCTX) samples sections stained with OLIG2 were analyzed (3 young and 3 old samples). In total, 49,822 stained FCTX (BA9) image tiles were captured for all samples (number of image tiles in the old brains = 24,061, and in young ones = 25,761). Overall 20,190 tiles survived signal to noise ratio (SNR) exclusion criteria (old = 8,992, young = 11,198). Generally, the cell detection and quantification pipeline included the following steps: SNR exclusion criteria, image enhancement, followed by object detection of all the cells in each slide This process was performed using a non-linear machine-learning model that was created using a large dictionary of positive and negative examples annotated manually by experts (general computational flow under Figure 5B). For each slide, the number of cells as well as morphological features per cell (including area, perimeter and roundness) was extracted. Overall, 393,625 cells (old = 172,868, young = 220,757) were quantified. Of these, 183,898 were brown (OLG precursor) cells (old = 76,926, young =106,972) and 209,727 were blue (old = 95,942, young =113,785). In the high-density slides we randomly selected 50 slides from each sample and run a

similar permutation test to the one above (old = 1828, young = 2612). FDR correction over the p-values was performed to account to the multiple iterations. The tiles were classified to 4 different density groups (low, low-med, low-high and high) using k-means. Further details are under supporting methods.

Permutation tests on the NeuN stained slides

Per-slide Area statistics are aggregated into one large database, and data is binned into four size groups. For each bin 100 random iterations over 1000 permutation analysis are performed to assess the empirical p over the null t distribution.

4. Cell type marker genes and age-association statistical analysis

The cell type gene markers for seven cell types were defined based on statistical analysis of available RNA-Sequencing data produced by the Barres lab (http://web.stanford.edu/group/barres_lab/brain_rnaseq.html), Table S3. For each group of cell type markers, an age predictive model was generated. Briefly, the UKBEC cohort samples were partitioned into training (60%), testing (20%) and validation (20%) sets, and gender, brain bank source and cause of death were incorporated as covariates. Following variable elimination using Competitive adaptive reweighted sampling (CARS), the model was assessed and stepwise fit was calculated.

5. Supplementary Tables list and legends

Supplementary Table 1| UKBEC cohort samples details, related to Figure 1

The details for the UKBEC samples and microarray sample numbers are given for each of the 1,231 analyzed brain samples. The details include: brain bank, individual ID, central nervous system (CNS) region, RNA Integrity number (RIN), chronological age, Post Mortem Interval (PMI) - in hours, brain tissue pH, cause of death code, gender and ethnicity. Reference in the main paper text: page 5, line 8.

Supplementary Table 2| Functional enrichment of brain aging altered genes, related to Figure 2

Results of functional enrichment analysis based on the genes that were detected as altered in each of the analyzed 10 brain regions upon aging, and of the genes commonly altered in 8-10 brain regions upon aging. For each brain region, the top 200 genes were functionally analyzed. The functional analysis was conducted using the DAVID resource EASE tool (Hosack et al., 2003), and with Gene Analytics LifeMap tool (Edgar et al., 2013). The functional analysis was conducted for each brain region, on the top 200 aging altered genes (scores were calculated using a PLSR model) for each of the 10 brain regions. Reference in the main paper text: page 14, line 22.

Supplementary Table 3| Cell type marker genes altered in the aging brain, related to Figure 4

Genes found as top enriched brain cell markers based on t-test of average RPKM values of RNASeq mouse transcriptome data of glia, neurons and vascular cells of rats cerebral cortex for 7 cell types: endothelial cells, neurons, oligodendrocyte (OLG) precursors, newly formed OLGs, myelinating OLGs, microglia and astrocytes (Zhang et al., 2014a), that were also found as altered in the human brain upon aging. Specific cell type marker genes that were also altered in the brain upon aging, including cell type

specific enrichment score and p value. The specific cell type marker genes were identified by statistical analysis of the RNASeq data produced by the Barres laboratory from seven rat brain cell types. The Barres lab RNA brain RNASeq data resource can be found under: http://web.stanford.edu/group/barres_lab/brain_rnaseq.html). Normalized RNASeq counts were analysed (Zhang et al., 2014b)).

To complement our cell specific findings, we also analyzed the recent Barres brain RNASeq transcriptome dataset (published in Neuron, 2016, This data was produced from human fetal brains, and includes neurons, microglia, astrocytes and endothelial cells, and from several brain regions (temporal cortex, white matter, parietal cortex, and hippocampus). We used the normalized FPKM data for our analyses. This recent cell specific data is available under: web.stanford.edu/group/barres_lab/brainseqMariko/brainseq2.html.

Additionally, we used the microarray data of 24 Central Nervous System (CNS) populations, of the resource that was published by Doyle et al. (Doyle et al., 2008)to define additional lists of cell type specific genes and investigate their expression patterns upon aging using our dataset. Reference in the main paper text: page 8, line 15.

Supplementary Table 4| Cross regional human aging altered genes, related to Figure 3

This table legend is given directly following the table, which is in this file. Reference to the table in the main paper text: page 13, line 21.

Supplementary Table 5| Age associated cell specific genes, related to Figures 4 and 7

Cell type specific markers that were found by statistical enrichment analysis of RNASeq data from rat brains (reference: Zhang, Y., et al., An RNA-sequencing transcriptome and splicing database of glia, neurons, and vascular cells of the cerebral cortex. J Neurosci, 2014. 34(36): p. 11929-47) (Zhang et al., 2014b), for each of the 7 analyzed cell type populations. For each gene, given are the gene symbol, intercept estimate, standard error, T statistic and p-value. Reference in the main paper text: page 20, line 17.

Supplementary Table 6| Age associated cell specific genes, Related to Figure 4

Cell type specific markers that were found as age predictive by a linear model, for each of the 7 analyzed cell type populations. For each gene, given are the gene symbol, intercept estimate, standard error, T statistic and p value. Reference in the main paper text: page 20, line 17.

Supplementary Table 7| Aging altered genes that were also found as methylated upon aging (based on comparison to other datasets), Related to Figures 4 and S7

Upon comparison to the aging methylation/expression study data (reference: (Horvath, 2013)), a large number of the reported aging methylated brain genes were also found in our study as differentially expressed upon aging. Additionally, genes that we detected as altered upon aging that were also found as top neuronal cell type markers (based on Doyle et al 24 cell type microarray study (reference: Application of a translational profiling approach for the comparative analysis of CNS cell types, Doyle J. P. et al., Cell 2008) are given as well. Reference in the main paper text: page 23, line 16.

6. EXPRESSION DATASETS ACCESSION CODES

UKBEC Gene Expression Omnibus (GEO): GSE36192

NABEC - see under (Ramasamy et al., 2013b) and GEO accession number: GSE36192.

8. SUPPLEMENTAL REFERENCES

Beach, T.G., Sue, L.I., Walker, D.G., Roher, A.E., Lue, L., Vedders, L., Connor, D.J., Sabbagh, M.N., and Rogers, J. (2008). The Sun Health Research Institute Brain Donation Program: description and experience, 1987-2007. Cell and tissue banking *9*, 229-245.

Beach, T.G.e.a. (2008). The Sun Health Research Institute Brain Donation Program: description and experience, 1987-2007. Cell Tissue Bank *9*, 229-245.

Cahoy, J.D., Emery, B., Kaushal, A., Foo, L.C., Zamanian, J.L., Christopherson, K.S., Xing, Y., Lubischer, J.L., Krieg, P.A., Krupenko, S.A.*, et al.* (2008). A transcriptome database for astrocytes, neurons, and oligodendrocytes: a new resource for understanding brain development and function. J Neurosci *28*, 264-278.

Dennis, G., Jr., Sherman, B.T., Hosack, D.A., Yang, J., Gao, W., Lane, H.C., and Lempicki, R.A. (2003). DAVID: Database for Annotation, Visualization, and Integrated Discovery. Genome biology *4*, P3.

Doyle, J.P., Dougherty, J.D., Heiman, M., Schmidt, E.F., Stevens, T.R., Ma, G., Bupp, S., Shrestha, P., Shah, R.D., Doughty, M.L.*, et al.* (2008). Application of a translational profiling approach for the comparative analysis of CNS cell types. Cell *135*, 749-762.

Edgar, R., Mazor, Y., Rinon, A., Blumenthal, J., Golan, Y., Buzhor, E., Livnat, I., Ben-Ari, S., Lieder, I., Shitrit, A.*, et al.* (2013). LifeMap Discovery: the embryonic development, stem cells, and regenerative medicine research portal. PLoS One *8*, e66629.

Hernandez, D.G., Nalls, M.A., Moore, M., Chong, S., Dillman, A., Trabzuni, D., Gibbs, J.R., Ryten, M., Arepalli, S., Weale, M.E.*, et al.* (2012). Integration of GWAS SNPs and tissue specific expression profiling reveal discrete eQTLs for human traits in blood and brain. Neurobiology of disease *47*, 20-28.

Horvath, S. (2013). DNA methylation age of human tissues and cell types. Genome Biol *14*, R115.

Hosack, D.A., Dennis, G., Jr., Sherman, B.T., Lane, H.C., and Lempicki, R.A. (2003). Identifying biological themes within lists of genes with EASE. Genome Biol *4*, R70.

Laurens van der Maaten, G.H. (2008). Visualizing Data using t-SNE Journal of Machine Learning Research *9*, 2579-2605

Loerch, P.M., Lu, T., Dakin, K.A., Vann, J.M., Isaacs, A., Geula, C., Wang, J., Pan, Y., Gabuzda, D.H., Li, C.*, et al.* (2008). Evolution of the aging brain transcriptome and synaptic regulation. PloS one *3*, e3329.

Meyer, F. (1994). Topographic distance and watershed lines. Signal processing *38*, 113--125.

Millar, T., Walker, R., Arango, J.C., Ironside, J.W., Harrison, D.J., MacIntyre, D.J., Blackwood, D., Smith, C., and Bell, J.E. (2007). Tissue and organ donation for research in forensic pathology: the MRC Sudden Death Brain and Tissue Bank. The Journal of pathology *213*, 369-375.

Otsu, N. (1975). A threshold selection method from gray-level histograms. Automatica *11*, 23-27.

Ramasamy, A., Trabzuni, D., Gibbs, J.R., Dillman, A., Hernandez, D.G., Arepalli, S., Walker, R., Smith, C., Ilori, G.P., Shabalin, A.A.*, et al.* (2013a). Resolving the polymorphism-in-probe problem is critical for correct interpretation of expression QTL studies. Nucleic Acids Res *41*, e88.

Ramasamy, A., Trabzuni, D., Gibbs, J.R., Dillman, A., Hernandez, D.G., Arepalli, S., Walker, R., Smith, C., Ilori, G.P., Shabalin, A.A.*, et al.* (2013b). Resolving the polymorphism-in-probe problem is critical for correct interpretation of expression QTL studies. Nucleic acids research *41*, e88.

Trabzuni, D., Ramasamy, A., Imran, S., Walker, R., Smith, C., Weale, M.E., Hardy, J., Ryten, M., and North American Brain Expression, C. (2013). Widespread sex differences in gene expression and splicing in the adult human brain. Nature communications *4*, 2771.

Trabzuni, D., Ryten, M., Walker, R., Smith, C., Imran, S., Ramasamy, A., Weale, M.E., and Hardy, J. (2011). Quality control parameters on a large dataset of regionally dissected human control brains for whole genome expression studies. J Neurochem *119*, 275-282.

Zhang, Y., Chen, K., Sloan, S.A., Bennett, M.L., Scholze, A.R., O'Keeffe, S., Phatnani, H.P., Guarnieri, P., Caneda, C., Ruderisch, N.*, et al.* (2014a). An RNA-sequencing transcriptome and splicing database of glia, neurons, and vascular cells of the cerebral cortex. J Neurosci *34*, 11929-11947.

Zhang, Y., Chen, K., Sloan, S.A., Bennett, M.L., Scholze, A.R., O'Keeffe, S., Phatnani, H.P., Guarnieri, P., Caneda, C., Ruderisch, N.*, et al.* (2014b). An RNA-sequencing transcriptome and splicing database of glia, neurons, and vascular cells of the cerebral cortex. The Journal of neuroscience : the official journal of the Society for Neuroscience *34*, 11929-11947.

Figure S1. Related to Figures 2 and 3.

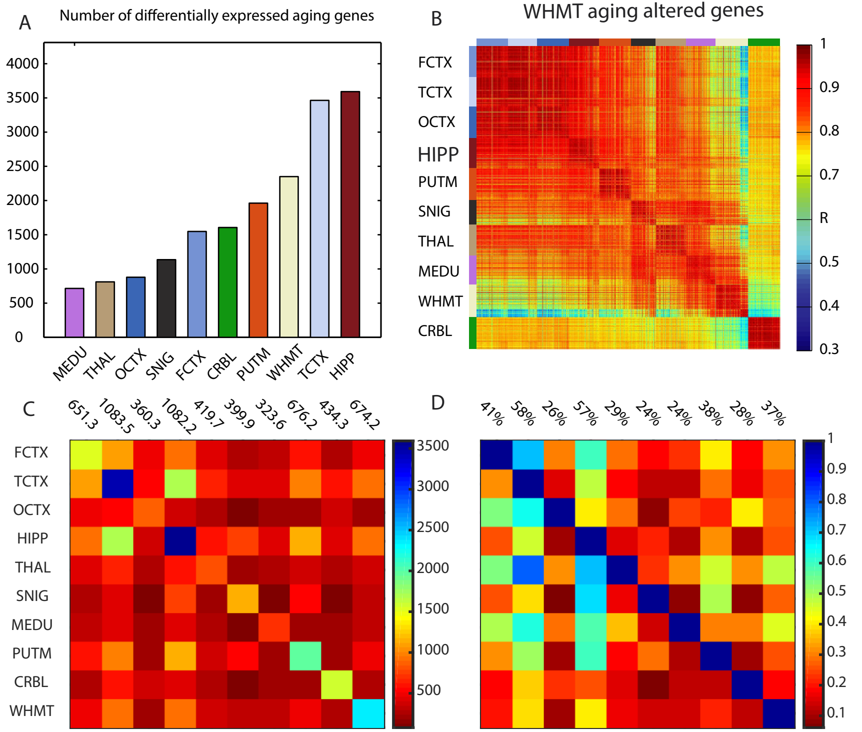

Figure S1

Related to Figures 2 and 3.

A) The number of altered genes per brain region in the main dataset of UKBEC (FDR<1e-3) B) A correlation between all the analyzed 1,231 UKBEC samples based on gene expression signals of genes that were altered upon aging in the white matter (WHMT). C) A heatmap showing the number (left) and percent (right) of brain aging altered genes that were commonly altered in two brain regions of the 10 studied concurrently.

Figure S2. Related to Figure 3.

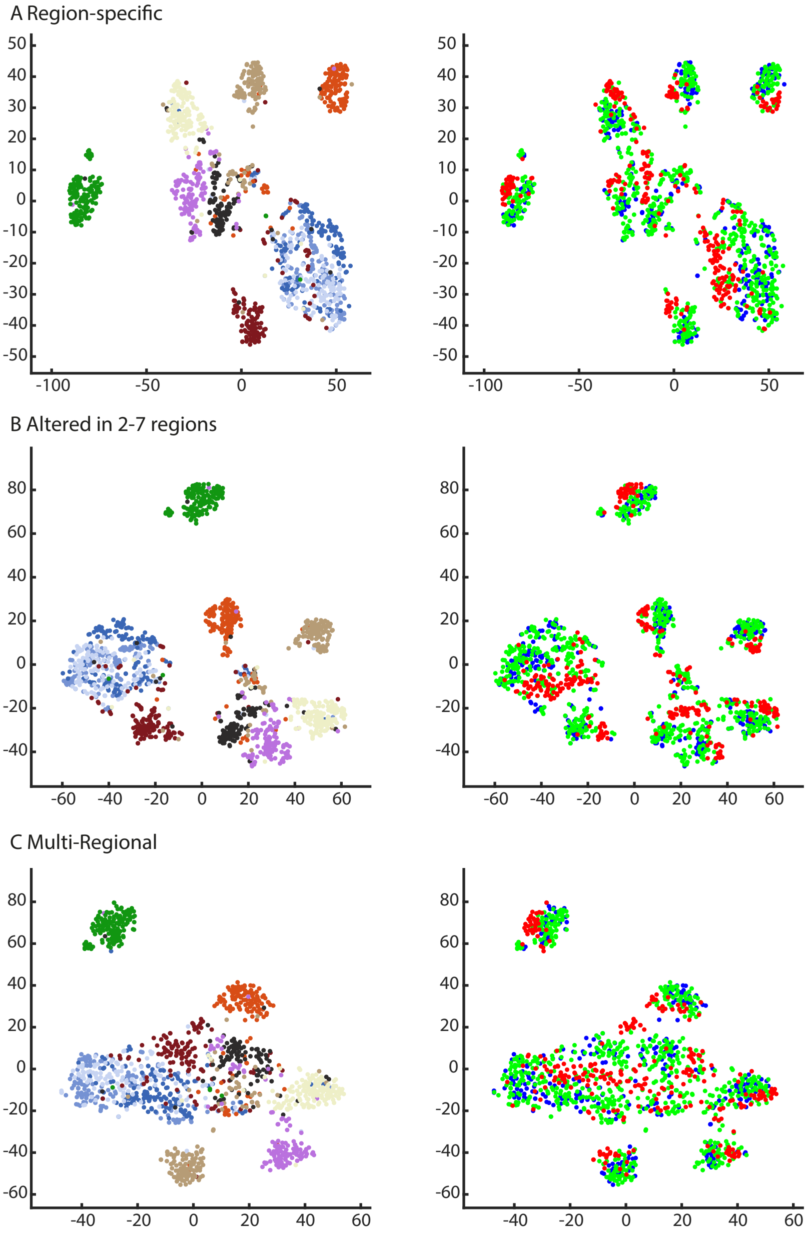

Figure S2

Related to Figure 3.

Classification by a multi-dimensional reduction classification algorithm of the UKBEC samples based on the expression patterns of genes altered in a single brain region, commonly altered in 2-7 brain regions and in 8-10 regions upon aging (B). The samples are much less separated by region based on expression of the multi-regional altered genes (commonly altered in 2-8 regions). In each sub section, the right panel presents the samples colored by age group (young, middle, old).

Figure S3. Related to Figure 3.

A SVM on NABEC based on cross-regional genes

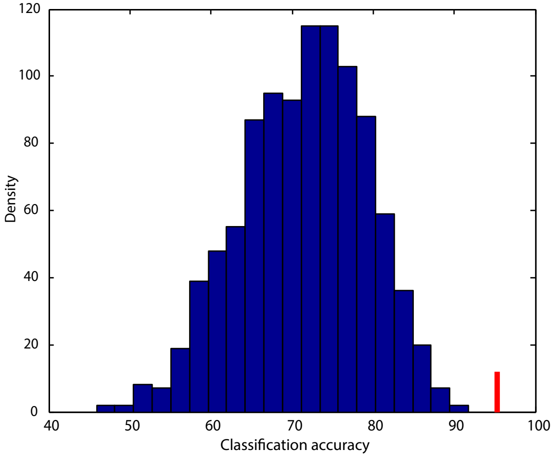

B Age-group classification colored by gender based on cross-regional genes

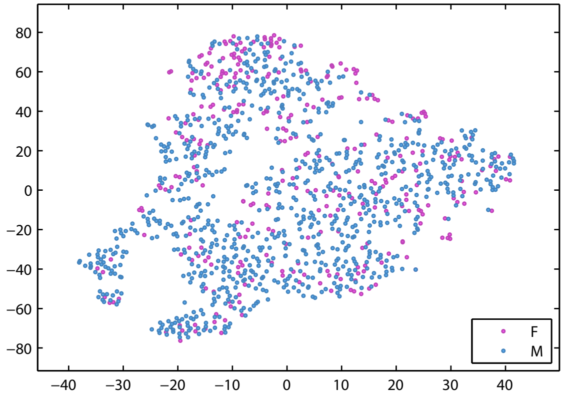

C REST dataset age-group classification by cross-regional genes

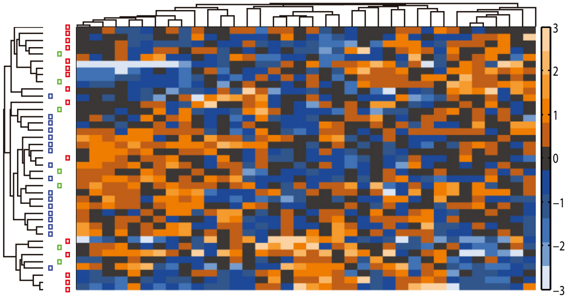

Figure S3

Related to Figure 2.

Support vector classification (SVM) of the independent NABEC dataset based on the expression patterns of a small subset of genes commonly altered in all the analyzed UKBEC brain regions (A). (B) The UKBEC samples are not classified by gender based on the RNA expression profiles of the small subset of aging altered common genes. (C) The second independent dataset (REST dataset) samples are largely classified by age group based on the expression profiles of the small subset of shared genes.

Figure S4. Related to Figures 3 and 7.

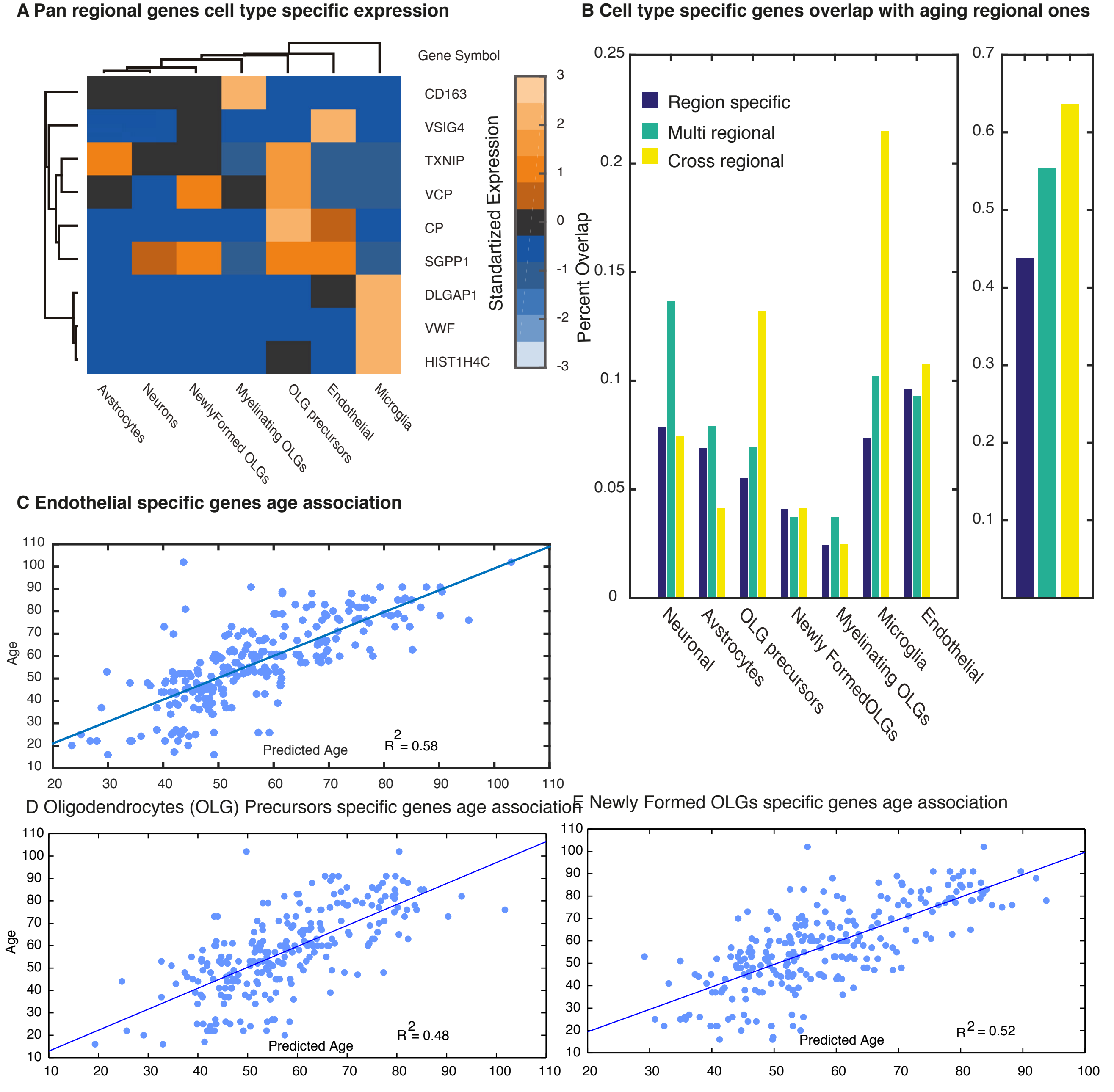

Figure S4

Related to Figures 3 and 7.

The pan-regional aging altered genes were enriched in 7 cell type (RNASeq data (from http://web.stanford.edu/group/barres_lab/brain_rnaseq.html). The hierarchical classification Euclidian distance dendograms are shown as well. Color scale: z-score of count data. B) The single-regional aging genes were more enriched in neuronal specific genes, while multi regional aging genes were more enriched in OLG precursor and microglia specific genes (left). Overall, multi-regional genes were more enriched in cell specific markers as compared with both multi- and single- regional genes (right). C) Endothelial (D) OLG precursor and E) Newly formed OLGs specific genes are associated with age. OLG: oligodendrocytes.

Figure S5. Related to Figure 4.

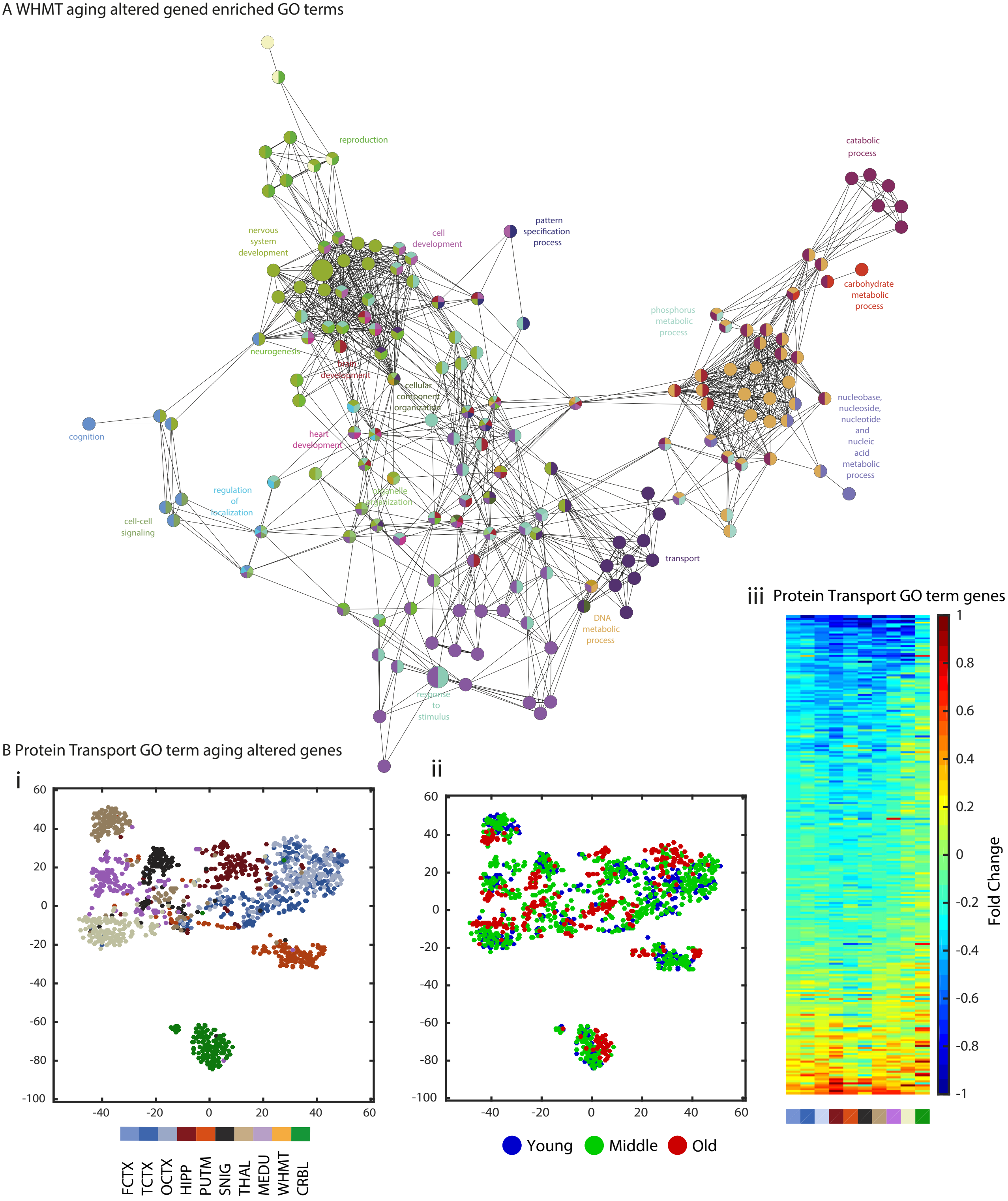

Figure S5

Related to Figure 4.

A) (i) A heatmap of expression of the top 50 MG specific aging altered genes show mainly up regulation ii) The top 50 neuronal specific markers showed a global down regulation, both similarly to the top 100 specific markers (iii) AC specific aging altered genes showed specific down regulation in the SNIG and PUTM iv) The top 50 OLG specific markers showed a mixed pattern of change in aging. In all the panels of A, the left plots show average expression in samples < 60 years old, and the middle plots of > 80 years old. The right panel plots show the fold change ratio (log2) between them (old vs. young). B) (i) A heatmap based on expression signals of top 100 OLG precursor specific aging altered genes (on the z score of the expression values) ii) Classification of the UKBEC samples based on the expression profiles of the top OLG precursor specific aging altered genes, the samples are colored by either brain region (ii) or age group (iii). C) (i) A heatmap based on expression signals of the top 100 Endothelial specific aging altered genes (ii) A classification of the samples based on the expression profiles of top 10 OLG precursor specific aging altered genes, the samples are colored by either region or age group as in A. D) (i) A heatmap of z-score normalized expression signals of the top 100 newly formed OLG specific aging altered genes. ii) Classification based on the top 100 newly formed OLG specific genes, colored by Region (i) and Age (ii). OLG: as in Figure S3 legend.

Figure S6. Related to Figure 4.

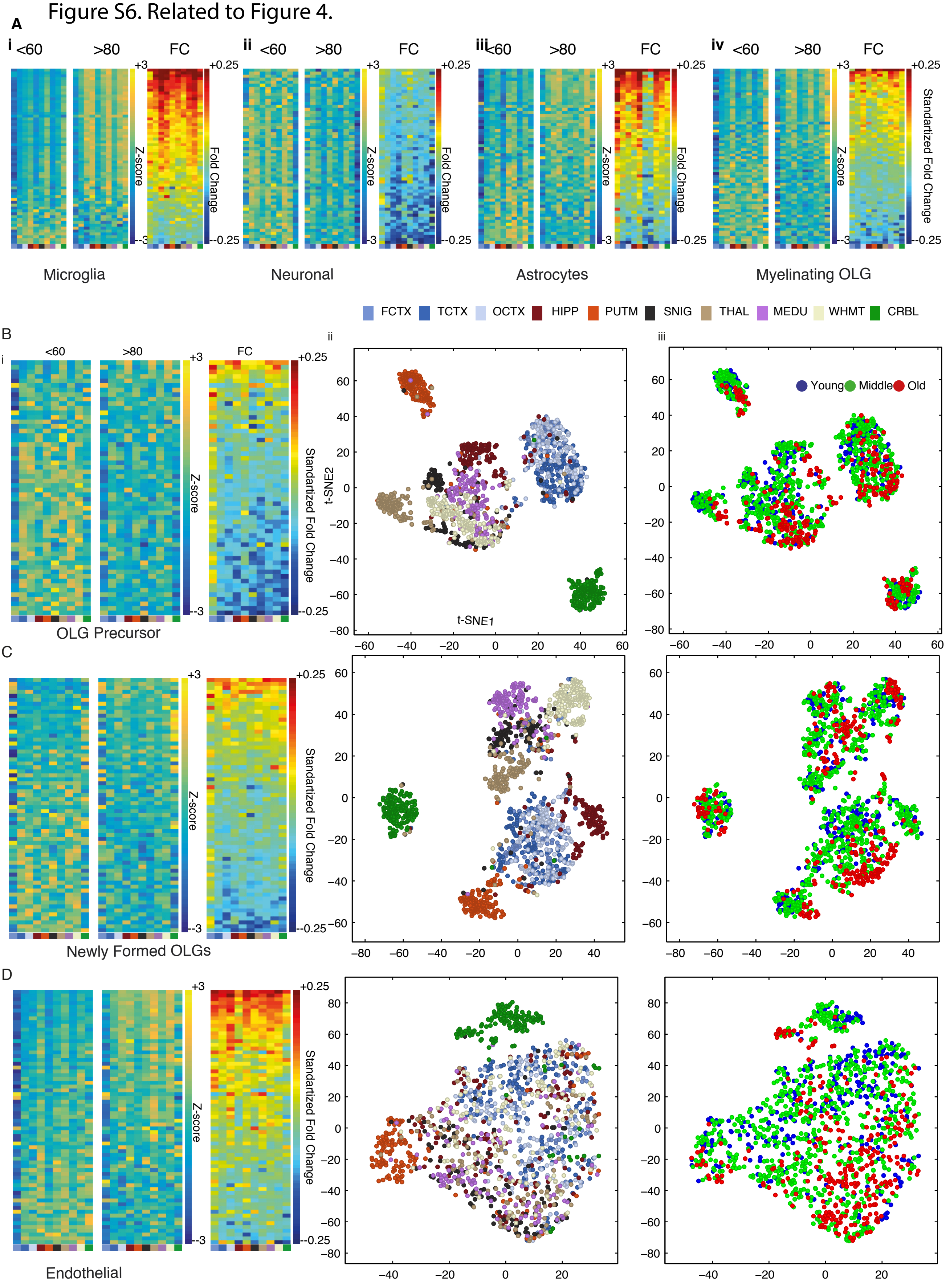

Figure S6

Related to Figure 4.

A) A network based on Gene Ontology (GO) functional enrichment analysis for the WHMT aging altered genes (the analysis was conducted with the Cytoscape plug-in ClueGO (Bindea et al., 2009)). The nodes (functional terms) are colored by functional group. The network is presented in a circular format. B) (i) Classification of all the samples based on the expression of aging altered genes annotated to the GO category protein transport, the samples are painted by regional identity and (ii) by age group. (iii) A heatmap of protein category aging altered genes show mainly down regulation upon aging.

Figure S7. Related to Figure 4 and Figure 7

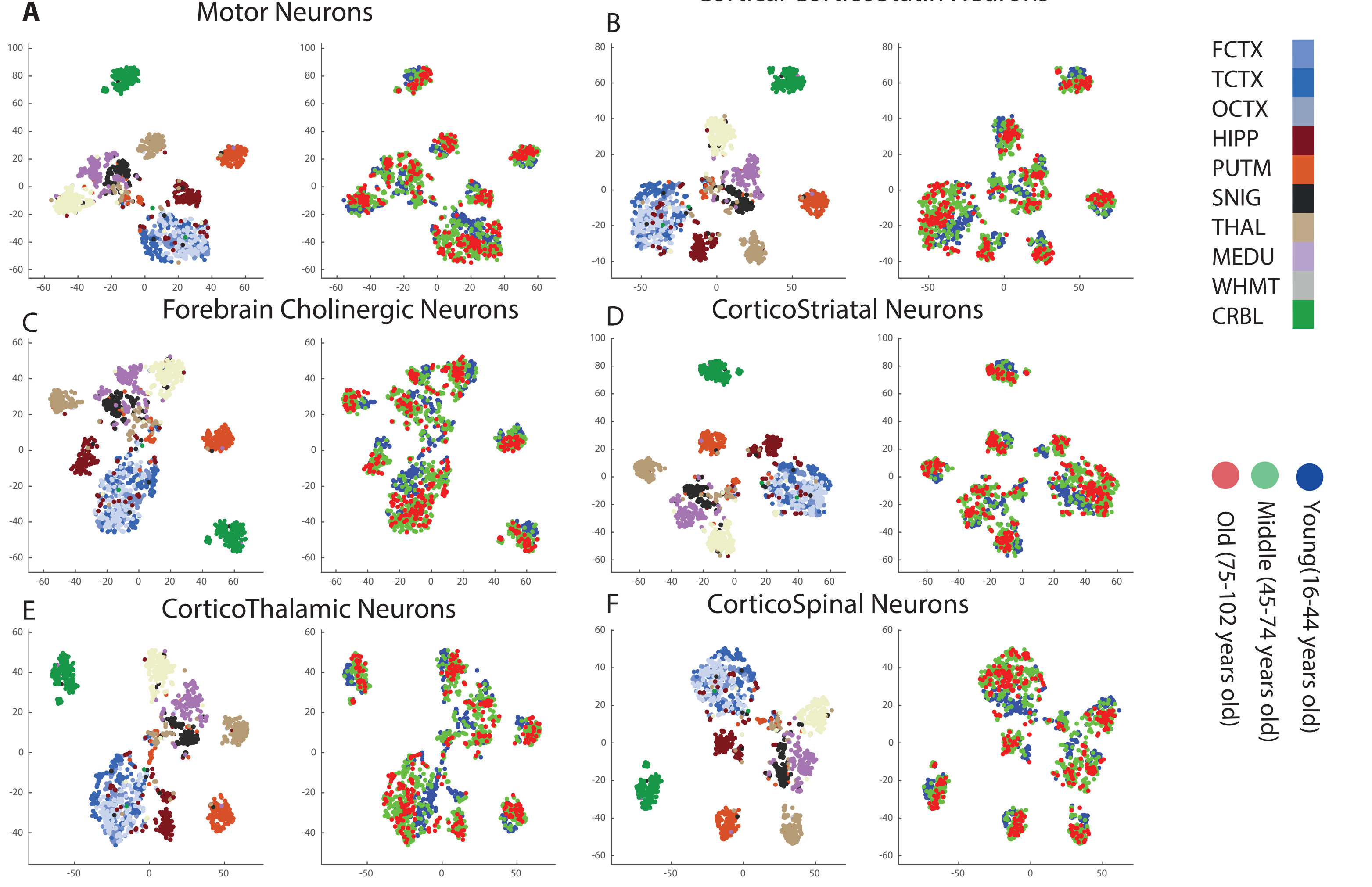

Figure S7

Related to Figure 4 and Figure 7

Classification plots of the UKBEC brain samples based on the expression signals of the neuronal populations specific genes characterized by a previous microarray study on 24 populations (Doyle et al., 2008). Given are examples for 6 different types of neurons. The regional classification showed distinction between the different anatomical regions similarly to one another (and to the plots of neuronal gene markers that were based on our lists that were defined by analysis of publically available rat brain RNASeq data, from http://web.stanford.edu/group/barres_lab/brain_rnaseq.html). Age-group separation was seen in some of the regions (in particular, the PUTM and WHMT as well as CRBL for motor neurons). The classification plots show classification of the samples based on the following aging altered neuronal markers: motor neurons specific genes, cortical CorticoStatin neurons, forebrain cholinergic neurons, corticostriatal neurons, corticoThalamic neurons and corticoSpinal neurons specific genes (A-F). CRBL: cerebellum, PUTM: putamen, WHMT: white matter.

Table S4. Cross-regional aging altered genes (commonly altered in 10 human brain regions)

Soreq L. et al., Major shifts in glial regional identity are a transcriptional hallmark of human brain aging

Related to Figure 3.

#	Gene Symbol	Gene Full Name	Cellular component/ cell specificity	Literature evidence for aging involvement*?	Literature evidence for neuro/ psychiatric disease involvement?
1	TXNIP	Thioredoxin interacting protein	Cytoplasm	Yes (PMID - 23958415, 22661500)	Yes (AD) - 22482078
2	CP	Ceruloplasmin (ferroxidase)	Astrocytes	Yes (18977241, and 1761530 – cognitive aging)	Yes (PD - 16150804, MS - 23868451)
3	HIST1H4C	Histone cluster 1, H4c	Nucleous	Yes (susceptible, (20800603)	
4	MPZL2 (EVA)	Myelin protein zero-like 2	Membrane, cytoskeleton		
5	VWF	Von Willebrand factor	Endoplasmatic reticulum, extracellular matrix		Yes (22120183)
6	CD163	Macrophage-Associated Antigen	Cell membrane/ Monocytes and Macrophages		Yes (MS, 21737148)
7	SGPP1	Sphingosine-1-phosphate phosphatase 1	Endoplasmic reticulum membrane		Yes (Schizophrenia, 18683247)

8	FLJ35776 (DLGAP1-AS1)	DLGAP1 antisense RNA 1	Uncharacterized * LncRNA		
9	VSIG4	V-set and immunoglobulin domain containing 4	Macrophages		

Supplementary Table 4| Cross regional human aging altered genes, related to Figure 3

This supplemental table is included within this supporting information file. The cross-regional aging altered genes that were found as significantly altered in all the analyzed 10 brain regions upon aging. The table includes the full gene names, literature evidence for involvement in aging (if any) and cellular specificity. Reference in the main paper text: page 13, line 21.
