# Supplementary material for: Major Shifts in Glial Regional Identity Are a Transcriptional Hallmark of Human Brain Aging
Source: Cell Rep. 2017 Jan 10;18(2):557–70. doi: 10.1016/j.celrep.2016.12.011 (PMC5263238; doi:10.1016/j.celrep.2016.12.011)
Supplement: Document S2. Article plus Supplemental Information [file mmc8.pdf]

Cell Reports

Resource

Major Shifts in Glial Regional Identity Are a Transcriptional Hallmark of Human Brain Aging

Graphical Abstract

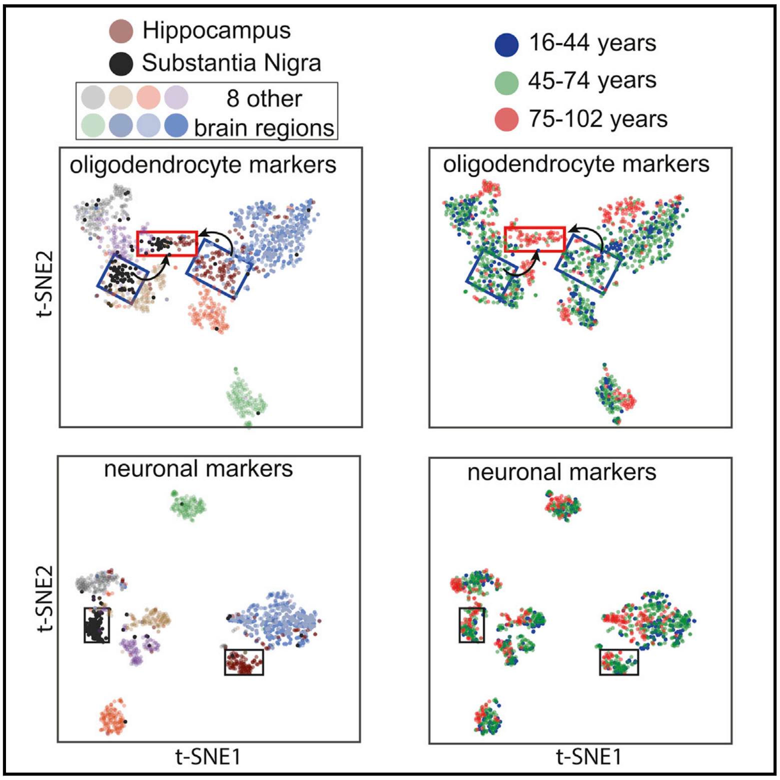

Highlights

- Understanding the role of cell-type-specific changes in human brain aging
- Glial-specific genes shift their regional expression patterns during aging
- Oligodendrocytes and neuronal subpopulations are decreased in the aging neocortex
- Microglia-specific genes globally increase their expression during aging

Authors

Lilach Soreq, UK Brain Expression Consortium, North American Brain Expression Consortium,, ..., Mina Ryten, Rickie Patani, Jernej Ule

Correspondence

rickie.patani@ucl.ac.uk (R.P.),
j.ule@ucl.ac.uk (J.U.)

In Brief

Human brain aging is determined by a complex interplay of regional and cell-type-specific molecular events. Soreq et al. find that glial genes shift their regional expression patterns, while microglia-specific genes globally increase their expression upon aging. Moreover, immunohistochemistry reveals decreased numbers of oligodendrocytes and neuronal subpopulations in the aging neocortex.

Accession Numbers

GSE46706
GSE36192

Soreq et al., 2017, Cell Reports *18*, 557–570
January 10, 2017 © 2017 The Author(s).
http://dx.doi.org/10.1016/j.celrep.2016.12.011

OPEN ACCESS
CellPress

Major Shifts in Glial Regional Identity Are a Transcriptional Hallmark of Human Brain Aging

Lilach Soreq,[1,2] UK Brain Expression Consortium, North American Brain Expression Consortium, Jamie Rose,[3] Eyal Soreq,[4] John Hardy,[1,5] Daniah Trabzuni,[1,6] Mark R. Cookson,[7] Colin Smith,[3] Mina Ryten,[1,9] Rickie Patani,[1,2,5,8,10,*] and Jernej Ule[1,2,11,*]

[1]Institute of Neurology, University College London, London WC1N 3BG, UK
[2]The Francis Crick Institute, 1 Midland Road, London NW1 1AT, UK
[3]MRC Edinburgh Brain Bank, Academic Neuropathology, Centre for Clinical Brain Sciences, University of Edinburgh, Edinburgh EH16 4SB, UK
[4]The Computational, Cognitive and Clinical NeuroImaging Laboratory, Division of Brain Sciences, Imperial College, London SW7 2AZ, UK
[5]Reta Lila Weston Institute of Neurological Studies, UCL ION, 1 Wakefield Street, London WC1N 1PJ, UK
[6]Departments of Genetics, King Faisal Specialist Hospital and Research Centre. Riyadh 12713, Saudi Arabia
[7]Laboratory of Neurogenetics, National Institute on Aging, NIH, Bethesda, MD 20892, USA
[8]Euan MacDonald Centre for MND, University of Edinburgh, Edinburgh EH8 9YL, UK
[9]Department of Medical and Molecular Genetics, King's College London, Guy's Hospital, Great Maze Pond, London SE1 9RT, UK
[10]Department of Clinical Neurosciences, University of Cambridge, Cambridge CB2 1TN, UK
[11]Lead Contact
*Correspondence: rickie.patani@ucl.ac.uk (R.P.), j.ule@ucl.ac.uk (J.U.)
http://dx.doi.org/10.1016/j.celrep.2016.12.011

SUMMARY

Gene expression studies suggest that aging of the human brain is determined by a complex interplay of molecular events, although both its region- and cell-type-specific consequences remain poorly understood. Here, we extensively characterized aging-altered gene expression changes across ten human brain regions from 480 individuals ranging in age from 16 to 106 years. We show that astrocyte- and oligodendrocyte-specific genes, but not neuron-specific genes, shift their regional expression patterns upon aging, particularly in the hippocampus and substantia nigra, while the expression of microglia- and endothelial-specific genes increase in all brain regions. In line with these changes, high-resolution immunohistochemistry demonstrated decreased numbers of oligodendrocytes and of neuronal subpopulations in the aging brain cortex. Finally, glial-specific genes predict age with greater precision than neuron-specific genes, thus highlighting the need for greater mechanistic understanding of neuron-glia interactions in aging and late-life diseases.

INTRODUCTION

Aging, an inevitable time-dependent functional decline, is present in all living organisms. The intimate relationship between aging and neurodegeneration raises the possibility of shared transcriptional and post-transcriptional gene regulation programs; however, we still lack a comprehensive transcriptome-wide picture of the effects of aging across different human brain regions and cell types (De Strooper and Karran, 2016). RNA expression profiling of the aging brain has been studied historically using a limited number of brain regions in animal models or human post-mortem tissues. A major unrealized goal therefore remains a comprehensive characterization of the transcriptional landscape across multiple human brain regions in a physiological age range, which may provide insights into the cellular architecture and molecular pathways of aging.

The unparalleled complexity of the human brain is a function of its structural and functional cellular diversity, which arises from tightly regulated transcriptional programs. Limited availability to human post-mortem samples has hampered comprehensive transcriptomic analysis of the brain, particularly of region- and cell-type-specific diversity. However, through international collaboration, a comprehensive atlas of the brain's transcriptome based on samples from two individuals (the Allen Brain Atlas) has been achieved. This study illustrated how transcripts of genes involved in different pathways are expressed across the brain, but the potential effect of age on the regional differences was not examined.

By current consensus, astrocyte (AC) and neuronal numbers appear generally preserved in aging (Fabricius et al., 2013; Matarin et al., 2015; Pelvig et al., 2008). It is clear, however, that Alzheimer's disease (AD) and other neurodegenerative diseases for which age is a major risk factor are associated with inflammatory changes mediated by microglia (MG) (Cribbs et al., 2012; Frank et al., 2008). Brain aging includes accumulation of senescent MG, altered signaling, and pro-inflammatory phenotypes (Mosher and Wyss-Coray, 2014), and it was shown that MG display regional sensitivity to aging (Streit and Xue, 2010). Immune-related changes were also strongly associated with aging in mouse models of amyloid pathology (Matarin et al., 2015). Nevertheless, animal models and human tissue have reported

© 2017 The Author(s).
This is an open access article under the CC BY license (http://creativecommons.org/licenses/by/4.0/).

OPEN
ACCESS
CellPress

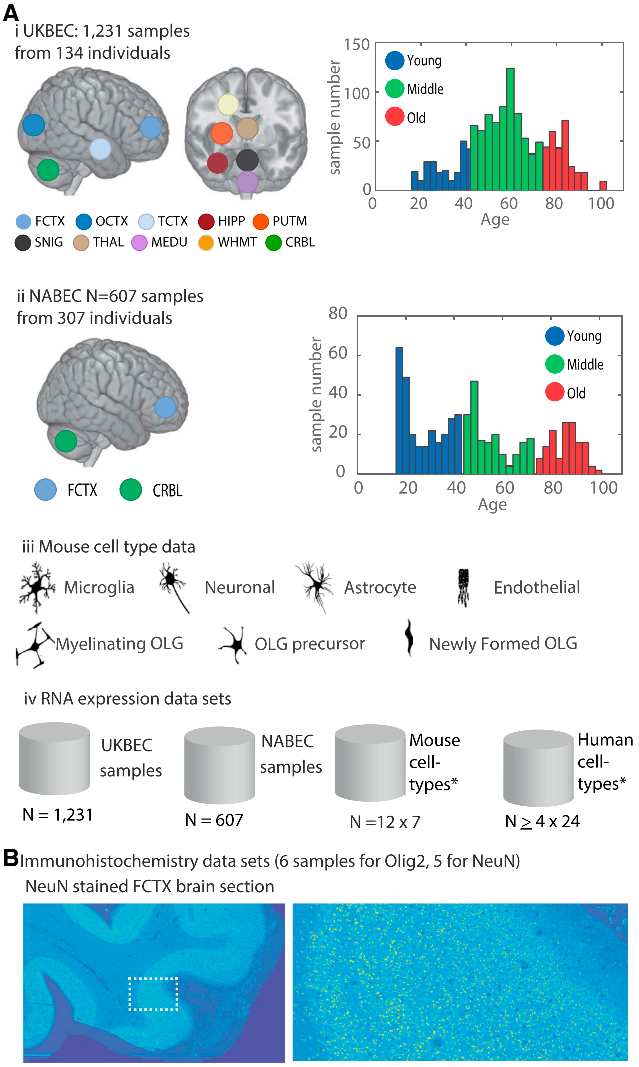

Figure 1. Analyzed Samples and Datasets

(A) The samples of the UKBEC and NABEC datasets were divided into three age groups each (young: 16–44, middle: 45–74, old: ≥75). (i) The main analyzed dataset (UKBEC) is composed of 1,231 brain samples interrogated by exon microarrays, from brain samples of 134 individuals from 16 to 102 years old and up to ten brain regions each. The brain regions included both cortical and sub-cortical regions, specifically: the frontal cortex (FCTX), temporal cortex (TCTX), occipital cortex (OCTX), intralobular white matter (WHMT), cerebellum (CRBL), substantia nigra (SNIG), putamen (PUTM), thalamus (THAL), hippocampus (HIPP), and medulla (MEDU) for UKBEC and the FCTX and CRBL for NABEC. (ii) The independent (NABEC) dataset of brain samples from FCTX and CRBL 307 individuals (16–101 years old). (iii) In addition, seven cell types were identified based on analysis of available RNA-seq data from mice cortex (http://web.stanford.edu/group/barres_lab/brain_rnaseq.html). (iv) A summary of all expression data used in this study. The total number of samples described in (i)–(iii) is listed, as well as the human RNA-seq analysis of 24 CNS human cell types (Table S7) (http://web.stanford.edu/group/barres_lab/brainseqMariko/brainseq2.html).

(B) High-resolution immunohistochemical imaging dataset was produced from samples of young and three old FCTX from the UKBEC cohort, following staining by OLIG2 antibody and computational analysis for the quantification of the OLG cell population. Staining by NeuN of FCTX sections from the same brain samples followed by targeted computational analysis was conducted for quantification of the neuronal cell population (an example of one of the NeuN stained sections is shown on the right, in the zoomed-in view of the area marked on the left-hand side). OLG, oligodendrocyte.

variable and apparently contrasting alterations in ACs (reactivity or atrophy) and MG (MHC class II antigen increase or atrophy) (Cerbai et al., 2012; Streit and Xue, 2010; Tremblay et al., 2012). Accumulation of oligodendrocytes (OLGs) was previously reported in aging monkey cortex (Peters and Sethares, 2004), while stereological quantification of glia in neocortical regions of old brains has suggested a reduction in the number of OLGs, as evident by a >3-fold greater atrophy of the sub-cortical white matter (WHMT) compared to cortical regions and an age-determined loss of myelin (Head et al., 2004; Vernooij et al., 2008). Furthermore, MG-mediated neuroinflammation has been described as a common hallmark of both AD and Parkinson's disease (PD) and is believed to be mechanistically important in driving pathogenesis (Orre et al., 2013; Perry and Teeling, 2013). Collectively, these findings suggest that the field stands to benefit from systematic and comprehensive analysis of aging-related changes in the cellular and molecular composition of the human brain.

Apart from the study of region-dependent microglial response to aging, the importance of both region- and cell-type-specific changes in the aging brain remains poorly understood. Studies have been hampered by the limited availability of cross-regional post-mortem tissue across a range of ages. To overcome these limitations, we analyzed gene expression patterns in ten brain regions (including cortical and sub-cortical areas) using more than 1,800 brain samples from two large independent cohorts, representing the most comprehensive human aging brain gene expression analysis to date. We report striking changes in cell-type-specific expression patterns across different brain regions, which revealed major shifts in glial regional identity upon aging in the human brain.

RESULTS

In this study, we examined two extensive gene expression datasets from post-mortem human samples and sampled multiple (up to ten) brain regions per individual. The primary dataset was produced by the UK Brain Expression Consortium (UKBEC) and included 1,231 tissue samples collected from 134 adult individuals between 16 and 102 years old, with each contributing post-mortem samples of up to ten brain regions (Figure 1Ai). The brain regions included both cortical and sub-cortical regions, specifically the frontal cortex (FCTX), temporal cortex (TCTX), occipital cortex (OCTX), intralobular white matter (WHMT), cerebellum (CRBL), substantia nigra (SNIG), putamen (PUTM), thalamus (THAL), hippocampus (HIPP), and medulla (MEDU). The second dataset, which allowed independent external cross-validation, was produced by the North American Brain Expression Consortium (NABEC) (Gibbs et al., 2010; Kumar et al., 2013), including 307 samples from two brain regions (age range: 16 to 101 years old; Figure 1Aii). The third dataset was also used for validation including samples with an age range of 27 to 106 years old (Lu et al., 2014). None of the brain samples had neuropathological evidence of diagnosable degenerative

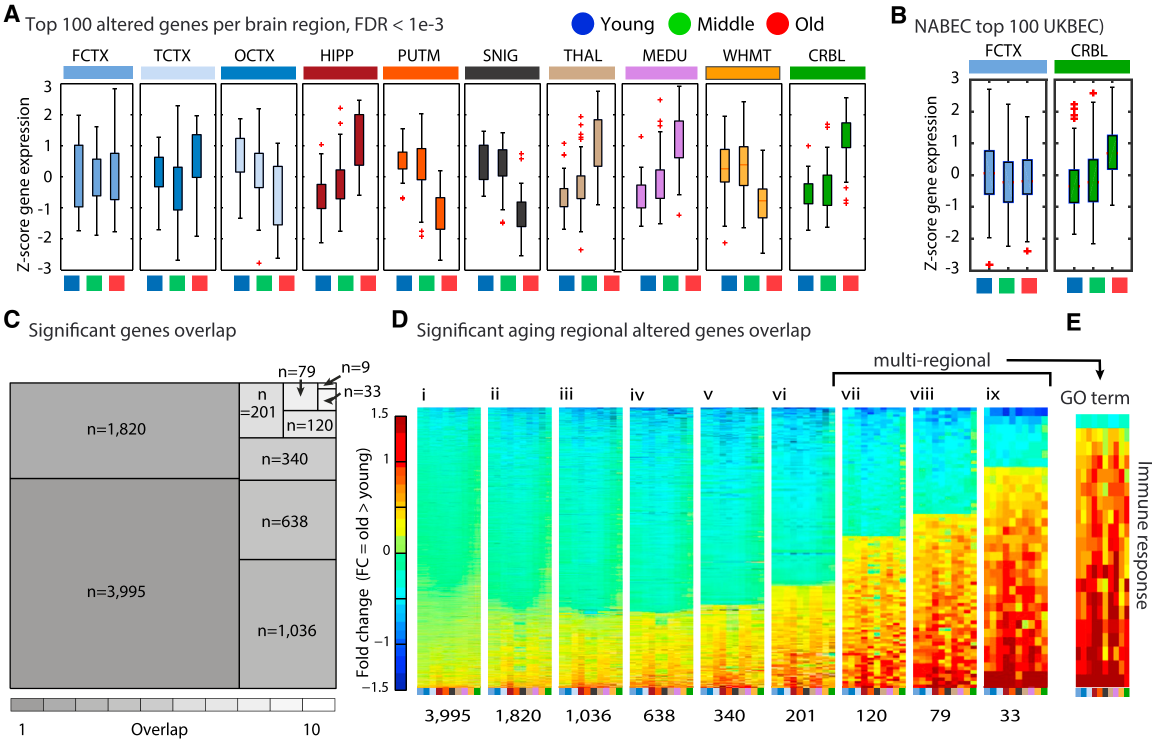

Figure 2. Multi-regional Aging-Altered Genes Are Mainly Upregulated

(A) The direction of expression change of the top 100 genes detected as significantly differentially expressed upon aging in each of the studied expression datasets from ten UKBEC brain regions (ANOVA test significance threshold: FDR < 1e−3; the test compared the three defined age groups).

(B) Age-group based separation of 607 FCTX and CRBL samples (the NABEC cohort) was based on measured expression of the nine cross-regional genes.

(C) A tree map of the number of genes that were altered upon aging, dependent on the number of brain regions where the change is observed.

(D) Fold change of the genes that were altered upon aging, separated into heatmaps dependent on the number of brain regions where the change is observed.

(E) Fold change of the multi-regional genes that were enriched in the Gene Ontology term immune response (standardized Z score; range is as shown for the heatmaps on the left).

Brain region abbreviations are explained in the legend to Figure 1A. See also Figure S1A for the total number of aging-altered genes per region.

diseases (Table S1). To detect differentially expressed genes, we assigned each sample to one of three age groups (young: 16–44, middle: 45–74, old: ≥75 years old) and applied a collection of tailored data-mining computational approaches (Figure 1). We excluded gender-based sample separation to specifically identify the effects of age on gene expression profiles.

Region-Specific and Global Transcriptional "Signatures" of the Aging Human Brain

We first sought to address whether region-specific differences in gene expression patterns occur within the brain upon aging. Both the number of differentially expressed genes (threshold: false discovery rate [FDR] < 1e−3) (Figure S1) and the direction of expression change varied in a region-specific manner (Figure 2A). The general directions of gene expression change were preserved in the independent NABEC dataset (Figure 2B). We applied a stringent threshold to enable isolation of global changes across the UKBEC brain regions. Most changes were specific for one region (hereafter referred to as "region specific") or a few regions (hereafter referred to as "region selective," including genes altered in two to seven regions), while some genes were altered in eight or more brain regions (hereafter referred to as "multi-regional"), and nine genes were found to be significantly altered in all ten brain regions upon aging (FDR < 1e−3, hereafter referred to as "cross-regional") (Figure 2C; Figure S2). The rates and number of overlapping age-altered genes varied between pairs of brain regions (Figure S2). Multi-regional genes predominantly exhibited increased expression levels upon aging (Figures 2Dviii–2Dix). This group of genes was enriched in the Gene Ontology (GO) term "immune response", which had the general trend to be upregulated in aging (Figure 2E).

Nine Cross-Regional Aging-Altered Genes Accurately Predict the Age Categories

To assess the ability of different groups of genes to classify the brain samples in both cohorts by brain region and age group criteria, we next used a non-linear dimension reduction classification method. The expression of regional-selective genes separated the samples well based on their regional identity (Figure S2). In addition, genes with aging-altered expression patterns in the CRBL, WHMT, or cortical samples generally have distinctive regional expression, as evident by sample-to-sample correlation scores that were computed among each of the 1,231

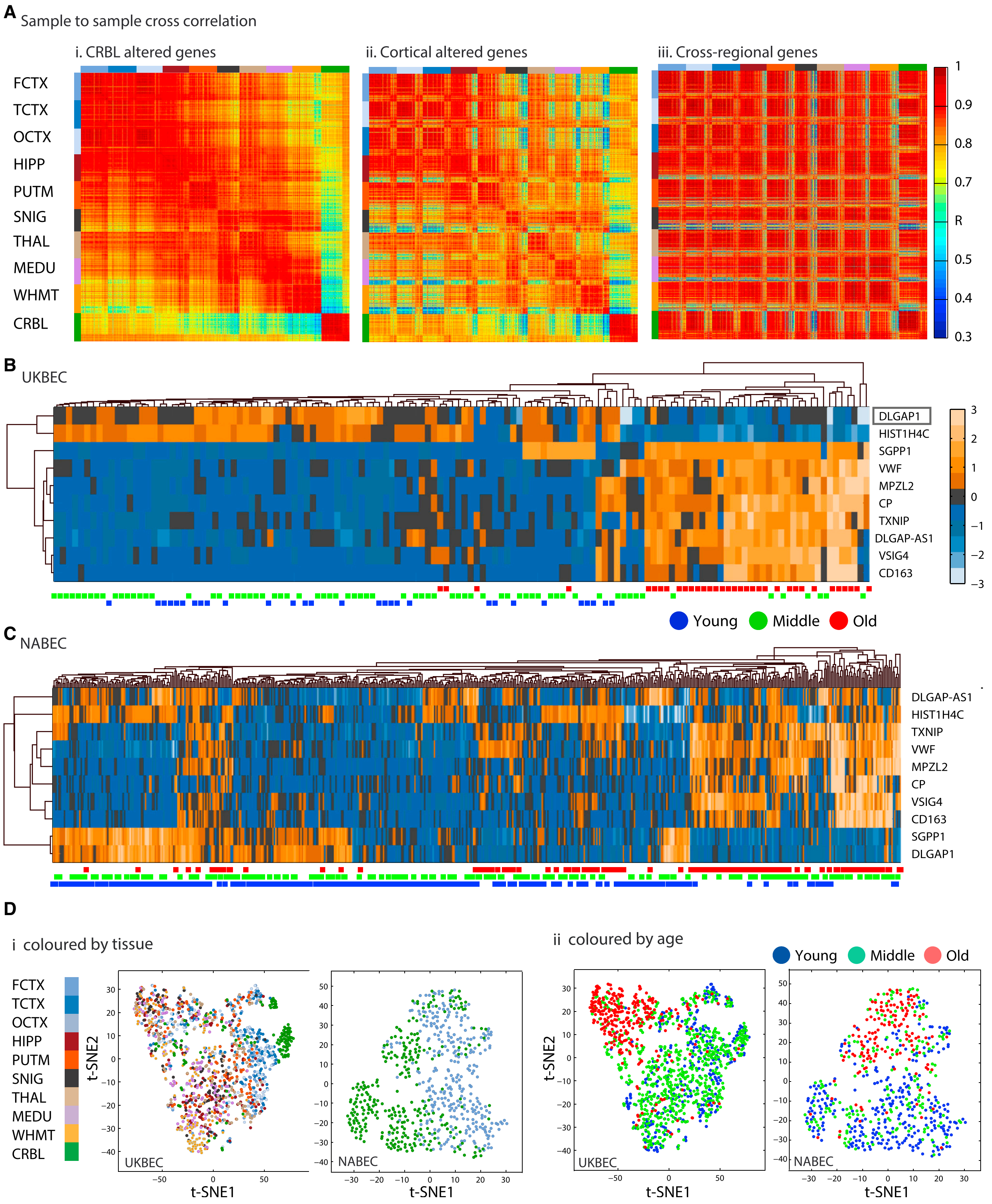

(legend on next page)

brain samples based on their expression signals (Figures 3Ai and Aii; Figure S1B). The 642 CRBL-altered genes showed high inter-regional correlation in expression patterns with the cortical regions (including the HIPP), the 265 cortical aging-altered genes revealed high correlation among the cortical regions and HIPP samples, and the 801 WHMT-altered genes showed increased correlation specifically within CRBL and WHMT.

In contrast to region-specific aging-altered genes, expression patterns of these cross-regional genes were correlated among samples only based on age, rather than brain region (Figure 3Aiii). Eight of the pan-regional genes were upregulated, and one (*HIST1H4C*) was downregulated upon aging (Figure 3B; Table S5). The cross-regional genes successfully discriminated the samples based on age group in most UKBEC cases (130 of 134) (Figure 3B). One of these genes was a non-coding RNA (*DLGAP1-AS1*) that is antisense to the protein-coding gene (*DLGAP1*). We identified a robust, reciprocal relationship in expression between *DLGAP1* and *DLGAP1-AS1* upon aging. *DLGAP1* is highly brain-specific, while *DLGAP1-AS1* and *DLGAP1-AS2* are normally expressed in internal organs and the bone marrow (Gene Cards database) (Harel et al., 2009). We find that in contrast to upregulation of *DLGAP1-AS*, expression of *DLGAP1* shows an aging-altered decrease across brain regions, despite not reaching statistical significance in each region (Figure 3B). This demonstrates coupling between the age-dependent decrease in the expression of the brain-specific protein-coding gene DLGAP1 and the increase of its antisense RNA, which is otherwise only expressed outside of the brain.

As a sign of the validity of the cross-regional genes, they were efficient in classifying samples of the independent NABEC dataset based on age, even though the NABEC data were not used to identify these genes (Figure 3C). Moreover, a non-linear classification based on these genes separated samples belonging to young, middle-age, and old groups in the UKBEC, the NABEC, and an additional independent FCTX brain expression cohort (Lu et al., 2014) (Figure 3D; Figure S3C). This model also verified the prediction of age group based on expression levels in the NABEC cohort (Figure S3A). To exclude the effects of other variables, we show that the UKBEC samples were not classified by gender (Figure S3B). The cross-regional genes also correctly classified samples by age group in an additional independent dataset of cortical samples (Lu et al., 2014; Figure S3) and did not classify the UKBEC dataset by gender (Figure S3).

Major Shifts in Region-Specific Expression Profiles of Glia-Specific Genes in the Aging Brain

To investigate the biological relevance of the age-related gene expression changes, we first examined the expression profiles of the cross-regional genes in recently produced RNA sequencing (RNA-seq) data from seven purified mouse brain cell types (Zhang et al., 2014). All cross-regional genes were expressed in a cell-type-specific manner, in particular within glial cells and mainly in MG and OLGs (Figure S4A). We therefore further examined the cell-type-specific expression patterns of all aging-altered genes. For this, we calculated genome-wide expression scores to identify genes specific for each cell type (Table S3). We then examined whether expression of cell-type-specific genes was altered upon aging. For three cell types (neurons, ACs, and OLGs), enrichment data previously generated by microarrays was also available (Cahoy et al., 2008). We selected genes that were demonstrated to be specific by both our defined cell-specific lists found by analysis of RNA-seq data (Zhang et al., 2014) and the published microarray cell-specific lists from mice (Cahoy et al., 2008), in addition to being altered in aging. In agreement with the great diversity of neuronal cell types across different brain regions, neuron-specific genes were most enriched among the regional aging-altered genes (Figure S4B). In contrast, glia-specific genes were most enriched among the multi- and cross-regional altered genes, and this was most pronounced for OLG precursors and MG (Figure S4B).

We first sought to investigate the changes in expression of MG-specific genes, because these cells have been most extensively linked to aging so far (Erraji-Benchekroun et al., 2005; Sibille, 2013). Consistent with previous studies (Erraji-Benchekroun et al., 2005; Sibille, 2013), most MG-specific aging-altered genes had low expression in brain samples from the young group but strongly increased their expression in all regions in the old group samples (Figure S5A). Our study extends these findings to a multi-regional phenomenon. Moreover, we find that a small number of MG-specific genes have high absolute expression in the young group but decreased expression upon aging in all regions. This suggests that the change in expression not only reflects an increased number of MG but most likely also includes a dramatic change in the MG gene expression program. Although most brain regions were not strongly separated by the MG-specific aging-altered genes, the CRBL was distinct (Figure 4A). This complies with a report of a distinct MG expression profile in the CRBL of young mice (Grabert et al., 2016).

Figure 3. The Nine Cross-Regional Genes Discriminate Samples Based on Age

(A) Correlation scores were calculated between each pair of brain samples among the UKBEC samples based on different lists of aging-altered genes using Spearman correlation. (i) Correlation scores based on CRBL aging-altered genes. (ii) Correlation scores based on cortical aging-altered genes. (iii) Correlation scores based on cross-regional aging-altered genes. See Figure S1B for correlation based on WHMT-altered genes.

(B) Hierarchical classification of the UKBEC cohort based on the expression signals of the cross-regional altered genes and the *DLGAP* antisense. Rows, genes; columns, samples. Age group is denoted in blue (young: 16–44 years), green (middle-age: 45–74 years), or red (old: $\geq$75 years). The dendrograms show the Euclidian distance measured for both rows and columns. Right color bar, standardized fold change (*Z* score; range: -3 to 3; orange represents increased expression in aging, and blue denotes decrease).

(C) Hierarchical classification of the NABEC expression dataset based on the profiles of the cross-regional genes and the DLGAP antisense.

(D) Non-linear dimensionality reduction by t-distributed stochastic neighbor embedding (t-SNE) is based on the expression of the nine cross-regional genes, with the x axis showing t-SNE1 and the y axis showing t-SNE2. Either the ten UKBEC brain regions or the two NABEC brain regions (FCTX and CRBL) are classified, as marked in the plots. (i) Each sample is colored based on its corresponding tissue (colors are marked on the left). (ii) The same samples are colored based on their age group (colors are marked on the top).

Brain region abbreviations are explained in the legend to Figure 1A.

OPEN
ACCESS
CellPress

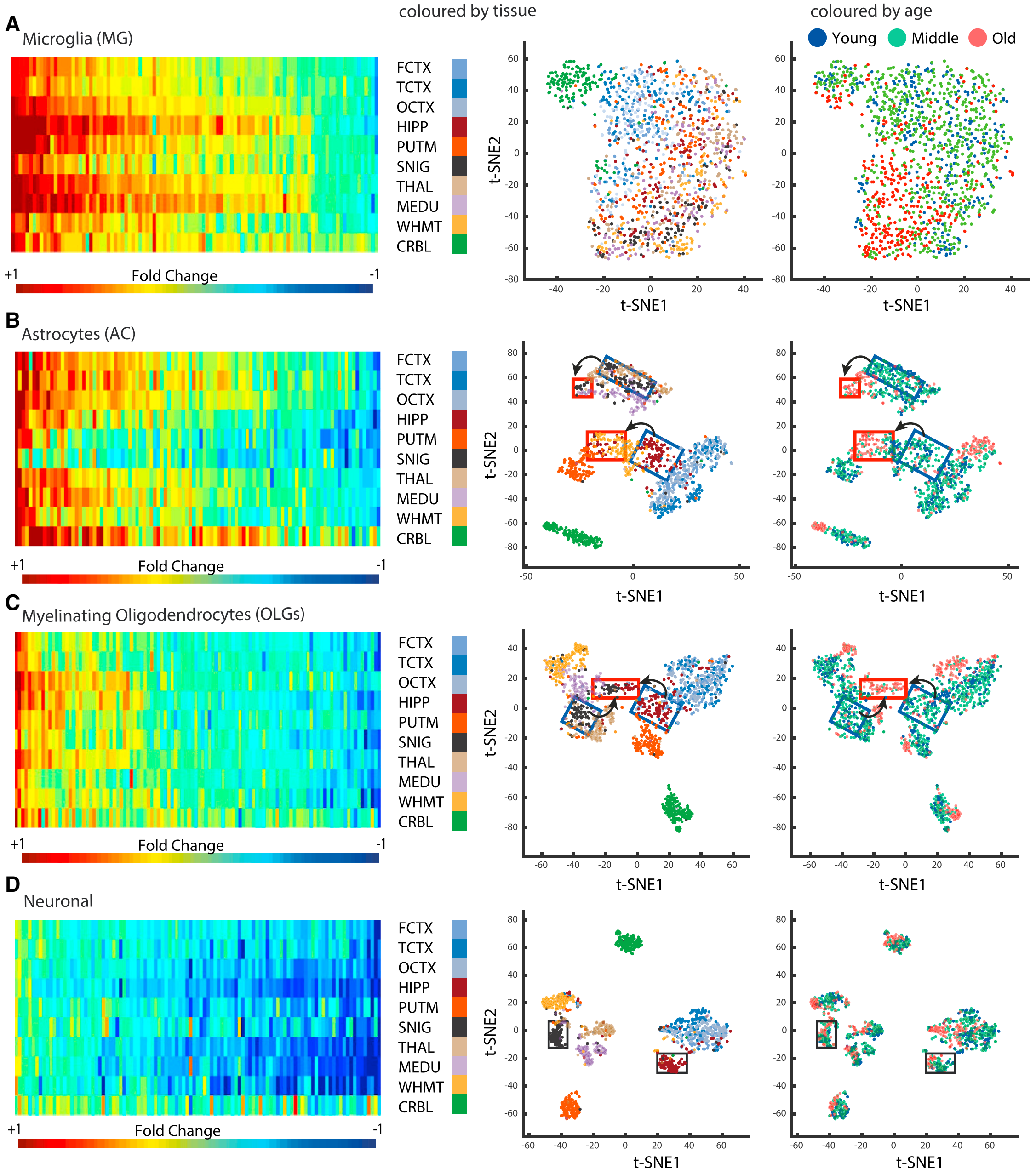

Figure 4. Glia-Specific Genes Show Major Shifts in Regional Identity upon Aging

On the left, heatmaps show the fold change between old and young groups in the expression of the top 100 aging-altered cell-type-specific genes across regions (the color bar corresponds to the standardized Z score, with blue corresponding to decrease and red to increase in gene expression; range: -1 to $+1$). On the right, non-linear dimensionality reduction by t-distributed stochastic neighbor embedding (t-SNE) is used to classify a sample of the ten UKBEC brain regions based on the expression of the top 20 aging-altered cell-specific genes, with the x axis showing t-SNE1 and the y axis showing t-SNE2. In the first plot, each sample is colored based on its corresponding tissue (colors are marked on the left of the plot), and in the second plot, the same samples are colored based on their age group (colors are marked on the top of the plots).

(A) Sample classification based on the aging-altered MG-specific genes.

(B) Sample classification based on the aging-altered AC-specific genes.

(C) Sample classification based on aging-altered myelinating OLG-specific genes.

(D) Sample classification based on the expression signals of aging-altered neuron-specific genes. The SNIG and PUTM samples are marked by rectangles as an example of the loss of region-specific expression upon aging for OLG- and AC-specific genes.

Brain region abbreviations are explained in the legend to Figure 1A. MG, microglia; AC, astrocyte; OLG, oligodendrocyte. See Figure S2 for sample classification based on region-specific genes compared with multi-regional genes and Figure S6 for heatmaps and classification plots based on the three cell-type microarray gene markers.

Apart from the CRBL samples that formed a separate cluster, samples from the old group clustered together for all other regions and separately from younger samples, indicating that MG-specific gene expression is more defined by age than by regional identity (Figure 4A). The aging-altered endothelial-specific genes showed a similar pattern of changes as the MG-specific genes, with a general upregulation across all brain regions and a notable age group separation and lack of clear regional identity (Figure S5D).

Next, we examined the expression profiles of genes specific for either ACs or OLGs. In young samples, we observed much higher absolute expression of AC-specific genes in the midbrain regions compared to cortex and HIPP (Figure S5). However, AC-specific genes increase their expression within cortical regions and exhibit decreased expression in basal ganglia (BG) upon aging (Figure 4B); therefore, their absolute expression signals become more similar across regions in the aging brain (Figure S5A). SNIG and THAL, which show the highest expression of AC-specific genes in the young brain, have a generic decrease of AC-specific genes upon aging (Figure 4B). In contrast, the AC-specific genes with the lowest expression in the young group increase their expression upon aging in all regions except SNIG and THAL. This leads to remarkable shifts in the regional patterns of AC-specific gene expression. Although expression of AC-specific genes clustered most brain regions separately for the young group, only four regional clusters remained in the old group, two of which were the CRBL and the cortical regions (Figure 4B). The most pronounced change is seen for HIPP and SNIG. For example, HIPP clusters close to cortex in the young group but shifts toward the WHMT and PUTM in the samples from the old group (Figure 4B).

The aging-altered genes that are specific for all stages of OLG differentiation, including OLG precursors, newly formed OLGs, and myelinating OLG generally show a trend toward decreased expression in all regions upon aging (Figures S5A–S5C). Moreover, OLG-specific genes show a shift of region-specific gene expression upon aging, with the strongest change of regional identity seen in HIPP and SNIG. In the samples from the young group, HIPP clusters close to cortical samples and SNIG clusters close to MEDU and THAL, whereas in the samples from the old group, HIPP and SNIG cluster closer to each other (Figure 4C).

Similar to the OLG-specific genes, the aging-altered neuron-specific genes showed predominant downregulation in all brain regions upon aging (Figure D; Figure S5B), in agreement with previous studies that observed decreased expression of neuron-specific genes in the cortex (Erraji-Benchekroun et al., 2005), but with the added insight that this occurs in a brain-wide manner. Classification based on aging-altered neuron-specific genes yielded a striking separation of samples based on their regional identity (Figure 4D), and the old group samples remained clustered closest to the young samples of the same brain region (Figure 4D). Thus, neuron-specific gene expression is more defined by regional identity than by age. This agrees with the finding that the downregulated genes, which are often neuron specific, are also generally region-specific, while the upregulated genes, which are often MG specific, are generally multi-regional (Figure 2D; Figure S5B).

Specific Neuronal Subpopulations and Oligodendrocytes Are Decreased in the Aging Brain

To examine how gene expression changes may relate to changes in brain cell populations, we developed an efficient pipeline for analysis of high-resolution image tiles of immunolabeled sections of FCTX. We developed a targeted computational pipeline for detection and quantification of the stained cells based on the scanned images, which consists of big data detection, segmentation, and quantification pipeline using thresholding, filtering, and object detection.

Noting the trend for decreased expression of OLG-specific genes in the frontal cortex (Figure 4D), we examined serial sections immunolabeled with OLIG2 antibody from the tissue blocks from the same brain samples that were used for the microarray study. We selected three young and three old cases based on their microarray profiles, such that it was representative for their age (see Supplemental Experimental Procedures). Approximately 50,000 image tiles were analyzed from the three young and three old FCTX sections (Figure 5A). We counted the number of OLIG2-positive cells compared to the total number of cell nuclei in each tile. Statistics was calculated on two classes of tile density, likely corresponding to local variations in the proportion of white matter (low density of nuclei) and gray matter (high density of nuclei), in addition to all densities combined (all tiles). The number of OLIG2-positive cells decreased in all classes of tiles in the aging FCTX, with the largest decrease in the low-density tiles in old compared to young cases (Figure 5C, middle panel). In contrast, the number of other cells significantly increased low-density tiles (Figure 5C, lower panel), in agreement with the slight increase in the total number of cells in the same tiles (Figure 5C, upper panel). This analysis demonstrates that the decreased expression of OLG-specific genes might partly reflect a decrease in three cortical OLG cell population.

The aging RNA expression signatures also revealed downregulation of neuron-specific genes (Figure 4B); therefore, we analyzed high-resolution images produced from the three young and three old FCTX sections stained with NeuN antibody to mark the neurons. This antibody detects the neuron-specific RNA-binding protein RBFOX3, which is predominantly nuclear, but is also present in the cytoplasm of the cell body (Kim et al., 2009) (Figure 6A). We used the tissue samples from the same cases as were used for OLIG2 quantification, thus allowing direct comparison of the two cell types. To capture the large diversity of both shape and size of cell bodies in the neuronal populations, we used a large tile size (10,000 × 10,000 pixels each) (Figure 6Bi). This allowed us to extract information from almost all layers of the neocortex in each slide of gray matter. A preliminary quality control analysis flagged one image from a young individual as a technical outlier, and this sample was therefore omitted from further analysis, although we provide access to its data (https://figshare.com/s/f2675361af1242f3565f). We processed 1,044 image tiles using our cell detection pipeline and applied an information exclusion criterion (entropy > 5) to contain the most meaningful slides (n = 641). In an attempt to enrich the regions of gray matter with the highest information content, we further focused on the 184 tiles with the highest density of nuclei. A total of 371,096 neurons were identified. We further segregated cells into four bins of total area of cell

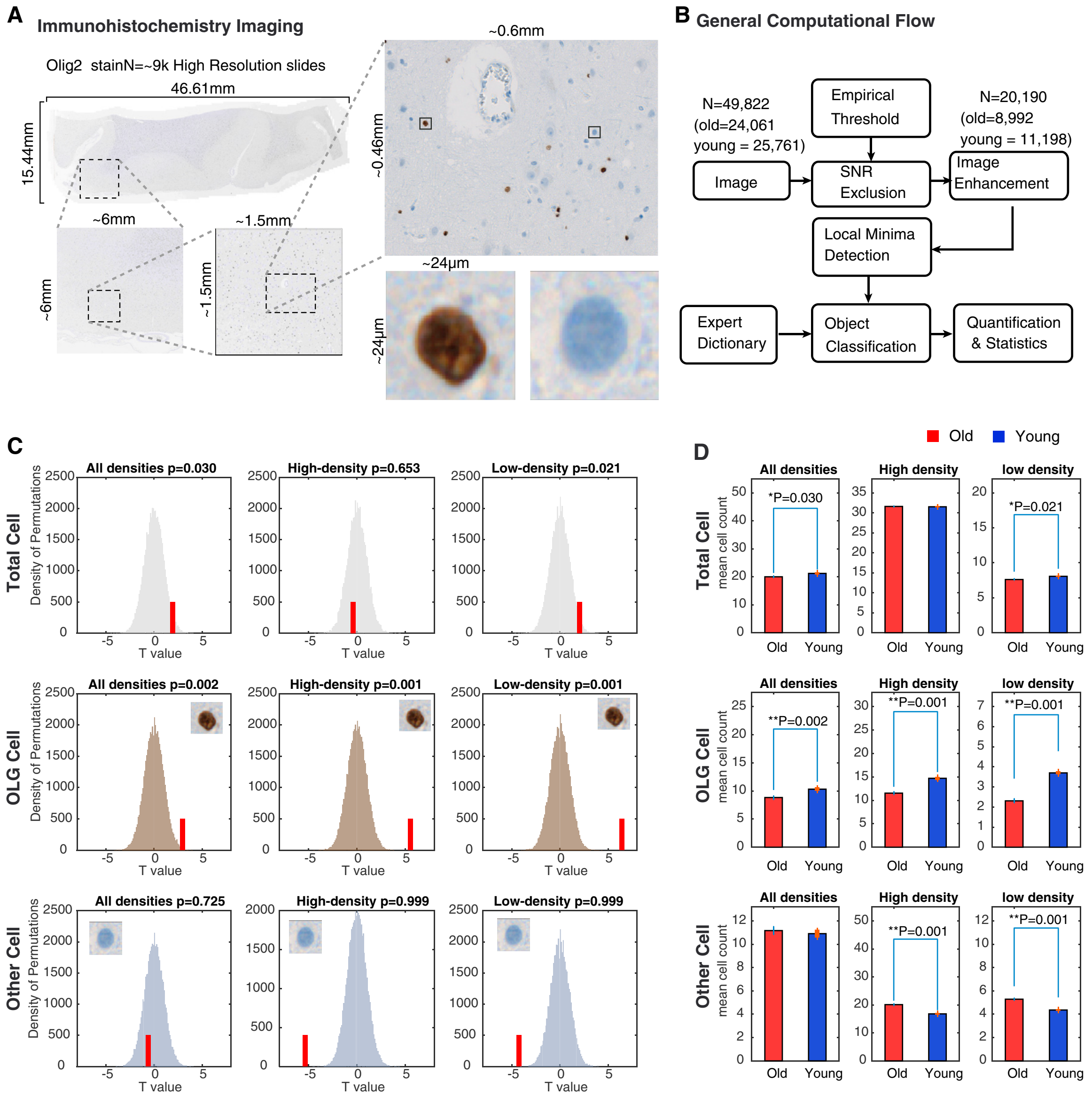

Figure 5. Decreased Counts of Oligodendrocytes in the Frontal Cortex upon Aging

Six FCTX brain sections were stained and imaged (from three old and three young post-mortem brain samples). Each sample contains thousands of equal-size slides each 1,600 × 1,200 pixels, as captured by a Zeiss AxioScan slide scanner following staining with the Olig2 antibody.

(A) An example of a BA9-Olig2 slide shown in a full-resolution pyramid, with gradual zooming into two typical cells: one stained brown (OLG cells) and one stained blue (other cells).

(B) General computational pipeline for the analysis of high-resolution immunohistochemical high-dimensional imaging data allowed us to quantify both OLG and other cells in each FCTX slide.

(C) Comparison of OLG counts that asks if the number of cells of interest is different in young samples compared to old (i.e., red bar shifted to the right means increased count in young samples). In each panel, the histogram represents the null distribution of t values calculated using two-tailed Student's t test over slide cell counts randomly sampled from the entire population of the six samples, using 100 random iterations over 500 permutations where the true-label t statistics is depicted with a red bar, and the remaining distribution was calculated based on shuffled labels. The analysis was done on overall 8,766 young and 10,922 old group slides (left). From a total of 2,612 young and 1,828 old group high-density slides, the 50 slides with the highest density were selected per case for quantification. Similarly, from 1,154 young and 1,277 old group low-density slides, the 50 slides with the lowest density were chosen per sample for quantification.

(D) Cell counts in samples from old (red) and young (blue) groups, with significance calculated with t statistics as described in (C). The star marks bars with a p value < 0.05 and the mean T statistic, p value and SD of the permutation test are reported on top of the graphs.

body (small: 500–3,000 pixels, n = 92,947; medium: 3,000–6,000 pixels, n = 202,239; large: 6,000–9,000 pixels, n = 60,314; very large: >9,000 pixels, n = 15,596). In agreement with the previous study (Kim et al., 2009), the intensity of cytoplasmic NeuN signal was strongest in the largest cells (Figure 6C). To account for the imbalance of tiles across samples, significance in the

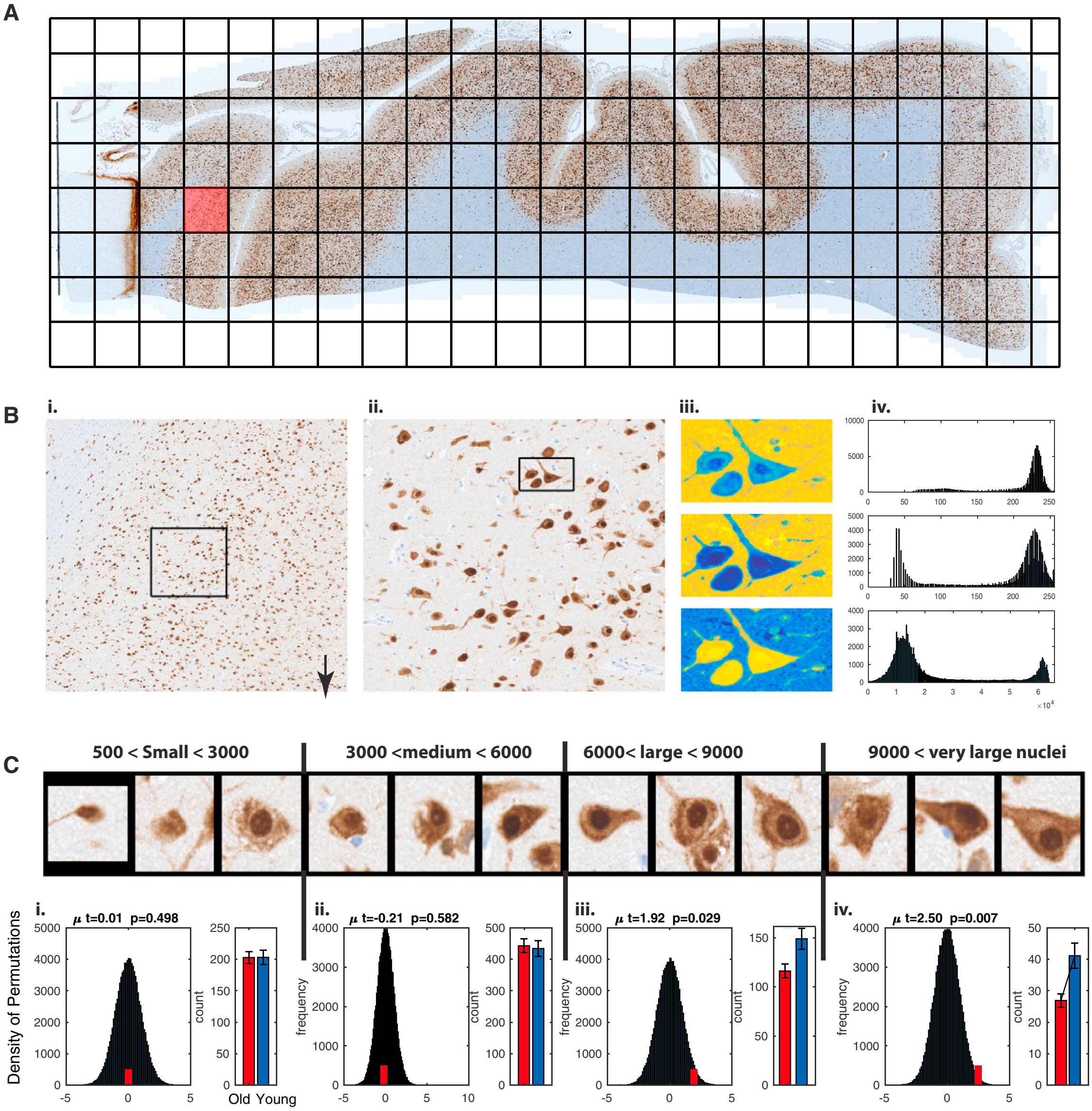

Figure 6. Decreased Counts of Specific Neuronal Populations in the Frontal Cortex upon Aging

(A) An image of one NeuN-stained FCTX section, with re-defined tiles demonstrated by black rectangles (file size = 37.4 GB).

(B) (i) Enlargement of a single tile of 10,000 × 10,000 pixels (size = 225 MB). (ii) Enlargement of a 2,500 × 2,500 pixel section. (iii) Three cells as observed in the red channel (top, shown in light blue) and blue channel (middle), and intersection of the two channels (bottom) differentiates between neuronal cells (stained by NeuN in brown on the original slides) and other cells (stained by Heamotoxylin in blue in the middle plot). (iv) The x axis represents the color frequency distribution of the red and blue channels across an intensity range of 256 gray levels, while the y axis represents the frequency of pixel intensity in the image tile depicted in (iii).

(C) Examples of detected neurons that contain small, medium, medium to large (E), or large cell body (F) with size given in pixels. Underneath each image is the histogram that asks if the number of cells of interest is different in young samples compared to old (i.e., red bar shifted to the right means increased count in young samples). The histogram shows the null distribution of t values, calculated using two-tailed Student's t test over slide counts using 100,000 random permutations from the entire population of the six samples (black bars), while the mean of the true-label t statistics is depicted with a red bar. The right graph shows the cell counts in samples from old (red) and young (blue) groups, with significance calculated with t statistics based on 10,000 random permutations. The star marks bars with a p value < 0.05, and the mean t statistics, p value, and SD of the permutation test are reported on top of the graphs.

age-dependent decrease in each neuronal population was tested with right-tailed two-sample t test. We observed no change in the number of neurons with small or medium-size cell bodies, which represent 80% of detected neurons. In contrast, the number of neurons with large or very large cell bodies is significantly decreased ($p = 0.029$ and $p = 0.007$, accordingly; right-tailed two-sample t test) Figure 6C).

Altogether, it is likely that changes in gene expression observed in our study reflect a combination of changes in expression profiles and changes in quantity of specific cell types.

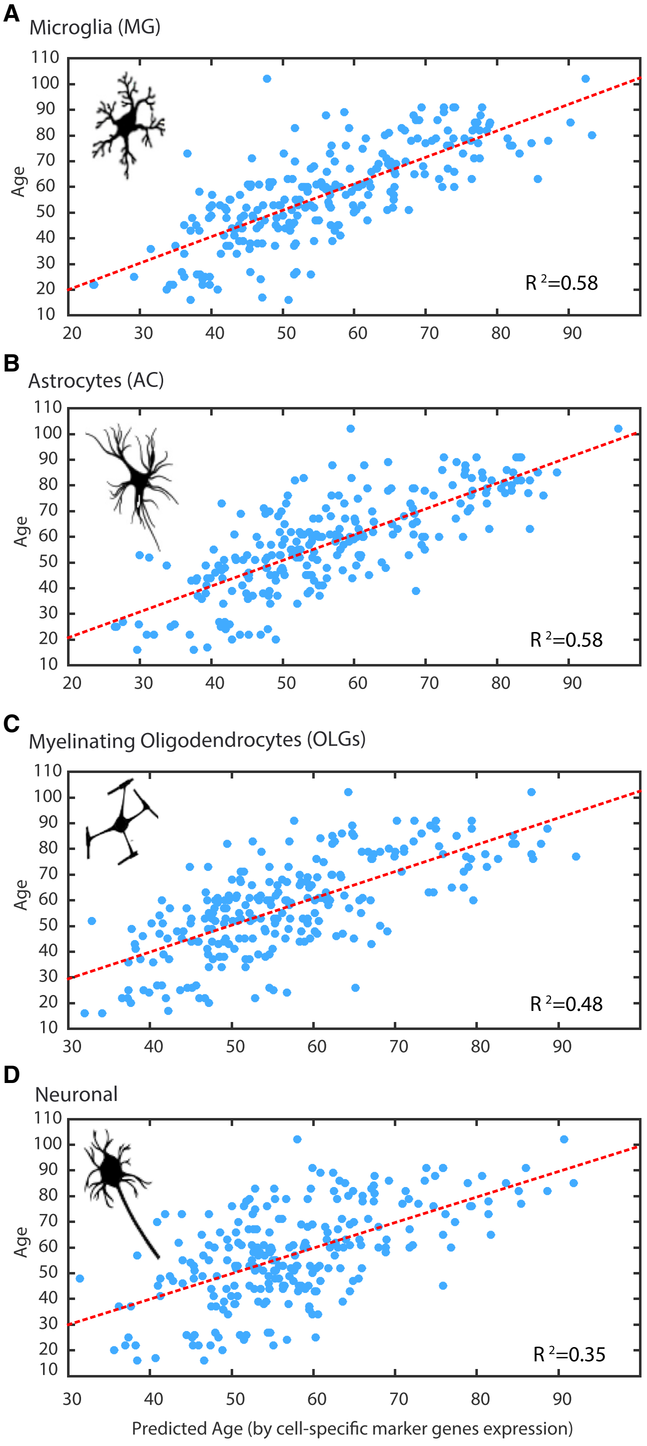

Figure 7. Glial-Specific Genes Are Most Capable of Predicting Biological Age

(A–D) Analysis of the accuracy of cell-type-specific genes in predicting the biological age of UKBEC brain samples: (A) MG-specific genes (R^2 = 0.58), (B) AC-specific genes (R^2 = 0.58), (C) neuron-specific genes (R^2 = 0.35), (D) OLG precursor-specific genes (R^2 = 0.48).

In all plots, the y axis denotes the actual age and the x axis denotes the predicted age. MG, microglia; AC, astrocytes; OLG, oligodendrocytes. See Figure S4C for age association plots of endothelial, OLG precursor, and newly formed OLG cell-specific genes.

While most neuron-specific genes are predominantly downregulated, the extent of this downregulation varies among brain regions. All other cell types have a more complex pattern of changes. These changes appear most pronounced in HIPP and SNIG, which show the strongest shifts in the regional expression pattern of AC- and OLG-specific genes upon aging.

MG- and Endothelial-Specific Genes Are the Best Predictors of Biological Age

Given our aforementioned findings, we next sought to gain insight into whether glial genes can predict age category with more fidelity than neurons and sought to understand the precise nature of gene expression changes driving this. Having established cross-regional and cell-type-specific gene expression relationships upon aging, we next asked which cell-type expression patterns within the brain are most associated with age. We applied a stepwise regression to construct an age-associative model based on the expression signals of cell-type-specific genes to compare them (Figure 7; Figure S4; Supplemental Experimental Procedures). We accounted for both the brain bank source and the cause of death. Application of the model uncovered defined groups of the highest age-relevant genes for each cell type. In a few cases, interaction between two genes was found to be age predictive (Table S3). Several multi-regional genes were also detected as age-predictive cell-type-specific genes, including *CP*, *SGPP1*, and *VWF*, which were detected as OLG or endothelial specific (Table S4). MG-, AC-, and endothelial-specific genes were most highly associated with biological age, while the smallest number of age-predictive genes was found among the neuron-specific genes (Figure 6). Altogether, our data implicate expression of glial-specific genes, rather than neuronal-specific genes, as the most reliable predictor of biological age in the human brain.

Functional Enrichment Analysis of Multi-regional and Region-Specific Aging-Altered Genes

To gain further insight into the functional nature of aging-altered genes, we performed enrichment analysis of Gene Ontology (GO) terms of these genes (Edgar et al., 2013). The upregulated multi-regional genes were enriched in the following functional terms: "MG cell development", "interleukin-1 (IL-1) receptor activity", and "immune response". Supporting these observations, neuroinflammation is known to be involved in aging, with evidence implicating the interferon type I response in aging-associated cognitive decline (Baruch et al., 2014). Conversely, downregulated multi-regional genes were enriched in the processes of "protein transport and localization", and aging-altered expression of these genes led to shifts in regional identity (Figure S2B). Moreover, 244 of a total of 253 genes annotated to the "protein transport" category were detected as altered upon aging in at least one brain region. These genes separated the CRBL from the other regions and maintained their regional sub-classification (Figure S5Bi). In addition, the CRBL samples of the old group remained clearly separated from the rest

(Figure S5Bii). Most aging-altered genes annotated to this category were downregulated in eight regions, apart from the CRBL and WHMT, which showed greater expression variability (Figure S5Biii). The WHMT aging-altered genes were functionally enriched in "regulation of cell adhesion", "regulation of cell development", "metabolic processes", and "cognition" (Figure S6A). Conversely, among the top functional terms that were enriched in aging-altered genes in the FCTX were immune functions including "T cell differentiation", "T cells", and "leukocyte and lymphocyte activation" (Table S2). Among the CRBL-enriched functions were "cell adhesion", "regulation of cell motion and migration", and "neuron projection morphogenesis". These results imply a region-specific functional heterogeneity of the brain aging process.

Analysis of the cell-type-specific aging-altered genes revealed enrichment of further functional pathways. "Synaptosome", "regulation of programmed cell death", and "metal ion transporter" were enriched in downregulated neuron-specific genes. "Regulation of adaptive immune response", "natural killer cell-mediated cytotoxicity", and "cell adhesion and motion" were enriched in MG-specific upregulated genes (Table S2). "Myelination", "oxidoreductase", and "RAS protein signal transduction" were enriched in upregulated OLG-specific genes. "Mitochondrial matrix", "phosphate metabolic process", and "Kyoto Encyclopedia of Genes and Genomes (KEGG) pathway AD" were enriched in downregulated myelinating OLG-specific genes. Finally, "cell morphogenesis" and "cell-cell adhesion" were enriched in upregulated AC-specific genes. Some of these functions have also been identified in a study that examined the initial cell-type-specific transcriptional changes in a mouse model of amyotrophic lateral sclerosis (ALS), including synaptic functions in neurons and membrane signaling defects in OLGs (Sun et al., 2015).

DISCUSSION

This study presents a comprehensive analysis of RNA expression in ten regions of the human brain and large-scale cell quantification in FCTX upon aging. Our findings show that cell-type-specific genes delineate samples based on both age group and brain region. Aging was the major determinant of glia-specific gene expression shifts in regional identity, while such changes were not evident in neuron-specific genes. Genes specific for neurons and OLGs generally decreased their expression upon aging, while MG-specific genes increased their expression profiles, consistent with the known MG activation in aging (Norden and Godbout, 2013). ACs showed a more complex pattern of reciprocal regional changes upon aging, with upregulation in the cortical regions and downregulation in the deeper brain structures. Among the genes specific for the non-neuronal cell types, those with the highest absolute expression in the young group decrease their expression upon aging in most brain regions, while those with the lowest expression in the young group increase their expression in a subset of regions. This leads to major shifts in region-specific gene expression, particularly of AC- and OLG-specific genes, which are most pronounced in the HIPP and SNIG, the regions that are archetypally affected in the most common age-related neurodegenerative diseases (AD and PD, respectively). These findings reinforce a growing body of evidence implicating glia in aging (Norden and Godbout, 2013).

Age-related degeneration of OLG has been previously observed in the HIPP of the senescence-accelerated mice, as well as other animal models (Hayakawa et al., 2007; Hwang et al., 2006; Shimeda et al., 2005). OLG-specific genes were also found to have the strongest enrichment among genes with decreased age-related expression in human TCTX (Tollervey et al., 2011). We demonstrate that the age-related downregulation of OLG-specific gene expression is accompanied by a decrease in OLG cell numbers in the FCTX, consistent with previous observations of decreased OLGs in neocortical regions of old human brains (Fabricius et al., 2013; Pelvig et al., 2008). The OLG-specific aging-altered genes include *MBP*, a major constituent of the myelin sheath, and *LINGO1*, a regulator of myelination (Mi et al., 2005). It is of interest that the low-density tiles (corresponding to likely white matter) show the largest decrease in oligodendrocytes and a corresponding increase in other cells (Figure 5). Given the strongest upregulation of MG-specific genes in the brain, it appears possible that the increase of other cells is driven by the increase in MG, but this remains to be directly examined.

We found increased AC-specific gene expression in human aging HIPP, which agrees with data from aging mouse models, in which increased proliferation and activation of ACs are reported (Hayakawa et al., 2007) (Figure 4). An examination of three sub-regions of mouse HIPP using three AC-specific protein markers revealed complex, region-specific, and marker-dependent changes (Rodríguez et al., 2014). Regionally encoded AC expression is important for neuronal functions, as was demonstrated by the loss of ventral spinal cord AC-encoded *SEMA3A* gene expression, which leads to selective death of α-motor neurons in mice (Molofsky et al., 2014). We find many regional differences in expression of AC-specific genes are largely erased in samples from the old group; for example, these genes cluster the HIPP and PUTM separately in the young group, but not in the old group (Figure 4B). This suggests that major changes in functional heterogeneity of AC take place in the aging brain, which might have deleterious consequences on the integrity of neuronal circuits.

A trend toward increased expression of MG-specific genes was observed in all regions upon aging, with corresponding upregulation of genes with immune or inflammatory functions. The upregulated genes include *C1Q*, which agrees with the increased C1Q protein levels that were observed in both mouse and human brains upon aging (Stephan et al., 2013). Another upregulated gene is *TREM2*, which is also upregulated in amyloid-plaque-associated MG (Frank et al., 2008) and contains variant alleles that increase AD risk (Guerreiro et al., 2013). Upregulation of inflammatory functions is in line with evidence implicating the interferon type I response in age-associated cognitive decline (Baruch et al., 2014).

In addition to glial changes, we also observed a decreased number of neurons with large cell bodies, which represent approximately 20% of neurons in the cortex. Although we did not attempt to directly identify the neuronal subtypes in the present study, neurons with the largest cell bodies are likely to be

associative pyramidal neurons (Zeba et al., 2008). Furthermore, these neurons were previously indicated to be most vulnerable to aging in a study of Rhesus monkeys (Gilman et al., 2016). While our analysis indicates that the decrease in these pyramidal neurons may be the primary source of the downregulation of neuron-specific genes, our findings regarding the cortical neuronal cells remain speculative due to the limited number of individuals used for the imaging analyses. Moreover, it remains possible that the change does not result from loss of these neurons, but rather from downregulation of Rbfox3 protein, or its loss from the cytoplasm of large neurons. Thus, our current analysis will need to be verified with the use of additional markers of specific neuronal cell types and increased sample size, which will potentially include additional brain regions; ideally, it will also be compared to the outcomes of cell-type-specific analyses of RNA sequencing datasets (Lake et al., 2016).

Age is the major risk factor for both AD and PD, the two most prevalent neurodegenerative diseases. It is becoming clear that the pre-clinical stage of AD begins decades before clinical manifestation (Dubois et al., 2014). This pre-clinical stage has been termed "the cellular phase," because it involves changes in interactions among all cell types in the brain, with the most dramatic changes taking place in AC, MG, and vasculature (De Strooper and Karran, 2016). We find a corrosion of glial region-specific gene expression in aging, with the genes specific for AC, MG, and endothelial cells being the best predictors of age. HIPP and SNIG are affected in the early stages of AD and PD, respectively, and these are the two regions with major shifts in their regional expression profiles of AC- and OLG-specific genes upon aging. Thus, our data may provide insights into the role of glia in the region-specific vulnerability in these age-related neurodegenerative diseases.

By simultaneously assessing changes in cell-type-specific genes across multiple brain areas, our study takes a step toward providing a comprehensive framework of the molecular and cellular changes in human aging. While our primary aim was to deconvolute the cell-type-specific signatures present within large databases of age-related transcriptional changes, we also made a step toward interpreting these in light of changes in counts of OLG and neuronal cells. Integration of further genome-wide and single-cell data from human tissues samples and cell and animal models will be required to fully understand the cellular and molecular mechanisms underlying the observations in our study. Altogether, our study indicates that the cellular changes during aging involve a dramatic shift in the regional identity of glia, and it provides a resource for further studies of the relationship between aging and the cellular phase of dementia.

Conclusions

Our study examines brain-wide gene expression patterns in the aging human brain across a wide physiological age range, coupled with complementary analysis of cell-type-specific marker genes and validation by direct cell quantification using immunohistochemical imaging followed by targeted computational analysis. In addition to the expected increase in expression of MG-specific genes and decrease in expression of neuron-specific genes, our analyses uncovered major changes in the region-specific expression of AC- and OLG-specific genes. The age-associated changes in the regional expression of glial-specific genes are most dramatic in HIPP and SNIG, the brain regions affected in AD and PD. The age-dependent decrease in expression of OLG- and neuron-specific genes aligns with the results of direct cortical cell counting, in which decreased numbers of OLGs and of neurons with large cell bodies are demonstrated. We believe that our data and computational approaches provide a powerful resource for further study of the cellular and molecular changes taking place during human brain aging and provide insights into the pre-clinical cellular phase of dementia.

EXPERIMENTAL PROCEDURES

Ethical Statement

All samples used for this study had fully informed consent for retrieval and were authorized for ethically approved scientific investigation (Research Ethics Committee number 10/H0716/3).

Brain Samples

Post-mortem human brain material was produced under institutional guidelines governed by approved protocols. Tissue samples were produced from 99 individuals by the Sudden Death MRC brain bank, 35 individuals by the Sun Head Institute for the UKBEC, and 305 individuals by the American Brain Bank (NABEC).

Quality Assessment and Array Pre-processing

For the UKBEC dataset, all quality measurements were extensively described in a previous publication (Trabzuni et al., 2011). The initial pre-processing of the microarray data, including application of RMA (robust multi-assay) average quantile normalization with guanine cytosine (GC) background corrections (GC-RMA) and expression data were log2 transformed. The gene level signal estimates were calculated for a total of 26,493 transcripts using the median signal of each group of probe sets interrogating a transcript.

Expression Data Analysis

A tailored analysis pipeline was developed for all computational analyses and data visualization of microarray and RNA-seq datasets that were analyzed in this study (in MATLAB, R2014-2016a). Those include construction of data structures and statistical significance inference using ANOVA, with false discovery rate (FDR) thresholding (of corrected $p < 1e-3$), classification and clustering (e.g., using t-distributed stochastic neighbor embedding [t-SNE] and hierarchical clustering), and data visualization.

Cell-type-specific genes were defined by analysis of RNA-seq data from mouse brain (http://web.stanford.edu/group/barres_lab/brain_rnaseq.html) and were further used to find age-predictive cell-specific genes. The lists are under Tables S5 and S6, accordingly. Additional cell-specific lists were based on a previous microarray data on three of these cell types (Cahoy et al., 2008). Further details are in the Supplemental Information.

High-Resolution Imaging and Analysis of Immunolabeled Brain Samples

Post-mortem human brain sections were placed into xylene and rehydrated. Antigen retrieval was performed with citric acid. For OLG staining, the samples were immunolabeled with OLIG2 antibody using the Leica Novolink Polymer detection kit. We used the Olig2 antibody from Millipore (catalog #AB9610) at 1/200 dilution. For staining of neurons, the samples were immunolabeled with NeuN antibody (Acris) and the Leica Bond Epitope Retrieval Solution#1 was used (AR9961 from Leica Biosystems) (AR9961 from Leica Biosystems). The images for both types of stains were acquired on the Zeiss AxioScan slide scanner. Details of the cell detection and quantification computational methods for neurons are given under the Supplemental Experimental Procedures. In addition, all raw jpeg images of the slides can be seen at https://figshare.com/s/f2675361af1242f3565f. For image analysis, we employed

some of the computational methods mentioned by Bjornsson et al. (2008), in addition to a targeted computational pipeline developed in-house in MATLAB (see details under Supplemental Experimental Procedures).

ACCESSION NUMBERS

The accession numbers for the UKBEC exon and NABEC expression datasets reported in this paper are GEO: GSE46706 and GSE36192, respectively.

SUPPLEMENTAL INFORMATION

Supplemental Information includes Supplemental Experimental Procedures, seven figures, and seven tables and can be found with this article online at http://dx.doi.org/10.1016/j.celrep.2016.12.011.

CONSORTIA

The members of the UK Brain Expression Consortium are John Hardy, Mina Ryten, Daniah Trabzuni, Sebastian Guelfi, Michael E. Weale, Adaikalavan Ramasamy, Paola Forabosco, Colin Smith, and Robert Walker. The members of the North American Brain Expression Consortium are Sampath Arepalli, Mark R. Cookson, Allissa Dillman, J. Raphael Gibbs, Dena G. Hernandez, Michael A. Nalls, Andrew B. Singleton, Bryan Traynor, Marcel van der Brug, Luigi Ferrucci, Robert Johsnon, Ronal Zielke, Dan L. Longo, Juan Toncoso, and Alan Zonderman.

AUTHOR CONTRIBUTIONS

L.S., R.P., and J.U. conceived and designed the project and wrote the manuscript with contributions from all co-authors; J.U. and R.P. contributed equally to the work. L.S. developed the computational pipelines, analyzed the data, and produced the figures. E.S. developed targeted imaging computational analyses. M.R. and D.T. provided RNA extraction and microarray sample preparations. J.H. and M.R. provided access to the UKBEC dataset, and M.R.C. provided access to the NABEC datasets. J.R. and C.S. provided immunohistochemistry preparation, and R.P. supervised the immunohistochemical slide analysis and interpretation.

ACKNOWLEDGMENTS

This work was supported by the European Research Council (617837-Translate) to J.U.; the Marie Curie Intra European Fellowship (330430-PRANA), and the Alzheimer's Society for Junior Investigator award (award 172065, project 534121) to L.S.; the Francis Crick Institute, which receives its core funding from Cancer Research UK (FC001002), the UK Medical Research Council (FC001002); the Wellcome Trust (FC001002); the UK Medical Research Council (MRC) through the MRC Sudden Death Brain Bank to C.S.; a project grant (G0901254) to J.H. and a training fellowship (G0802462) to M.R.; and in part by the Intramural Research Program of the US National Institute on Aging, NIH, Department of Health and Human Services (project ZO1 AG000947). R.P. is a Wellcome Trust Intermediate Clinical Fellow (101149/Z/13/A) and an Anne Rowling Fellow in Regenerative Neurology. D.T. was supported by the King Faisal Specialist Hospital and Research Centre, Saudi Arabia. We are grateful to the Banner Sun Health Research Institute Brain and Body Donation Program of Sun City, Arizona, for the provision of human biospecimens. The Brain and Body Donation Program is supported by the US National Institute of Neurological Disorders and Stroke (U24 NS072026 to the National Brain and Tissue Resource for Parkinson's Disease and Related Disorders), the National Institute on Aging (P30 AG19610 to the Arizona Alzheimer's Disease Core Center), the Arizona Department of Health Services (contract 211002 to the Arizona Alzheimer's Research Center), the Arizona Biomedical Research Commission (contracts 4001, 0011, 05-901, and 1001 to the Arizona Parkinson's Disease Consortium), and the Michael J. Fox Foundation for Parkinson's Research. This research was supported in part by the Intramural Research Program of the NIH, National Institute on Aging.

Received: May 2, 2016
Revised: October 4, 2016
Accepted: December 2, 2016
Published: January 10, 2017

REFERENCES

Baruch, K., Deczkowska, A., David, E., Castellano, J.M., Miller, O., Kertser, A., Berkutzki, T., Barnett-Itzhaki, Z., Bezalel, D., Wyss-Coray, T., et al. (2014). Aging. Aging-induced type I interferon response at the choroid plexus negatively affects brain function. Science *346*, 89–93.

Bjornsson, C.S., Lin, G., Al-Kofahi, Y., Narayanaswamy, A., Smith, K.L., Shain, W., and Roysam, B. (2008). Associative image analysis: a method for automated quantification of 3D multi-parameter images of brain tissue. J. Neurosci. Methods *170*, 165–178.

Cahoy, J.D., Emery, B., Kaushal, A., Foo, L.C., Zamanian, J.L., Christopherson, K.S., Xing, Y., Lubischer, J.L., Krieg, P.A., Krupenko, S.A., et al. (2008). A transcriptome database for astrocytes, neurons, and oligodendrocytes: a new resource for understanding brain development and function. J. Neurosci. *28*, 264–278.

Cerbai, F., Lana, D., Nosi, D., Petkova-Kirova, P., Zecchi, S., Brothers, H.M., Wenk, G.L., and Giovannini, M.G. (2012). The neuron-astrocyte-microglia triad in normal brain ageing and in a model of neuroinflammation in the rat hippocampus. PLoS ONE *7*, e45250.

Cribbs, D.H., Berchtold, N.C., Perreau, V., Coleman, P.D., Rogers, J., Tenner, A.J., and Cotman, C.W. (2012). Extensive innate immune gene activation accompanies brain aging, increasing vulnerability to cognitive decline and neurodegeneration: a microarray study. J. Neuroinflammation *9*, 179.

De Strooper, B., and Karran, E. (2016). The cellular phase of Alzheimer's disease. Cell *164*, 603–615.

Dubois, B., Feldman, H.H., Jacova, C., Hampel, H., Molinuevo, J.L., Blennow, K., DeKosky, S.T., Gauthier, S., Selkoe, D., Bateman, R., et al. (2014). Advancing research diagnostic criteria for Alzheimer's disease: the IWG-2 criteria. Lancet Neurol. *13*, 614–629.

Edgar, R., Mazor, Y., Rinon, A., Blumenthal, J., Golan, Y., Buzhor, E., Livnat, I., Ben-Ari, S., Lieder, I., Shitrit, A., et al. (2013). LifeMap Discovery™: the embryonic development, stem cells, and regenerative medicine research portal. PLoS ONE *8*, e66629.

Erraji-Benchekroun, L., Underwood, M.D., Arango, V., Galfalvy, H., Pavlidis, P., Smyrniotopoulos, P., Mann, J.J., and Sibille, E. (2005). Molecular aging in human prefrontal cortex is selective and continuous throughout adult life. Biol. Psychiatry *57*, 549–558.

Fabricius, K., Jacobsen, J.S., and Pakkenberg, B. (2013). Effect of age on neocortical brain cells in 90+ year old human females—a cell counting study. Neurobiol. Aging *34*, 91–99.

Frank, S., Burbach, G.J., Bonin, M., Walter, M., Streit, W., Bechmann, I., and Deller, T. (2008). TREM2 is upregulated in amyloid plaque-associated microglia in aged APP23 transgenic mice. Glia *56*, 1438–1447.

Gibbs, J.R., van der Brug, M.P., Hernandez, D.G., Traynor, B.J., Nalls, M.A., Lai, S.L., Arepalli, S., Dillman, A., Rafferty, I.P., Troncoso, J., et al. (2010). Abundant quantitative trait loci exist for DNA methylation and gene expression in human brain. PLoS Genet. *6*, e1000952.

Gilman, J.P., Medalla, M., and Luebke, J.I. (2016). Area-specific features of pyramidal neurons—a comparative study in mouse and rhesus monkey. Cereb. Cortex, bhw062.

Grabert, K., Michoel, T., Karavolos, M.H., Clohisey, S., Baillie, J.K., Stevens, M.P., Freeman, T.C., Summers, K.M., and McColl, B.W. (2016). Microglial brain region-dependent diversity and selective regional sensitivities to aging. Nat. Neurosci. *19*, 504–516.

Guerreiro, R., Wojtas, A., Bras, J., Carrasquillo, M., Rogaeva, E., Majounie, E., Cruchaga, C., Sassi, C., Kauwe, J.S., Younkin, S., et al.; Alzheimer Genetic Analysis Group (2013). TREM2 variants in Alzheimer's disease. N. Engl. J. Med. *368*, 117–127.

Harel, A., Inger, A., Stelzer, G., Strichman-Almashanu, L., Dalah, I., Safran, M., and Lancet, D. (2009). GIFtS: annotation landscape analysis with GeneCards. BMC Bioinformatics *10*, 348.

Hayakawa, N., Kato, H., and Araki, T. (2007). Age-related changes of astrocytes, oligodendrocytes and microglia in the mouse hippocampal CA1 sector. Mech. Ageing Dev. *128*, 311–316.

Head, D., Buckner, R.L., Shimony, J.S., Williams, L.E., Akbudak, E., Conturo, T.E., McAvoy, M., Morris, J.C., and Snyder, A.Z. (2004). Differential vulnerability of anterior white matter in nondemented aging with minimal acceleration in dementia of the Alzheimer type: evidence from diffusion tensor imaging. Cereb. Cortex *14*, 410–423.

Hwang, I.K., Yoo, K.Y., Kim, D.S., Kang, T.C., Lee, B.H., Kim, Y.S., and Won, M.H. (2006). Chronological distribution of Rip immunoreactivity in the gerbil hippocampus during normal aging. Neurochem. Res. *31*, 1119–1125.

Kim, K.K., Adelstein, R.S., and Kawamoto, S. (2009). Identification of neuronal nuclei (NeuN) as Fox-3, a new member of the Fox-1 gene family of splicing factors. J Biol Chem. *284*, 31052–31061.

Kumar, A., Gibbs, J.R., Beilina, A., Dillman, A., Kumaran, R., Trabzuni, D., Ryten, M., Walker, R., Smith, C., Traynor, B.J., et al. (2013). Age-associated changes in gene expression in human brain and isolated neurons. Neurobiol. Aging *34*, 1199–1209.

Lake, B.B., Ai, R., Kaeser, G.E., Salathia, N.S., Yung, Y.C., Liu, R., Wildberg, A., Gao, D., Fung, H.L., Chen, S., et al. (2016). Neuronal subtypes and diversity revealed by single-nucleus RNA sequencing of the human brain. Science *352*, 1586–1590.

Lu, T., Aron, L., Zullo, J., Pan, Y., Kim, H., Chen, Y., Yang, T.H., Kim, H.M., Drake, D., Liu, X.S., et al. (2014). REST and stress resistance in ageing and Alzheimer's disease. Nature *507*, 448–454.

Matarin, M., Salih, D.A., Yasvoina, M., Cummings, D.M., Guelfi, S., Liu, W., Nahaboo Solim, M.A., Moens, T.G., Paublete, R.M., Ali, S.S., et al. (2015). A genome-wide gene-expression analysis and database in transgenic mice during development of amyloid or tau pathology. Cell Rep. *10*, 633–644.

Mi, S., Miller, R.H., Lee, X., Scott, M.L., Shulag-Morskaya, S., Shao, Z., Chang, J., Thill, G., Levesque, M., Zhang, M., et al. (2005). LINGO-1 negatively regulates myelination by oligodendrocytes. Nat. Neurosci. *8*, 745–751.

Molofsky, A.V., Kelley, K.W., Tsai, H.H., Redmond, S.A., Chang, S.M., Madireddy, L., Chan, J.R., Baranzini, S.E., Ullian, E.M., and Rowitch, D.H. (2014). Astrocyte-encoded positional cues maintain sensorimotor circuit integrity. Nature *509*, 189–194.

Mosher, K.I., and Wyss-Coray, T. (2014). Microglial dysfunction in brain aging and Alzheimer's disease. Biochem. Pharmacol. *88*, 594–604.

Norden, D.M., and Godbout, J.P. (2013). Review: microglia of the aged brain: primed to be activated and resistant to regulation. Neuropathol. Appl. Neurobiol. *39*, 19–34.

Orre, M., Kamphuis, W., Dooves, S., Kooijman, L., Chan, E.T., Kirk, C.J., Dimayuga Smith, V., Koot, S., Mamber, C., Jansen, A.H., et al. (2013). Reactive glia show increased immunoproteasome activity in Alzheimer's disease. Brain *136*, 1415–1431.

Pelvig, D.P., Pakkenberg, H., Stark, A.K., and Pakkenberg, B. (2008). Neocortical glial cell numbers in human brains. Neurobiol. Aging *29*, 1754–1762.

Perry, V.H., and Teeling, J. (2013). Microglia and macrophages of the central nervous system: the contribution of microglia priming and systemic inflammation to chronic neurodegeneration. Semin. Immunopathol. *35*, 601–612.

Peters, A., and Sethares, C. (2004). Oligodendrocytes, their progenitors and other neuroglial cells in the aging primate cerebral cortex. Cereb. Cortex *14*, 995–1007.

Rodríguez, J.J., Yeh, C.Y., Terzieva, S., Olabarria, M., Kulijewicz-Nawrot, M., and Verkhratsky, A. (2014). Complex and region-specific changes in astroglial markers in the aging brain. Neurobiol. Aging *35*, 15–23.

Shimeda, Y., Hirotani, Y., Akimoto, Y., Shindou, K., Ijiri, Y., Nishihori, T., and Tanaka, K. (2005). Protective effects of capsaicin against cisplatin-induced nephrotoxicity in rats. Biol. Pharm. Bull. *28*, 1635–1638.

Sibille, E. (2013). Molecular aging of the brain, neuroplasticity, and vulnerability to depression and other brain-related disorders. Dialogues Clin. Neurosci. *15*, 53–65.

Stephan, A.H., Madison, D.V., Mateos, J.M., Fraser, D.A., Lovelett, E.A., Coutellier, L., Kim, L., Tsai, H.H., Huang, E.J., Rowitch, D.H., et al. (2013). A dramatic increase of C1q protein in the CNS during normal aging. J. Neurosci. *33*, 13460–13474.

Streit, W.J., and Xue, Q.S. (2010). The brain's aging immune system. Aging Dis. *1*, 254–261.

Sun, S., Sun, Y., Ling, S.C., Ferraiuolo, L., Mcalonis-Downes, M., Zou, Y., Drenner, K., Want, Y., Ditsworth, D., Tokunaga, S., et al. (2015). Translational profiling identifies a cascade of damage initiated in motor neurons and spreading to glia in mutant SOD1-mediated ALS. Proc. Natl. Acad. Sci. USA *112*, E6993–E7002.

Tollervey, J.R., Wang, Z., Hortobágyi, T., Witten, J.T., Zarnack, K., Kayikci, M., Clark, T.A., Schweitzer, A.C., Rot, G., Curk, T., et al. (2011). Analysis of alternative splicing associated with aging and neurodegeneration in the human brain. Genome Res. *21*, 1572–1582.

Trabzuni, D., Ryten, M., Walker, R., Smith, C., Imran, S., Ramasamy, A., Weale, M.E., and Hardy, J. (2011). Quality control parameters on a large dataset of regionally dissected human control brains for whole genome expression studies. J. Neurochem. *119*, 275–282.

Tremblay, M.E., Zettel, M.L., Ison, J.R., Allen, P.D., and Majewska, A.K. (2012). Effects of aging and sensory loss on glial cells in mouse visual and auditory cortices. Glia *60*, 541–558.

Vernooij, M.W., de Groot, M., van der Lugt, A., Ikram, M.A., Krestin, G.P., Hofman, A., Niessen, W.J., and Breteler, M.M. (2008). White matter atrophy and lesion formation explain the loss of structural integrity of white matter in aging. Neuroimage *43*, 470–477.

Zeba, M., Jovanov-Milosević, N., and Petanjek, Z. (2008). Quantitative analysis of basal dendritic tree of layer III pyramidal neurons in different areas of adult human frontal cortex. Coll. Antropol. *32* (*Suppl 1*), 161–169.

Zhang, Y., Chen, K., Sloan, S.A., Bennett, M.L., Scholze, A.R., O'Keeffe, S., Phatnani, H.P., Guarnieri, P., Caneda, C., Ruderisch, N., et al. (2014). An RNA-sequencing transcriptome and splicing database of glia, neurons, and vascular cells of the cerebral cortex. J. Neurosci. *34*, 11929–11947.

Cell Reports, Volume *18*

Supplemental Information

Major Shifts in Glial Regional Identity Are a Transcriptional Hallmark of Human Brain Aging

Lilach Soreq, UK Brain Expression Consortium, North American Brain Expression Consortium,, Jamie Rose, Eyal Soreq, John Hardy, Daniah Trabzuni, Mark R. Cookson, Colin Smith, Mina Ryten, Rickie Patani, and Jernej Ule

Table of Contents[1]

1. SUPPLEMENTARY EXPERIMENTAL PROCEDURES 3

STUDY DATA SETS 3

RNA EXPRESSION DATA SETS 3

THE UKBEC DATA SET 3

THE NABEC DATA SET 4

REST FCTX DATA SET 4

RNA ISOLATION 4

UK-BEC EXON ARRAYS: RNA EXTRACTION IS DESCRIBED UNDER TRABZUNI ET AL. (TRABZUNI ET AL., 2011). 5

NABEC 3' ARRAYS: THE RNA ISOLATION AND ARRAY PROCESSING AS WELL AS QUALITY MEASUREMENTS ARE REPORTED IN RAMASAMY ET AL. (E.G (RAMASAMY ET AL., 2013A), (HERNANDEZ ET AL., 2012)). 5

HIGH-RESOLUTION IMMUNOHISTOCHEMICAL BRAIN IMAGING DATASET PRODUCTION 5

IMAGE ACQUISITION OF THE HIGH-RESOLUTION FCTX OLIG2 STAINED IMAGES 6

NEUN STAINED SECTIONS IMAGE ACQUISITION 7

ANALYSIS METHODS 8

MICROARRAYS 8

CELL TYPE SPECIFIC GENES: DEFINITION AND CLASSIFICATION ANALYSES 9

3. HIGH RESOLUTION SCANNED IMMUNOHISTOCHEMICAL STAINED IMAGES COMPUTATIONAL QUANTIFICATION 10

OLIG2 STAINED BRAIN SECTIONS IMAGE ANALYSIS 10

CELL QUANTIFICATION AND CLASSIFICATION MODEL 10

PERMUTATION TEST ON THE OLIG2 STAINED SLIDES 11

PERMUTATION TESTS ON THE NEUN STAINED SLIDES 13

4. CELL TYPE MARKER GENES AND AGE-ASSOCIATION STATISTICAL ANALYSIS 13

[1] Soreq L. et al., Major shifts in glial regional identity are a transcriptional hallmark of human brain aging

5. SUPPLEMENTARY TABLES LIST AND LEGENDS 13

SUPPLEMENTARY TABLE 1| UKBEC COHORT SAMPLES DETAILS, RELATED TO FIGURE 1 13

SUPPLEMENTARY TABLE 2| FUNCTIONAL ENRICHMENT OF BRAIN AGING ALTERED GENES, RELATED TO FIGURE 2 14

SUPPLEMENTARY TABLE 3| CELL TYPE MARKER GENES ALTERED IN THE AGING BRAIN, RELATED TO FIGURE 4 14

SUPPLEMENTARY TABLE 4| CROSS REGIONAL HUMAN AGING ALTERED GENES, RELATED TO FIGURE 3 15

SUPPLEMENTARY TABLE 5| AGE ASSOCIATED CELL SPECIFIC GENES, RELATED TO FIGURES 4 AND 7 15

SUPPLEMENTARY TABLE 6| AGE ASSOCIATED CELL SPECIFIC GENES, RELATED TO FIGURE 4 16

SUPPLEMENTARY TABLE 7| AGING ALTERED GENES THAT WERE ALSO FOUND AS METHYLATED UPON AGING (BASED ON COMPARISON TO OTHER DATASETS), RELATED TO FIGURES 4 AND S7 16

8. SUPPLEMENTAL REFERENCES 17

1. Supplementary experimental procedures

Study data sets

RNA expression data sets

The UKBEC data set

Brain tissues originated from 134 Caucasian European individuals (with two exceptions, 1 of Mexican ancestry and 1 of Chinese ancestry, see further details under Table S1). Of these, 101 brains were obtained from the MRC sudden Death Brain and Tissue Bank, Edinburgh, UK (Millar et al., 2007) 33 of them originating from the Sun Health Research Institute (SHRI, USA), an affiliate of Sun Health Corporation, USA (Beach et al., 2008). None of the individuals had neuropathologically diagnosable conditions, or presented with neurological or neuropsychiatric conditions. A detailed description of the samples used in the study, tissue processing and dissection is provided in the in Trabzuni et al. (Beach, 2008; Trabzuni et al., 2013). All the samples had fully informed consent for retrieval and were authorized for ethically approved scientific investigation (National Hospital for Neurology and Neurosurgery and Institute of Neurology Research Ethics Committee, 10/H0716/3).

The brain samples were extracted post mortem from up to 10 brain regions per individual. The individuals aged from 16 (the youngest) to 102 years. For each individual, the samples were produced from up to 10 different anatomical brain regions: Frontal cortex (FCTX) from Brodmann areas 8 and 9, Cerebellar cortex (CRBL), Hippocampus (HIPP), Substantia Nigra (SNIG), Putamen (PUTM), Thalamus (THAL), Medulla (specifically, the inferior olivary nucleus) (MEDU), Temporal Cortex (TCTX) and the Occipital cortex (OCTX) from Brodmann area 17 and from intralobular white matter (WHMT, which was separated from the grey matter). In terms of aging altered genes, we detected genes that were detected as

differentially expressed in different brain regions, subsets of brain regions or in a brain-wide fashion and aging altered cell specific genes.

The NABEC data set

To further increase the number of samples and add an external, independent dataset for computational validation purposes, we examined a large additional independent dataset of 607 post mortem brain samples originating from 305 control individuals (N = 101 females and 204 males) between the ages 16 to 101 that had no neuropathological diagnosis nor neurological or neuropsychiatric conditions. Sub-dissected samples from cerebellar and frontal cortex brain regions were frozen before processing (Ramasamy et al., 2013a). The dataset included an overall of 607 Illumina HT12 v3 BeadChip microarray raw CEL files, which interrogated RNA from 305 post mortem FCTX brain samples, and 302 CRBL samples from the same individuals.

REST FCTX data set

RNA extraction from 39 human cortical samples, as well as microarray hybridization protocols were previously described under (Loerch et al., 2008), the minimal age was 24 and the maximal - 106. The microarray platform used is Affymetrix Human Genome U133plus 2.0 Arrays. Postmortem human cortical samples were derived from subjects that did not carry a diagnosis of Alzheimer’s disease or another neurodegenerative disease, and showed neuropathological findings within the normal range for age (Loerch et al., 2008). Overall, we analyzed up to 10 brain regions from 480 individuals aged from 16 to 106 and over 1800 microarray samples.

RNA Isolation

UK-BEC exon arrays: RNA Extraction is described under Trabzuni et al. (Trabzuni et al., 2011).

NABEC 3' arrays: the RNA isolation and array processing as well as quality measurements are reported in Ramasamy et al. (e.g (Ramasamy et al., 2013a), (Hernandez et al., 2012)).

High-resolution immunohistochemical brain imaging dataset production

To complement out RNA expression findings, we have generated high resolution imaging data for 6 selected BA9 (FCTX) samples using OLIG2 antibody (to stain oligodendrocytes (OLGs), where the DAB+ nuclear product is seen brown) and with Heamotoxylin as a counterstain (the nuclei of other cell types are stained in blue). Briefly, the brain sections were placed into 2 changes of Xylene to remove wax – 3 mins each, with varying degrees of alcohol (70%, 99% and absolute alcohol) to rehydrate – 3 mins each. The samples were then placed in picric acid to remove any artifacts – ca15 minutes. Subsequently, the samples were placed under running water to remove all picric residues (ca15 mins). Finally, antigen retrieval was performed, 1:200 with Citric Acid retrieval (5% Citric Buffer pH 6.0 heated to 125°C for 30 seconds then allowed to cool).

The remaining process was done via the Leica Novolink Polymer Detection Kit:

1. Peroxidase Block – 30 mins
2. Tris Buffered Saline – 5 mins
3. Protein Block – 15 mins
4. Tris Buffered Saline – 5 mins
5. Primary Antibody – 30 mins
6. Tris Buffered Saline – 5 mins
7. Post Primary Block – 30 mins
8. Tris Buffered Saline – 5 mins

9. Novolink Polymer – 30 mins
10. Tris Buffered Saline – 5 mins
11. DAB secondary (50ul chromogen/1ml substrate buffer)
12. Counterstained with Heamotoxylin – 30 seconds

Slides then dehydrated, through to Xylene and mounted.

The images were acquired on the 'Zeiss AxioScan Slide Scanner.

Details and specs of the system can be found here:

www.zeiss.co.uk/microscopy/en_gb/products/imaging-systems/axio-scan-z1.html

Magnification of the scans done set to x20 and in the Brightfield setting.

NeuN slides staining

In the NeuN staining protocol, antibody used was from Acris (code AM10122SU-N) in dilution of 1:200. NeuN antibody was Acris AM10122SU-N was used at 1:200 dilution with citrate buffer pre-treatment for antigen retrieval. The retrieval method was Leica Bond ERI solution for 20 minutes (citric acid @6pH). All pre-treatments including dewaxing and counterstains (haematoxylin) were carried out on the Leica Bond III IHC staining machine. The staining protocol used is Protocol F (factory set one).

Image acquisition of the high-resolution FCTX OLIG2 stained images

Post-mortem human brain sections were placed into 2 changes of Xylene to remove wax (3 minutes each). Varying degrees of alcohol (70%,99% and absolute) used to rehydrate (3 minutes each), placed in Picric Acid to remove any artifacts and were put under running water to remove all picric residue (ca 15 minutes). Antigen retrieval was performed subsequently, 1:200 with Citric acid for the OLIG2 staining (citric acid: 5% Citric Buffer pH 6.0 heated to 125oC for 30 seconds then allowed to cool). The Leica Novolink Polymer detection kit was then applied as follows: Peroxidase Block - 30 minutes, Tris buffered Saline – 5 minutes, Protein block – 15 minutes, Tris buffered Saline – 5 minutes, Primary

antibody – 30 minutes, Tris Buffered Saline – 5 minutes, Post primary block – 30 minutes, Tris buffered Saline – 5 minutes, Novolimk Polymer – 40 minutes, Tris buffered Saline – 5 minutes and DAB secondary (50ul chromogen/1ml substrate buffer), Counterstained with Heamotoxylin – 30 seconds and slides were then dehydrated, through to Xylene and mounted. The images were acquired on the 'Zeiss AxioScan Slide Scanner. Details and specs of the system can be found here: www.zeiss.co.uk/microscopy/en_gb/products/imaging-systems/axio-scan-z1.html. Magnification of the scans done set to x20 and in the Brightfield setting.

NeuN stained sections image acquisition

Image acquisition from the FCTX NeuN stained sections was done similarly to the OLIG2 stained samples, the images were acquired on the 'Zeiss AxioScan Slide Scanner. In order to account for the large diversity of cell size and shape, in the quantification of neuronal cells based on the NeuN stained slides, we increased tile size from the native device tile 1600 x 1200 to a larger tile of 10k x10k pixels. This was followed assessing the entropy of each slide, a statistical measurement that captures the randomness across grayscale image using the following formula $E = -\sum p_i log(p_i)$ and excluding any image below entropy of 5. Then we preform element wise multiplication across the inverse red and blue image channel to create the 16bit interaction image that contains both NeuN staining as well as the supporting cells all transformed from local minima to local maxima – converting "valleys to hills". This is followed by estimating five multilevel thresholds using Otsu method (Otsu, 1975). And creating a segmented binary image using the maximum threshold. Morphological operations are followed to ensure segmentation integrity and areas smaller then a lower bound of 500 pixels is enforced.

This is followed by applying the watershed transform (Meyer, 1994) to separate joined segments. And finished by harvesting additional per cell statistics. Visual plots are produced per tile to enable

visual inspection of the quality of cell detection. The raw .jpeg images of the NeuN stained slides can be seen under FigShare portal (link: https://figshare.com/s/f2675361af1242f3565f).

ANALYSIS METHODS

Microarrays

To analyze these extensive datasets, we developed dedicated programmed computer programs. Specifically, we have implemented a number of functions and scripts for mathematical, computational and statistical analyses of the microarrays and RNASeq data, including (1) definition of age groups based on histogram bins (16-44 years old, 45-79 years old and 80 – 106 years old), (2) microarray processing including identification of gene differentially expressed upon aging in each brain region, (3) comparisons between different brain regions, (4) expression correlations, (5) classification (6) enrichment analysis of the RNASeq data from specific cell types, (7) cellular specificity of the aging altered genes and (8) data visualization. These tailored mathematical and statistical analyses were conducted through tailored Matlab (version R2013B) programs that also called functions from Matlab Statistics toolbox. First, we divided the UKBEC microarray samples to three age groups (young, middle and old) by a mathematical calculation, using histogram bins of all the ages that were included in the dataset. Subsequently, significance p-values were computed using ANOVA (and FDR correction, $q < 1e-3$) based on the gene level expression signals of the interrogated genes. Subsequently, partial least squares regression (PLSR) linear regression model was further used to rank the aging altered genes based on the age prediction accuracy of these genes. We then applied unsupervised clustering and classification methods on the expression data, to investigate grouping of the samples of different age groups and brain regions based on expression signals of different groups of genes. Those methods included hierarchical classification (HCL), which was applied using the Euclidean distance and average linkage method. We also applied a powerful non-linear parametric dimensionality reduction technique called t-Distributed Stochastic Neighbor Embedding (t-SNE) (Laurens van der Maaten, 2008) to create a 2D visualization of local and global data structures based on different groups of genes identified as altered in the aging brains including

cell type top markers. The technique is a variation of the Stochastic Neighbor Embedding method (termed SNE, Hinton and Roweis, 2002), with the aim to preserve as much of the significant structure of the high dimensional data as possible in a low dimensional map. SNE minimizes the sum of Kullback-Leiber divergences over all data points using a gradient descent method. The variance of the Gaussian noise is reduced through iterative process. We applied support vector machine (SVM) to classify the samples to age groups based on the expression patterns of the genes commonly altered in all the brain regions upon aging. Functional enrichment analysis to detect enriched functional groups of genes was conducted through the DAVID (Dennis et al., 2003) resource EASE (Hosack et al., 2003) program on the lists of genes found as altered in different brain regions upon aging, as well as to functionally analyze cell specific aging genes. To measure relationships among the annotation terms based on the degrees of their co-association genes the similar, redundant, and heterogeneous annotation contents from the same or different resources were grouped into annotation groups. A modified Fisher Exact P-value calculated for gene-enrichment analysis. $P < 0.05$ considered as significant. The number of differentially expressed genes per term was compared against the human genome background.

Cell type specific genes: definition and classification analyses

Cell-type specific genes were defined by analysis of RNASeq data from mouse brain (http://web.stanford.edu/group/barres_lab/brain_rnaseq.html) through calculation of enrichment score based on the normalized RNASeq read counts from the mice cortex RNA libraries. We calculated the enrichment p-value for the 7 cell types (which included neurons and glial types), per each separately. The full list of markers is given under Table S5. We further used the defined lists of genes expression profiles to find age predictive genes (Table S6). Additional cell specific lists were based on a previous microarray data on four cell types (Cahoy et al., 2008). Additionally, we used expression data from 24 central nervous system cell types interrogated by microarrays was to classify the brain samples

(http://web.stanford.edu/group/barres_lab/brainseqMariko/brainseq2.html), Table S7. Further details are under supporting material.

3. High resolution scanned immunohistochemical stained images computational quantification

OLIG2 stained brain sections image analysis

To analyze slides produced by high throughput imaging on brain sections from selected old and young individuals (N=3 of each age group), a tailored systematic computational pipeline was developed using Matlab (R2015a). The pipeline preforms a cell counting procedure for each independent tile, followed by statistical permutation assessment. The quantification module includes the following steps: 1. For slide in each sample (typically composed of several thousands of individual multi-channel images here called slides): For each slide, entropy (i.e. $H = -\sum_{i=1}^{M} p_k \log_2(p_k)$) was calculated and the exclusion criteria of H=5 was used to discard slides without useful information. 2) Morphological top-hat and bottom-hat filtering using a disk kernel was performed to account to local noise. 3) A pyramidal Gaussian mixture model (k=4) was performed on the slide to label each pixel and using a majority vote across pyramidal levels a binary image was produced. 4) Morphological procedures were used to discard border elements as well as hole filling, and lower and upper area thresholds. 5) Watershed algorithm was performed to separate overlapping cells. 6) Each estimated cell was classified using an expertly trained classification model (see below) to further eliminate non-cell objects.

Cell quantification and classification model

For analysis of OLG, the tiles of each OLIG2 stained image were extracted. Images with low entropy were discarded (<5) and the information based filtering was followed by background and noise filtering.

Then the lower threshold was uncovered using empirical Pareto distribution modelling, and thresholding of the three color channels using this measurement to create a binarized segmentation of the image. This was followed by circle detection using circular Hough transform. Lastly, each circle was assigned a class using a previously trained random forest model based on manual expert classification of 2000 events (i.e stained cells) containing both brown- and blue- stained noise ones. For each slide, the output included a summary of the number of cells counted as true in it, per each of four density groups representing four different cell densities. The difference between age groups was calculated using permutation tests on the average number of cells per each density class. For training of the machine-learning algorithm, about 2800 individual cells were manually labeled as 'blue', 'brown' (the OLG stained cells through the DAB+ nuclear product), or 'other' (which represents technical artifacts/noise). This data was used to train a Random Forest learning algorithm and to generate a classification model.

For analysis of neurons, we accounted for the large diversity of cell size and shapes by increasing the tile size from the native device tile. We assessed the entropy of each slide, and assessed statistically the entropy of each slide for filtering purposes, preformed element wise multiplication across the inverse red and blue image channel, estimated five multilevel thresholds and application of watershed transform. Area statistics per slide is aggregated into one large database, and data is binned into four size groups. For each bin, 100 random iterations over 1000 permutation analysis are performed to assess the empirical p-value over the null T-distribution. In order to account for the large diversity of cell size and shape, in the quantification of neuronal cells based on the NeuN stained slides, we increased tile size from the native device tile. Briefly, assessed the entropy of each slide, and assessed statistically the entropy of each slide for filtering purposes, preformed element wise multiplication across the inverse red and blue image channel, estimated five multilevel thresholds and application of watershed transform. Further details about the computational methods for quantification of neurons are given under the supporting methods.

Permutation test on the OLIG2 stained slides

SUPPLEMENTAL INFORMATION

Each image is constructed from thousands of tiled images each approximately 0.6mm wide and 0.46mm tall. For each tile, the number of cells was extracted. Statistical significance was calculated on groups for both types of stained cells on overall 10,922 aging and 8,766 young stained tiles. To account for the large imbalance in the total number of slides we randomly selected an equal number of 100 tiles per sample, and repeated this process across 100 iterations and 1000 random shuffling permutations. To further examine the effects of the two different cellular populations in BA9 (likely corresponding to gray matter cortical areas vs. the least dense, WHMT) we classified the different tiles based on the total cell density (regardless of sample) to four different groups based on density (low, low-med, med-high and high which generally may be attributed to white matter vs. grey matter areas). The significance p-value was calculated using normal distribution cumulative distribution function (CDF) per permutation using the following formula $T_n = \frac{T_0 - \bar{T}}{std(T)}$ where T_0 corresponds to the true labels.

Overall 6 BA9 (FCTX) samples sections stained with OLIG2 were analyzed (3 young and 3 old samples). In total, 49,822 stained FCTX (BA9) image tiles were captured for all samples (number of image tiles in the old brains = 24,061, and in young ones = 25,761). Overall 20,190 tiles survived signal to noise ratio (SNR) exclusion criteria (old = 8,992, young = 11,198). Generally, the cell detection and quantification pipeline included the following steps: SNR exclusion criteria, image enhancement, followed by object detection of all the cells in each slide This process was performed using a non-linear machine-learning model that was created using a large dictionary of positive and negative examples annotated manually by experts (general computational flow under Figure 5B). For each slide, the number of cells as well as morphological features per cell (including area, perimeter and roundness) was extracted. Overall, 393,625 cells (old = 172,868, young = 220,757) were quantified. Of these, 183,898 were brown (OLG precursor) cells (old = 76,926, young =106,972) and 209,727 were blue (old = 95,942, young =113,785). In the high-density slides we randomly selected 50 slides from each sample and run a

similar permutation test to the one above (old = 1828, young = 2612). FDR correction over the p-values was performed to account to the multiple iterations. The tiles were classified to 4 different density groups (low, low-med, low-high and high) using k-means. Further details are under supporting methods.

Permutation tests on the NeuN stained slides

Per-slide Area statistics are aggregated into one large database, and data is binned into four size groups. For each bin 100 random iterations over 1000 permutation analysis are performed to assess the empirical p over the null t distribution.

4. Cell type marker genes and age-association statistical analysis

The cell type gene markers for seven cell types were defined based on statistical analysis of available RNA-Sequencing data produced by the Barres lab (http://web.stanford.edu/group/barres_lab/brain_rnaseq.html), Table S3. For each group of cell type markers, an age predictive model was generated. Briefly, the UKBEC cohort samples were partitioned into training (60%), testing (20%) and validation (20%) sets, and gender, brain bank source and cause of death were incorporated as covariates. Following variable elimination using Competitive adaptive reweighted sampling (CARS), the model was assessed and stepwise fit was calculated.

5. Supplementary Tables list and legends

Supplementary Table 1| UKBEC cohort samples details, related to Figure 1

The details for the UKBEC samples and microarray sample numbers are given for each of the 1,231 analyzed brain samples. The details include: brain bank, individual ID, central nervous system (CNS) region, RNA Integrity number (RIN), chronological age, Post Mortem Interval (PMI) - in hours, brain tissue pH, cause of death code, gender and ethnicity. Reference in the main paper text: page 5, line 8.

Supplementary Table 2| Functional enrichment of brain aging altered genes, related to Figure 2

Results of functional enrichment analysis based on the genes that were detected as altered in each of the analyzed 10 brain regions upon aging, and of the genes commonly altered in 8-10 brain regions upon aging. For each brain region, the top 200 genes were functionally analyzed. The functional analysis was conducted using the DAVID resource EASE tool (Hosack et al., 2003), and with Gene Analytics LifeMap tool (Edgar et al., 2013). The functional analysis was conducted for each brain region, on the top 200 aging altered genes (scores were calculated using a PLSR model) for each of the 10 brain regions. Reference in the main paper text: page 14, line 22.

Supplementary Table 3| Cell type marker genes altered in the aging brain, related to Figure 4

Genes found as top enriched brain cell markers based on t-test of average RPKM values of RNASeq mouse transcriptome data of glia, neurons and vascular cells of rats cerebral cortex for 7 cell types: endothelial cells, neurons, oligodendrocyte (OLG) precursors, newly formed OLGs, myelinating OLGs, microglia and astrocytes (Zhang et al., 2014a), that were also found as altered in the human brain upon aging. Specific cell type marker genes that were also altered in the brain upon aging, including cell type

specific enrichment score and p value. The specific cell type marker genes were identified by statistical analysis of the RNASeq data produced by the Barres laboratory from seven rat brain cell types. The Barres lab RNA brain RNASeq data resource can be found under: http://web.stanford.edu/group/barres_lab/brain_rnaseq.html). Normalized RNASeq counts were analysed (Zhang et al., 2014b)).

To complement our cell specific findings, we also analyzed the recent Barres brain RNASeq transcriptome dataset (published in Neuron, 2016, This data was produced from human fetal brains, and includes neurons, microglia, astrocytes and endothelial cells, and from several brain regions (temporal cortex, white matter, parietal cortex, and hippocampus). We used the normalized FPKM data for our analyses. This recent cell specific data is available under: web.stanford.edu/group/barres_lab/brainseqMariko/brainseq2.html.

Additionally, we used the microarray data of 24 Central Nervous System (CNS) populations, of the resource that was published by Doyle et al. (Doyle et al., 2008)to define additional lists of cell type specific genes and investigate their expression patterns upon aging using our dataset. Reference in the main paper text: page 8, line 15.

Supplementary Table 4| Cross regional human aging altered genes, related to Figure 3

This table legend is given directly following the table, which is in this file. Reference to the table in the main paper text: page 13, line 21.

Supplementary Table 5| Age associated cell specific genes, related to Figures 4 and 7

Cell type specific markers that were found by statistical enrichment analysis of RNASeq data from rat brains (reference: Zhang, Y., et al., An RNA-sequencing transcriptome and splicing database of glia, neurons, and vascular cells of the cerebral cortex. J Neurosci, 2014. 34(36): p. 11929-47) (Zhang et al., 2014b), for each of the 7 analyzed cell type populations. For each gene, given are the gene symbol, intercept estimate, standard error, T statistic and p-value. Reference in the main paper text: page 20, line 17.

Supplementary Table 6| Age associated cell specific genes, Related to Figure 4

Cell type specific markers that were found as age predictive by a linear model, for each of the 7 analyzed cell type populations. For each gene, given are the gene symbol, intercept estimate, standard error, T statistic and p value. Reference in the main paper text: page 20, line 17.

Supplementary Table 7| Aging altered genes that were also found as methylated upon aging (based on comparison to other datasets), Related to Figures 4 and S7

Upon comparison to the aging methylation/expression study data (reference: (Horvath, 2013)), a large number of the reported aging methylated brain genes were also found in our study as differentially expressed upon aging. Additionally, genes that we detected as altered upon aging that were also found as top neuronal cell type markers (based on Doyle et al 24 cell type microarray study (reference: Application of a translational profiling approach for the comparative analysis of CNS cell types, Doyle J. P. et al., Cell 2008) are given as well. Reference in the main paper text: page 23, line 16.

6. EXPRESSION DATASETS ACCESSION CODES

UKBEC Gene Expression Omnibus (GEO): GSE36192

NABEC - see under (Ramasamy et al., 2013b) and GEO accession number: GSE36192.

8. SUPPLEMENTAL REFERENCES

Beach, T.G., Sue, L.I., Walker, D.G., Roher, A.E., Lue, L., Vedders, L., Connor, D.J., Sabbagh, M.N., and Rogers, J. (2008). The Sun Health Research Institute Brain Donation Program: description and experience, 1987-2007. Cell and tissue banking *9*, 229-245.

Beach, T.G.e.a. (2008). The Sun Health Research Institute Brain Donation Program: description and experience, 1987-2007. Cell Tissue Bank *9*, 229-245.

Cahoy, J.D., Emery, B., Kaushal, A., Foo, L.C., Zamanian, J.L., Christopherson, K.S., Xing, Y., Lubischer, J.L., Krieg, P.A., Krupenko, S.A., *et al.* (2008). A transcriptome database for astrocytes, neurons, and oligodendrocytes: a new resource for understanding brain development and function. J Neurosci *28*, 264-278.

Dennis, G., Jr., Sherman, B.T., Hosack, D.A., Yang, J., Gao, W., Lane, H.C., and Lempicki, R.A. (2003). DAVID: Database for Annotation, Visualization, and Integrated Discovery. Genome biology *4*, P3.

Doyle, J.P., Dougherty, J.D., Heiman, M., Schmidt, E.F., Stevens, T.R., Ma, G., Bupp, S., Shrestha, P., Shah, R.D., Doughty, M.L., *et al.* (2008). Application of a translational profiling approach for the comparative analysis of CNS cell types. Cell *135*, 749-762.

Edgar, R., Mazor, Y., Rinon, A., Blumenthal, J., Golan, Y., Buzhor, E., Livnat, I., Ben-Ari, S., Lieder, I., Shitrit, A., *et al.* (2013). LifeMap Discovery: the embryonic development, stem cells, and regenerative medicine research portal. PLoS One *8*, e66629.

Hernandez, D.G., Nalls, M.A., Moore, M., Chong, S., Dillman, A., Trabzuni, D., Gibbs, J.R., Ryten, M., Arepalli, S., Weale, M.E., *et al.* (2012). Integration of GWAS SNPs and tissue specific expression profiling reveal discrete eQTLs for human traits in blood and brain. Neurobiology of disease *47*, 20-28.

Horvath, S. (2013). DNA methylation age of human tissues and cell types. Genome Biol *14*, R115.

Hosack, D.A., Dennis, G., Jr., Sherman, B.T., Lane, H.C., and Lempicki, R.A. (2003). Identifying biological themes within lists of genes with EASE. Genome Biol *4*, R70.

Laurens van der Maaten, G.H. (2008). Visualizing Data using t-SNE Journal of Machine Learning Research *9*, 2579-2605

Loerch, P.M., Lu, T., Dakin, K.A., Vann, J.M., Isaacs, A., Geula, C., Wang, J., Pan, Y., Gabuzda, D.H., Li, C., *et al.* (2008). Evolution of the aging brain transcriptome and synaptic regulation. PloS one *3*, e3329.

Meyer, F. (1994). Topographic distance and watershed lines. Signal processing *38*, 113--125.

Millar, T., Walker, R., Arango, J.C., Ironside, J.W., Harrison, D.J., MacIntyre, D.J., Blackwood, D., Smith, C., and Bell, J.E. (2007). Tissue and organ donation for research in forensic pathology: the MRC Sudden Death Brain and Tissue Bank. The Journal of pathology *213*, 369-375.

Otsu, N. (1975). A threshold selection method from gray-level histograms. Automatica *11*, 23-27.

Ramasamy, A., Trabzuni, D., Gibbs, J.R., Dillman, A., Hernandez, D.G., Arepalli, S., Walker, R., Smith, C., Ilori, G.P., Shabalin, A.A., *et al.* (2013a). Resolving the polymorphism-in-probe problem is critical for correct interpretation of expression QTL studies. Nucleic Acids Res *41*, e88.

Ramasamy, A., Trabzuni, D., Gibbs, J.R., Dillman, A., Hernandez, D.G., Arepalli, S., Walker, R., Smith, C., Ilori, G.P., Shabalin, A.A., *et al.* (2013b). Resolving the polymorphism-in-probe problem is critical for correct interpretation of expression QTL studies. Nucleic acids research *41*, e88.

Trabzuni, D., Ramasamy, A., Imran, S., Walker, R., Smith, C., Weale, M.E., Hardy, J., Ryten, M., and North American Brain Expression, C. (2013). Widespread sex differences in gene expression and splicing in the adult human brain. Nature communications *4*, 2771.

Trabzuni, D., Ryten, M., Walker, R., Smith, C., Imran, S., Ramasamy, A., Weale, M.E., and Hardy, J. (2011). Quality control parameters on a large dataset of regionally dissected human control brains for whole genome expression studies. J Neurochem *119*, 275-282.

Zhang, Y., Chen, K., Sloan, S.A., Bennett, M.L., Scholze, A.R., O'Keeffe, S., Phatnani, H.P., Guarnieri, P., Caneda, C., Ruderisch, N., *et al.* (2014a). An RNA-sequencing transcriptome and splicing database of glia, neurons, and vascular cells of the cerebral cortex. J Neurosci *34*, 11929-11947.

Zhang, Y., Chen, K., Sloan, S.A., Bennett, M.L., Scholze, A.R., O'Keeffe, S., Phatnani, H.P., Guarnieri, P., Caneda, C., Ruderisch, N., *et al.* (2014b). An RNA-sequencing transcriptome and splicing database of glia, neurons, and vascular cells of the cerebral cortex. The Journal of neuroscience : the official journal of the Society for Neuroscience *34*, 11929-11947.

Figure S1. Related to Figures 2 and 3.

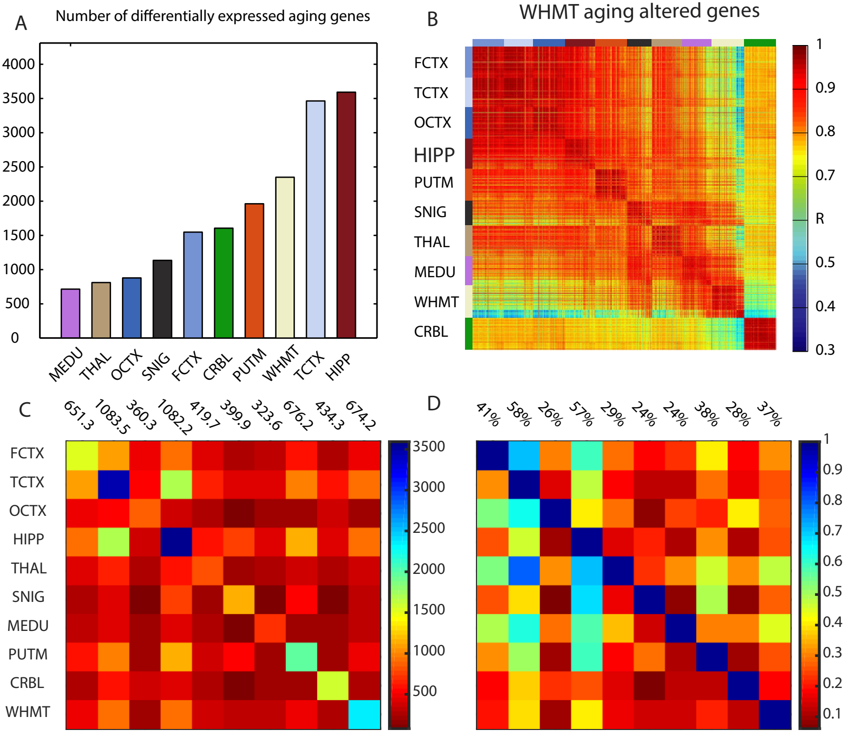

Figure S1

Related to Figures 2 and 3.

A) The number of altered genes per brain region in the main dataset of UKBEC (FDR<1e-3) B) A correlation between all the analyzed 1,231 UKBEC samples based on gene expression signals of genes that were altered upon aging in the white matter (WHMT). C) A heatmap showing the number (left) and percent (right) of brain aging altered genes that were commonly altered in two brain regions of the 10 studied concurrently.

Figure S2. Related to Figure 3.

A Region-specific

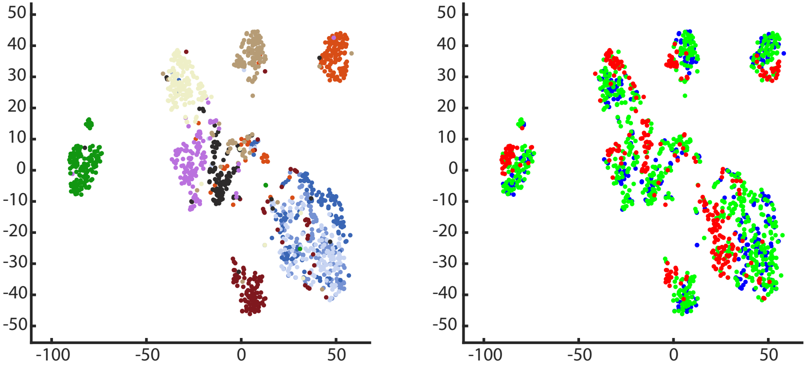

B Altered in 2-7 regions

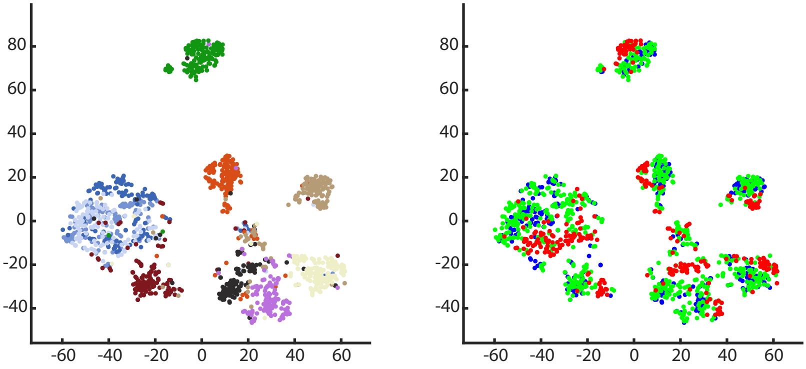

C Multi-Regional

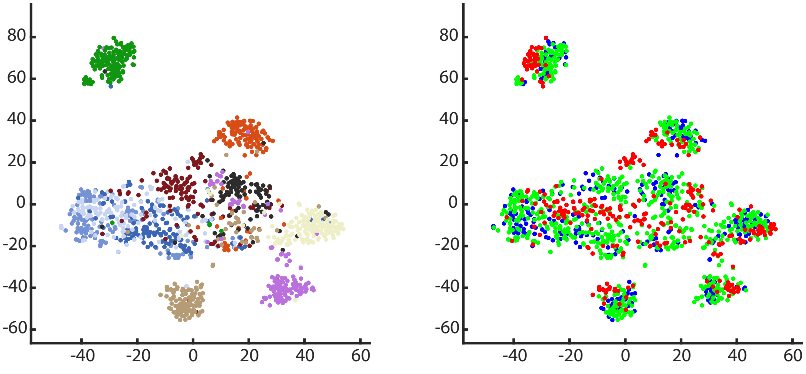

Figure S2

Related to Figure 3.

Classification by a multi-dimensional reduction classification algorithm of the UKBEC samples based on the expression patterns of genes altered in a single brain region, commonly altered in 2-7 brain regions and in 8-10 regions upon aging (B). The samples are much less separated by region based on expression of the multi-regional altered genes (commonly altered in 2-8 regions). In each sub section, the right panel presents the samples colored by age group (young, middle, old).

Figure S3. Related to Figure 3.

A SVM on NABEC based on cross-regional genes

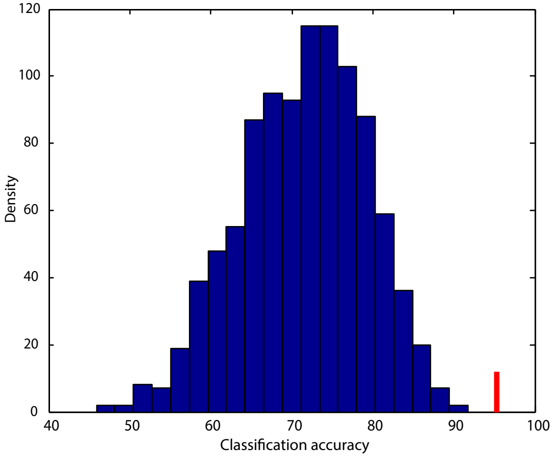

B Age-group classification colored by gender based on cross-regional genes

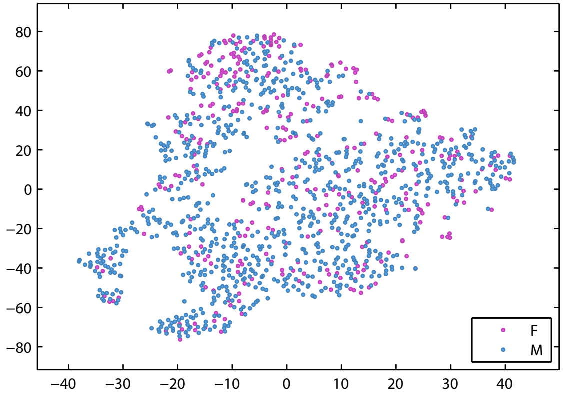

C REST dataset age-group classification by cross-regional genes

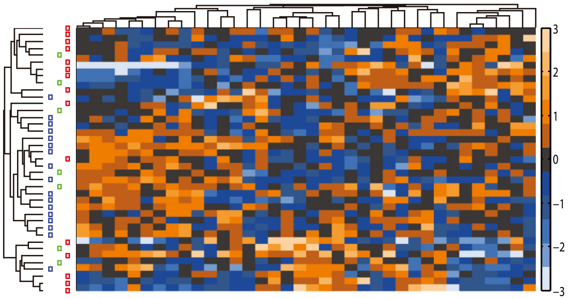

Figure S3

Related to Figure 2.

Support vector classification (SVM) of the independent NABEC dataset based on the expression patterns of a small subset of genes commonly altered in all the analyzed UKBEC brain regions (A). (B) The UKBEC samples are not classified by gender based on the RNA expression profiles of the small subset of aging altered common genes. (C) The second independent dataset (REST dataset) samples are largely classified by age group based on the expression profiles of the small subset of shared genes.

Figure S4. Related to Figures 3 and 7.

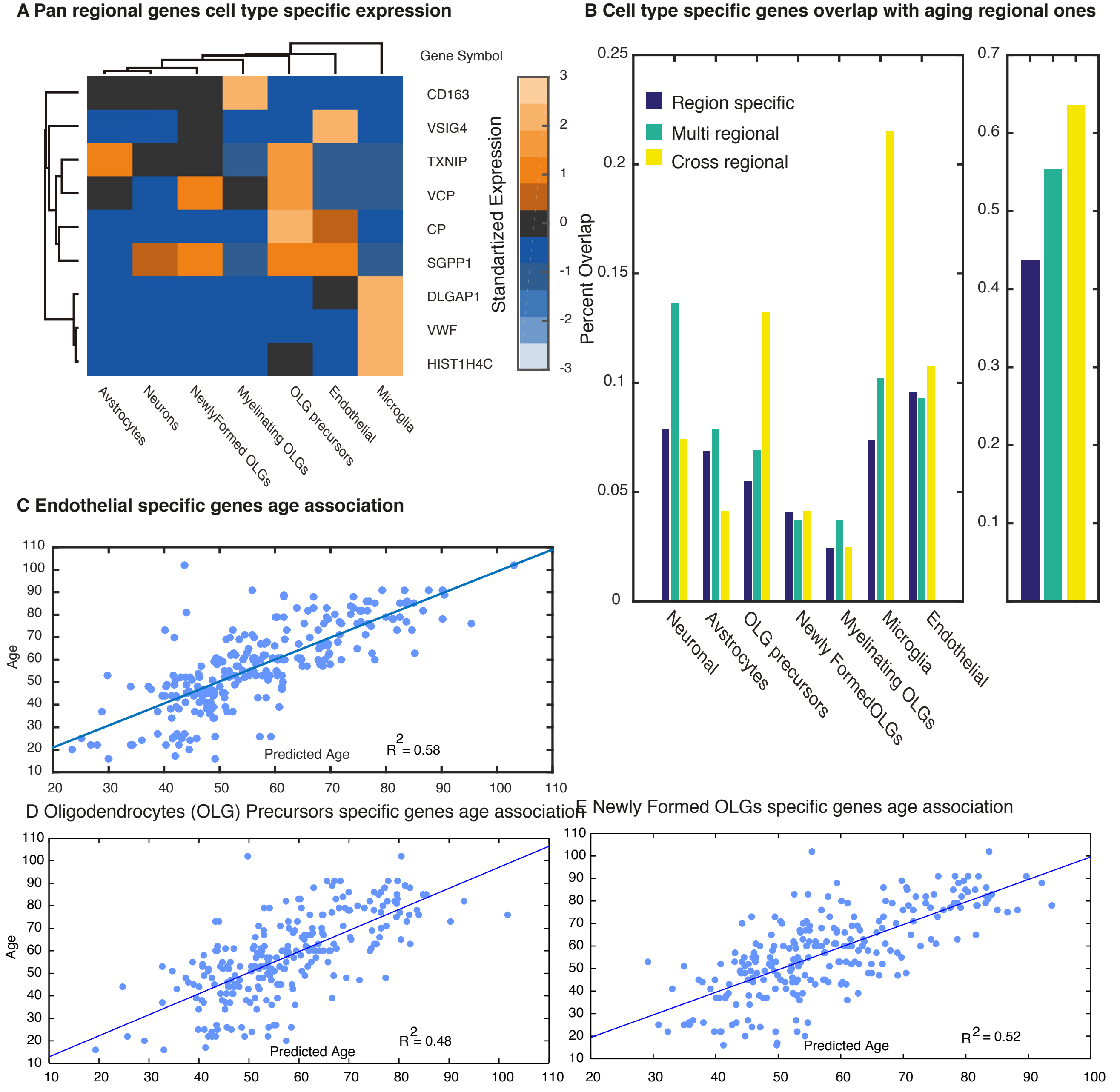

Figure S4

Related to Figures 3 and 7.

The pan-regional aging altered genes were enriched in 7 cell type (RNASeq data (from http://web.stanford.edu/group/barres_lab/brain_rnaseq.html). The hierarchical classification Euclidian distance dendograms are shown as well. Color scale: z-score of count data. B) The single-regional aging genes were more enriched in neuronal specific genes, while multi regional aging genes were more enriched in OLG precursor and microglia specific genes (left). Overall, multi-regional genes were more enriched in cell specific markers as compared with both multi- and single- regional genes (right). C) Endothelial (D) OLG precursor and E) Newly formed OLGs specific genes are associated with age. OLG: oligodendrocytes.

Figure S5. Related to Figure 4.

A WHMT aging altered gened enriched GO terms

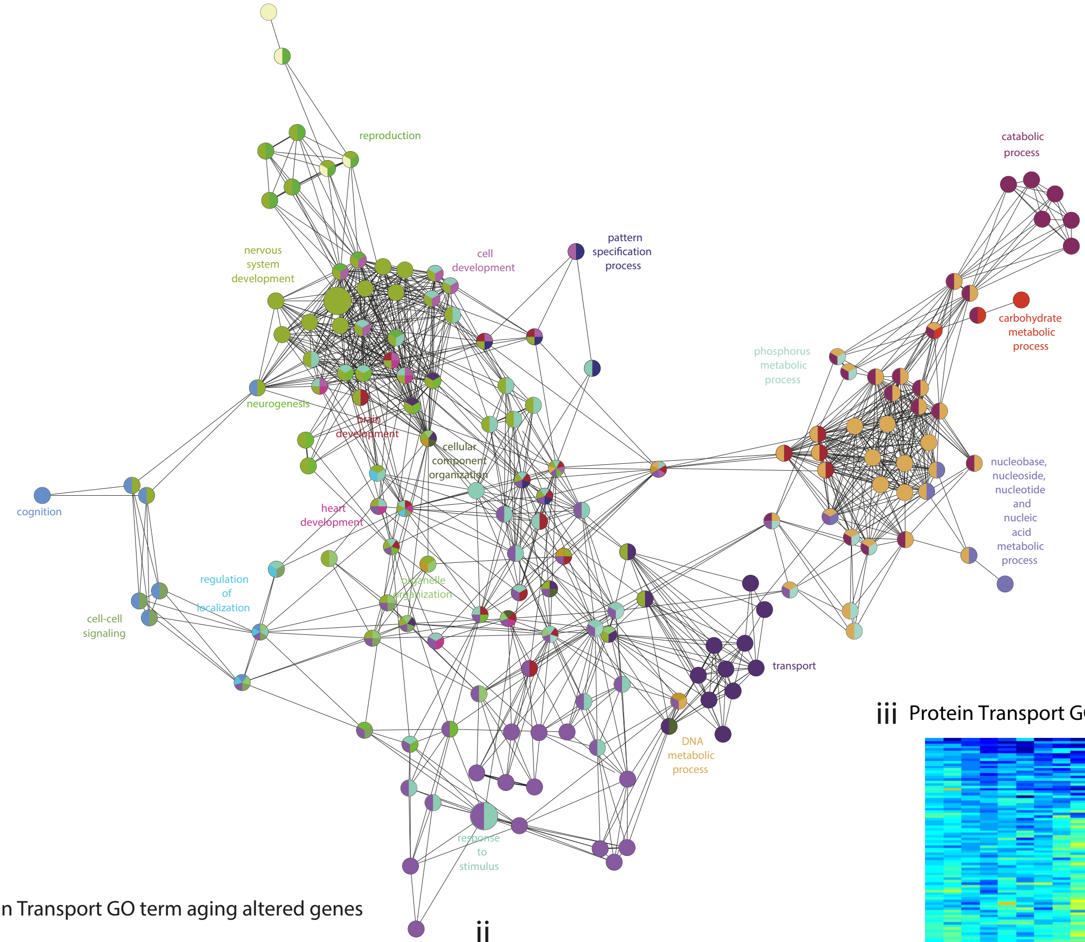

B Protein Transport GO term aging altered genes

i

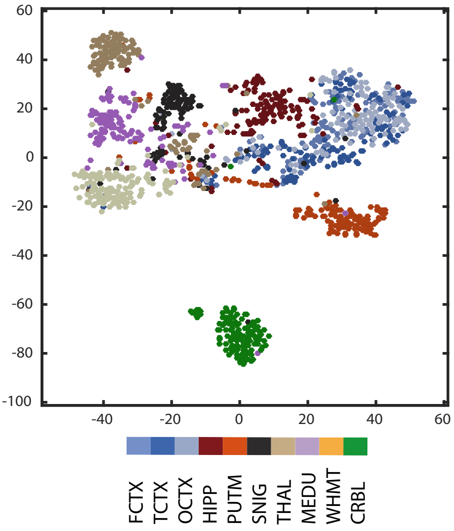

ii

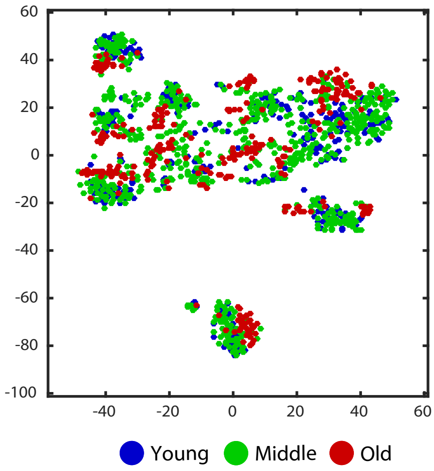

iii Protein Transport GO term genes

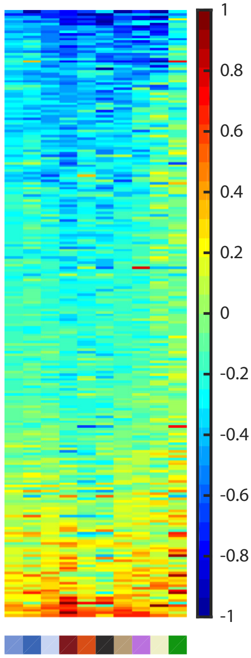

Figure S5

Related to Figure 4.

A) (i) A heatmap of expression of the top 50 MG specific aging altered genes show mainly up regulation ii) The top 50 neuronal specific markers showed a global down regulation, both similarly to the top 100 specific markers (iii) AC specific aging altered genes showed specific down regulation in the SNIG and PUTM iv) The top 50 OLG specific markers showed a mixed pattern of change in aging. In all the panels of A, the left plots show average expression in samples < 60 years old, and the middle plots of > 80 years old. The right panel plots show the fold change ratio (log2) between them (old vs. young). B) (i) A heatmap based on expression signals of top 100 OLG precursor specific aging altered genes (on the z score of the expression values) ii) Classification of the UKBEC samples based on the expression profiles of the top OLG precursor specific aging altered genes, the samples are colored by either brain region (ii) or age group (iii). C) (i) A heatmap based on expression signals of the top 100 Endothelial specific aging altered genes (ii) A classification of the samples based on the expression profiles of top 10 OLG precursor specific aging altered genes, the samples are colored by either region or age group as in A. D) (i) A heatmap of z-score normalized expression signals of the top 100 newly formed OLG specific aging altered genes. ii) Classification based on the top 100 newly formed OLG specific genes, colored by Region (i) and Age (ii). OLG: as in Figure S3 legend.

Figure S6. Related to Figure 4.

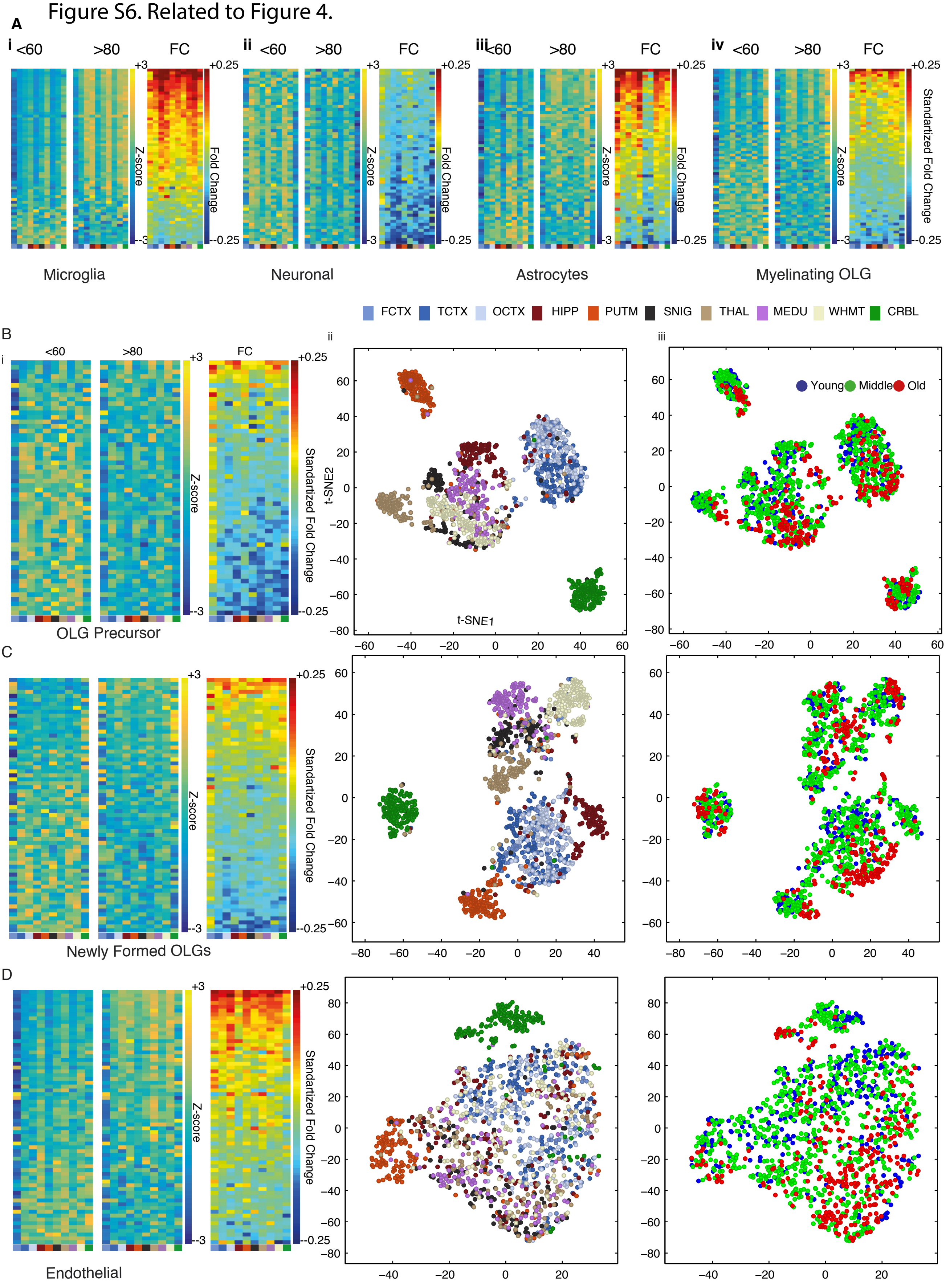

Figure S6

Related to Figure 4.

A) A network based on Gene Ontology (GO) functional enrichment analysis for the WHMT aging altered genes (the analysis was conducted with the Cytoscape plug-in ClueGO (Bindea et al., 2009)). The nodes (functional terms) are colored by functional group. The network is presented in a circular format. B) (i) Classification of all the samples based on the expression of aging altered genes annotated to the GO category protein transport, the samples are painted by regional identity and (ii) by age group. (iii) A heatmap of protein category aging altered genes show mainly down regulation upon aging.

Figure S7. Related to Figure 4 and Figure 7

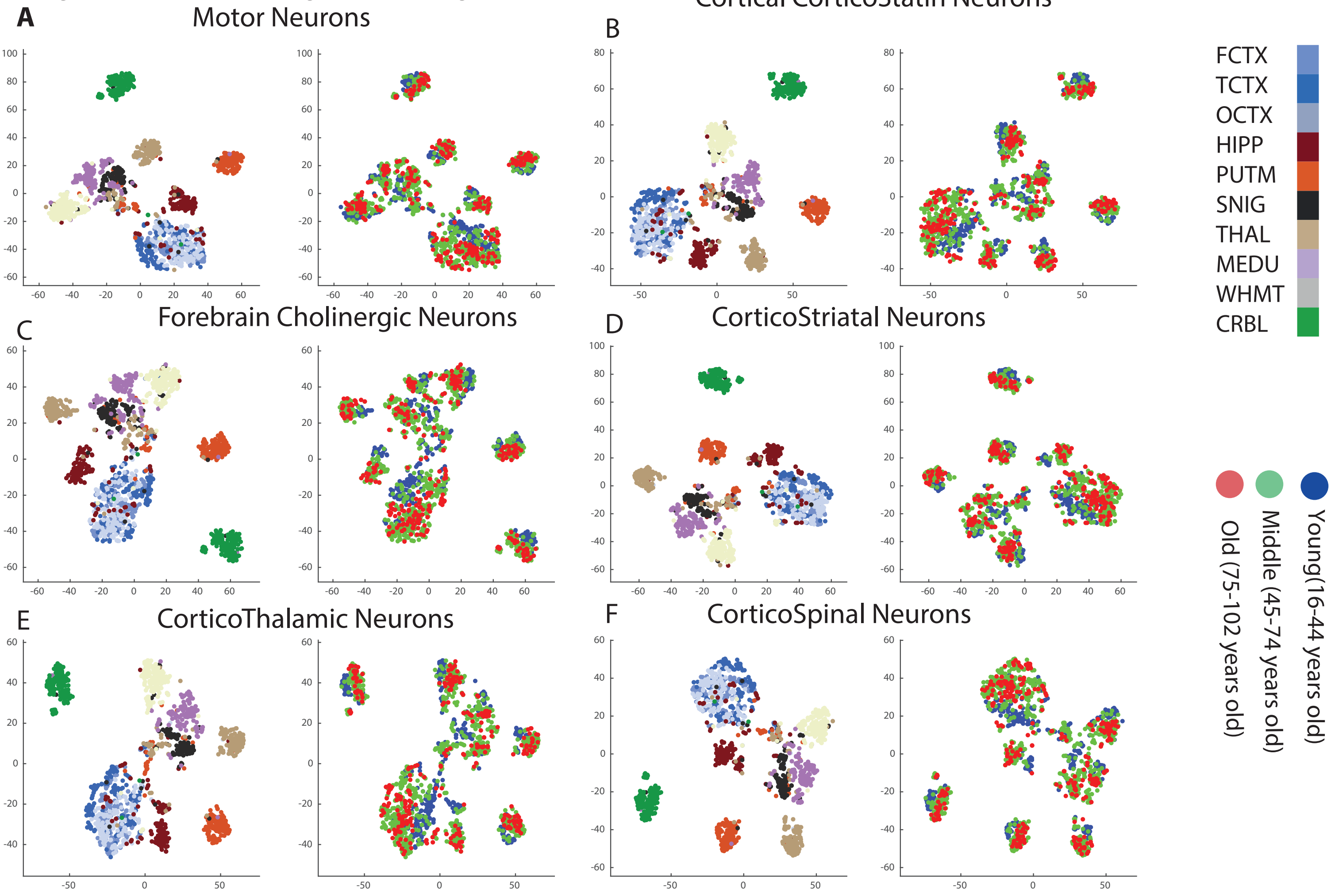

Figure S7

Related to Figure 4 and Figure 7

Classification plots of the UKBEC brain samples based on the expression signals of the neuronal populations specific genes characterized by a previous microarray study on 24 populations (Doyle et al., 2008). Given are examples for 6 different types of neurons. The regional classification showed distinction between the different anatomical regions similarly to one another (and to the plots of neuronal gene markers that were based on our lists that were defined by analysis of publically available rat brain RNASeq data, from http://web.stanford.edu/group/barres_lab/brain_rnaseq.html). Age-group separation was seen in some of the regions (in particular, the PUTM and WHMT as well as CRBL for motor neurons). The classification plots show classification of the samples based on the following aging altered neuronal markers: motor neurons specific genes, cortical CorticoStatin neurons, forebrain cholinergic neurons, corticostriatal neurons, corticoThalamic neurons and corticoSpinal neurons specific genes (A-F). CRBL: cerebellum, PUTM: putamen, WHMT: white matter.

Table S4. Cross-regional aging altered genes (commonly altered in 10 human brain regions)

Soreq L. et al., Major shifts in glial regional identity are a transcriptional hallmark of human brain aging

Related to Figure 3.

#	Gene Symbol	Gene Full Name	Cellular component/ cell specificity	Literature evidence for aging involvement*?	Literature evidence for neuro/ psychiatric disease involvement?
1	TXNIP	Thioredoxin interacting protein	Cytoplasm	Yes (PMID - 23958415, 22661500)	Yes (AD) - 22482078
2	CP	Ceruloplasmin (ferroxidase)	Astrocytes	Yes (18977241, and 1761530 – cognitive aging)	Yes (PD - 16150804, MS - 23868451)
3	HIST1H4C	Histone cluster 1, H4c	Nucleous	Yes (susceptible, (20800603)	
4	MPZL2 (EVA)	Myelin protein zero-like 2	Membrane, cytoskeleton		
5	VWF	Von Willebrand factor	Endoplasmatic reticulum, extracellular matrix		Yes (22120183)
6	CD163	Macrophage-Associated Antigen	Cell membrane/ Monocytes and Macrophages		Yes (MS, 21737148)
7	SGPP1	Sphingosine-1-phosphate phosphatase 1	Endoplasmic reticulum membrane		Yes (Schizophrenia, 18683247)

8	FLJ35776 (DLGAP1-AS1)	DLGAP1 antisense RNA 1	Uncharacterized * LncRNA		
9	VSIG4	V-set and immunoglobulin domain containing 4	Macrophages		

Supplementary Table 4| Cross regional human aging altered genes, related to Figure 3

This supplemental table is included within this supporting information file. The cross-regional aging altered genes that were found as significantly altered in all the analyzed 10 brain regions upon aging. The table includes the full gene names, literature evidence for involvement in aging (if any) and cellular specificity. Reference in the main paper text: page 13, line 21.
